# ABI5-binding proteins are substrates of key components in the ABA core signaling pathway affecting seeds

**DOI:** 10.1093/plphys/kiaf674

**Published:** 2025-12-23

**Authors:** Tim J Lynch, B Joy Erickson McNally, Teodora Losic, Jonas Lindquist, Ruth Finkelstein

**Affiliations:** Department of Molecular, Cellular and Developmental Biology, University of California at Santa Barbara, Santa Barbara, CA 93106, USA; Biomolecular Science and Engineering Program, University of California at Santa Barbara, Santa Barbara, CA 93106, USA; Department of Molecular, Cellular and Developmental Biology, University of California at Santa Barbara, Santa Barbara, CA 93106, USA; Department of Molecular, Cellular and Developmental Biology, University of California at Santa Barbara, Santa Barbara, CA 93106, USA; Department of Molecular, Cellular and Developmental Biology, University of California at Santa Barbara, Santa Barbara, CA 93106, USA

## Abstract

The central components of the ABA core signaling pathway are families of receptors, clade A type 2C protein phosphatases (PP2Cs), SNF1-related protein kinases (SnRK2s), and diverse sets of proteins regulated by phosphorylation via these kinases, including basic leucine zipper (bZIP) transcription factors such as ABA-INSENSITIVE(ABI)5. The larger network of ABA signaling factors includes additional kinases and E3 ligases that modify these components to affect their activity and stability. ABI5-binding proteins (AFPs) are negative regulators of ABA response, and this study shows that *Arabidopsis thaliana* AFPs interact with specific family members of all components of this pathway and are substrates for SnRK2s and PP2Cs. AFPs also interact with subsets of MAP kinases (MPKs) and 14-3-3 proteins previously found to regulate the activity of the ABI5-related clade of transcription factors. Residues predicted to be phosphorylated are conserved between AFPs, but are located within regions predicted to be unstructured. ABA promotes phosphorylation of AFP2, but conditions that prevent phosphorylation of AFP2 result in decreased stability, a shift in localization toward dispersed foci, and reduced effectiveness for inhibiting ABA response at germination. Thus, AFP2 appears to be an important hub in the ABA core signaling pathway.

## Teaser

An inhibitor of ABA-INSENSITIVE5 is proposed to act as a hub in ABA signaling, and its stability, localization, and function are regulated by changes in phosphorylation state.

## Introduction

The phytohormone ABA regulates diverse responses in plants, in part mediated by large changes in gene expression. Although transcriptome experiments in diverse tissues at various stages of growth have identified many thousands of ABA-regulated genes, very few of these are consistently ABA responsive across all of these conditions (reviewed in Finkelstein 2013) and most transcriptome comparisons focus on co-expressed genes to construct gene regulatory networks acting in specific responses or tissues (reviewed in Ko and Brandizzi 2020). However, some loci encode core components regulating these diverse responses. The central components of the ABA core signaling pathway are families of receptors, protein phosphatases, protein kinases, and diverse sets of proteins regulated by phosphorylation via these kinases (Cutler et al. 2010; Ali et al. 2020; Chen et al. 2020). In the absence of ABA, PP2C clade A protein phosphatases bind to SNF1-related protein kinase2s (SnRK2s), sterically preventing them from becoming phosphorylated and active. When ABA is present, PYRABACTIN RESISTANT (PYR/PYL)/REGULATORY COMPONENT OF ABA RECEPTOR (RCAR) receptors bind and inactivate these PP2C phosphatases, thereby permitting phosphorylation and activation of the SnRK2 class kinases. These kinases can then phosphorylate regulatory proteins such as the ABA- INSENSITIVE(ABI)5/ABA RESPONSE ELEMENT BINDING FACTOR (AREB/ABF) clade of bZIP transcription factors, leading to ABA-induced changes in gene expression, including feedback regulation by inducing expression of the PP2C co-receptors (Wang et al. 2019). Several of these bZIP proteins are bound in a phosphorylation-dependent manner by 14-3-3 proteins, and this interaction can be required for gene activation by the bZIP factor (Schoonheim et al. 2007; Camoni et al. 2018).

The clade A PP2Cs are monomeric Ser/Thr phosphatases that were initially identified genetically by dominant mutations in the *Arabidopsis thaliana ABA-INSENSITIVE(ABI)1* and *ABI2* loci (Koornneef et al. 1984), resulting in ABA resistance due to their inability to be inactivated (Park et al. 2009; reviewed in Finkelstein 2013). Additional members of the clade, eg *ABA-HYPERSENSITIVE GERMINATION(AHG)1* and *AHG3*, were identified based on loss of function mutations conferring hypersensitivity to ABA, reflecting their role as negative regulators of ABA response (Yoshida et al. 2006; Nishimura et al. 2007). These PP2Cs have overlapping expression patterns and functions, such that double mutants are more hypersensitive than the single mutants. Interactome and other studies have identified numerous substrates or other interaction partners for these PP2Cs, including members of multiple families of kinases and transcription factors involved in response to ABA and abiotic stresses (Lumba et al. 2014; Yilmaz et al. 2022).

The SnRK2 Ser/Thr kinases are a plant-specific subfamily of the SnRKs, comprising 10 family members in *A. thaliana* (reviewed in Kulik et al. 2011 and Maszkowska et al. 2021). Although all SnRK2s function in stress responses, this subfamily is further divided into 3 groups distinguished by their response to ABA. Group 1 does not respond to ABA, Group 2 shows minimal response to ABA, and Group 3 is strongly activated by ABA (Kobayashi et al. 2004). In Arabidopsis, Group 3 consists of SnRK2.2, SnRK2.3, and SnRK2.6. These kinases have partially redundant functions, with SnRK2.2 and SnRK2.3 playing major roles in seed dormancy, control of germination and seedling growth, while SnRK2.6 has greater effects on stomatal regulation (Fujii and Zhu 2009; Nakashima et al. 2009). Triple mutants defective in all 3 are highly resistant to ABA and exhibit extreme drought sensitivity and severely stunted growth (Fujita et al. 2009). In contrast, SnRK2.10 is a member of Group 1 and responds to osmotic stress, but is not part of the ABA core signaling pathway (Kobayashi et al. 2004).

Mitogen activated protein kinases (MAPKs, also known as MPKs) are another conserved family of Ser/Thr kinases, some of which regulate abiotic stress and ABA response. MAPKs are the final step in the MAPK cascade, composed of MAP kinase kinase kinases (MAPKKKs/MAP3Ks/MEKKs) that phosphorylate and activate MAP kinase kinases (MAPKKs/MAP2Ks/MEKs), which in turn phosphorylate/activate the MAPKs (Jagodzik et al. 2018). Over 100 genes encode members of these families in *A. thaliana*, regulating diverse processes with substantial overlaps in function. Although not part of the ABA core signaling pathway, cross-talk between these pathways occurs at various levels: SnRK2s phosphorylate some MAPKs (Umezawa et al. 2013), while MAP3Ks phosphorylate SnRK2s and are required for their activation by ABA (Takahashi et al. 2020). MAPK cascades regulate ABA response through effects on gene expression, including induction of transcription factors such as ABI3 and ABI5 (Lee et al. 2014), and activation or stabilization of other factors, eg ABI4, by phosphorylation (Guo et al. 2016; Eisner et al. 2021; Maymon et al. 2022). The 20 MAPKs of Arabidopsis are divided into 4 clades (A to D), distinguished by their domain structures and the sequence at the site phosphorylated by MAPKKs, which is different in clade D (Group et al. 2002). Clade D also lacks a “common docking” domain that is recognized by MAPKKs, phosphatases, and other proteins. The most extensively studied MAPKs are MPK3, MPK4, and MPK6, which are members of clades A and B; these have been shown to function in diverse pathways including stomatal development, root growth, pollen tube growth, immune response, and response to abiotic stress and/or ABA (Wang et al. 2007; Galletti et al. 2011; Guan et al. 2014; de Zelicourt et al. 2016; Shao et al. 2020; Zou et al. 2021). All members of the C clade are also activated by ABA by inducing synthesis of the upstream MAP3Ks via the PYR/PYL/RCAR-PP2C-SnRK2 core signaling pathway (Danquah et al. 2015). Within this clade, MPK7 has been specifically implicated in dormancy release (Chen et al. 2023).

Although not initially characterized as part of the core signaling pathway, a group of ABI5-binding proteins (AFPs) identified by a yeast 2-hybrid screen using ABI5 as bait were shown to be important ABA-inducible negative regulators of ABA response (Garcia et al. 2008). One of these, AFP3, is encoded by one of the few genes that are ABA-induced in many different contexts, but especially during vegetative growth (Finkelstein 2013). In contrast, AFP1 and AFP2 are most active during germination and seedling establishment. The AFPs have 3 highly conserved domains, including an ethylene-responsive element binding factor-associated amphiphilic repression (EAR) domain in the “A domain” and a nuclear localization signal in the “B domain,” in addition to the C-terminal domain initially shown to interact with the ABI5/ABF/AREB transcription factors (Garcia et al. 2008). Overexpression of the AFPs results in extreme ABA resistance, including decreased expression of many seed maturation genes, failure to acquire desiccation tolerance, and ability to germinate on 100-fold higher ABA concentrations than wt seeds (Lynch et al. 2017).

Studies addressing the mechanism by which AFPs inhibit ABA response have demonstrated interactions with co-repressors including TOPLESS/TOPLESS-RELATED (TPL/TPR) and histone deacetylase (HDAC) subunits, suggesting effects on chromatin remodeling (Lynch et al. 2017). Although mechanistically more obscure, the AFPs also interact directly with DELLA proteins, antagonizing their inhibitory effects on germination (Finkelstein and Lynch 2022). The AFPs have also been proposed to confer ABA resistance by promoting ABI5 degradation, functioning as adaptors for E3 ligases that ubiquitinate ABI5 and possibly related bZIP proteins, based in part on colocalization of AFP1 with ABI5 and COP1 in nuclear bodies (Lopez-Molina et al. 2003). However, the interactions with several such E3 ligases (DWA1, DWA2, and KEG1) are rather weak and loss of function mutations for these ligases do not block the ABA resistance conferred by AFP2 overexpression (Lynch et al. 2022). Furthermore, tests of the time course of germination show that germination mostly precedes degradation of ABI5 (Lynch et al. 2022) and COP1 promotes ABI5 accumulation in the dark (Peng et al. 2022).

Outside of the conserved domains, much of the AFP sequences are predicted to be intrinsically disordered (Lynch et al. 2017; Varadi et al. 2023) (Supplementary Figure S1), raising the possibility that the AFPs can assume a variety of structures due to post-translational modifications, consistent with the diversity of their observed protein interactions. Furthermore, much recent work highlights the tendency of intrinsically disordered regions to drive phase transitions forming bimolecular condensates (Liu et al. 2023). The current work reports interactions between AFPs and the PP2Cs, SnRK2s, and MPKs previously shown to regulate ABA response and addresses the functional significance of these interactions. Additional interactions with ABA receptors and 14-3-3 proteins may affect the activity of signaling complexes containing some of these factors.

## Results

### Interactions between AFPs and components of the ABA core signaling pathway

**Figure 1.**
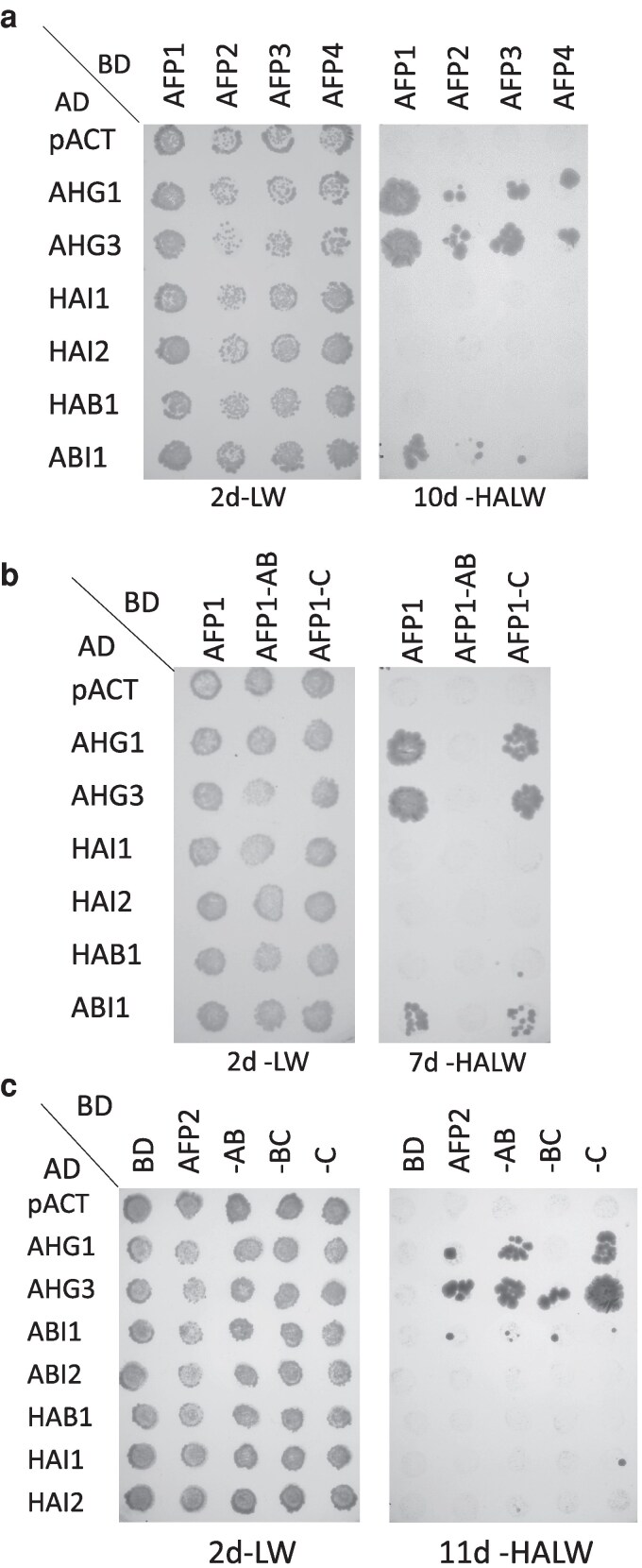
Interactions between AFPs and PP2Cs detected by yeast 2-hybrid assays. Fusions were combined by matings between Y187 carrying AD fusions and PJ69-4a carrying BD fusions. After overnight incubation on YPD media, yeast were replica plated onto selective media lacking leu and trp (-LW) to maintain the AD- and BD fusion plasmids and lacking histidine and adenine (-HALW) to score interactions between the fusion proteins. BD fusions to full-length AFPs paired with AD fusions to PP2Cs a). AD-PP2C fusions paired with BD fusions to domains of AFP1 b) or AFP2 c).

Our early studies of the AFPs revealed no obvious biochemical function, so we used AFP2 as bait in a yeast 2-hybrid screen to identify additional interacting partners that might provide clues regarding function. This screen produced a diverse set of potential interactors including multiple ABI5/ABF/AREB clade bZIP proteins, ABI clade protein phosphatases (PP2Cs), and a variety of kinases. Further specific pairwise yeast 2-hybrid assays, expanding this study to include all 4 members of the AFP family and 7 members of the ABI PP2C clade, showed strong interactions between all AFPs and AHG1 and AHG3. When the AFPs were present as binding domain (BD) fusions, additional interactions were limited to either AFP1 or AFP2 for only one other PP2C tested, ABI1 (Fig. 1a). Interactions mapped to the C domain of AFP1, but to several regions of AFP2 (Fig. 1b, c).

**Figure 2.**
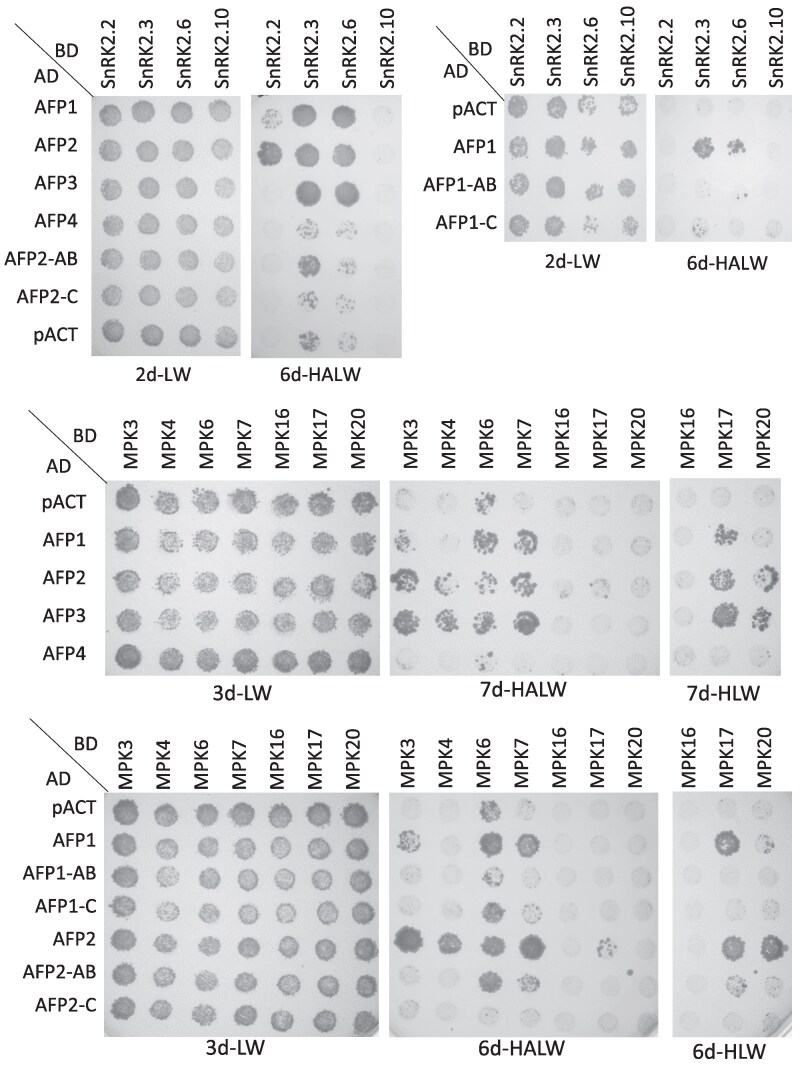
Interactions between AFPs and kinases detected by yeast 2-hybrid assays. The indicated fusions were combined and interactions were scored as described in Fig. 1.

Tests of interactions with kinases showed all AFPs except AFP4 interacted with SnRK2.3, SnRK2.6, MPK3, MPK4, and MPK7, but not with SnRK2.10 (Fig. 2). The BD-MPK6 fusion conferred growth without requiring an AD interaction partner, but reporter activation was again enhanced by interaction with AD fusions to AFP1, AFP2, and AFP3 (Supplementary Figure S2). Surprisingly, activity was impaired when BD-MPK6 was co-expressed with AFP4 or AFP2-C. Only AFP2 interacted with SnRK2.2 in this assay. Interactions with subdomains were very weak, suggesting that regions across the entire protein were required for these interactions. Although several D clade MPKs are highly co-expressed with the AFPs in maturing, dry, and germinating seeds (Supplementary Figure S3), only MPK17 and MPK20 interacted with the AFPs in yeast, and the interactions were relatively weak.

**Figure 3.**
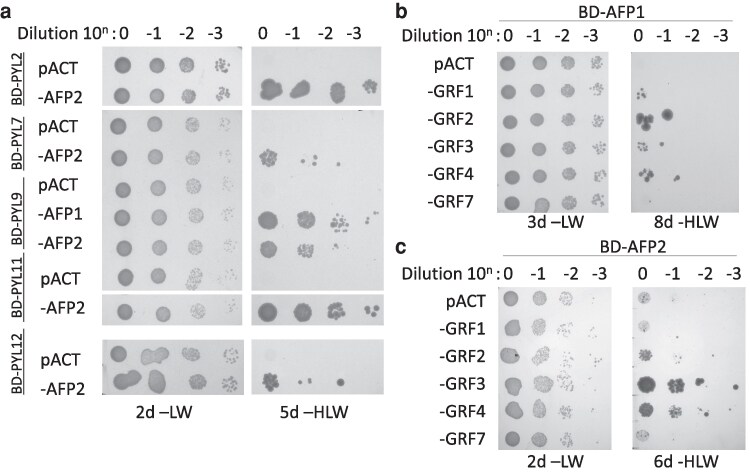
Interactions between AFPs, ABA receptors, and GRFs detected by yeast 2-hybrid assays. Following matings, the indicated diploid lines were grown overnight in media lacking leu and trp. After measuring the OD600, cultures were diluted to the same concentration, then serially diluted 10-fold 3 times. The diluted cultures were then replica spotted onto selective media. The -LW plate serves as a control for accuracy of the dilutions. Growth on the -HLW plate requires interaction between the AD and BD fusions to complement the *his3-200* allele of these yeast. Interactions between AD-AFPs and BD receptors (PYLs) a), AD-GRFs (14-3-3 proteins), and BD-AFP1 b) or BD-AFP2 c).

In contrast to the strong interactions between AFPs and bZIPs, PP2Cs and kinases, relatively weak interactions were observed between AFPs and a few members of the PYR/PYL/RCAR family of ABA receptors: PYLs 2, 7, 9, 11 and 12 (Fig. 3a; Supplementary Figure S4). All of these are expressed in maturing, dry, and/or imbibing seeds, coinciding with peak expression of AFP1, AFP2, and ABI5 (Supplementary Figure S5). Furthermore, PYLs 11 and 12 promote ABI5-mediated ABA response during germination and their expression is regulated by ABI5 (Zhao et al. 2020), and PYLs 7 and 9 are members of the small subset of receptors that can interact with AHG1 (Tischer et al. 2017). This result suggests that several AFPs can interact with all components of the ABA core signaling pathway.

Many phosphorylation-dependent protein activities, including hormone signaling, are regulated by 14-3-3 proteins that bind to phosphorylated Ser or Thr residues within specific contexts (Camoni et al. 2018). The Arabidopsis genome encodes 13 expressed 14-3-3 proteins, designated GENERAL REGULATORY FACTORS (GRFs), many of which are also expressed in maturing, dry, and germinating seeds (Supplementary Figure S5). Previous studies in multiple cereal grains and Arabidopsis have shown a role for 14-3-3 proteins in ABA-regulated transcriptional complexes, including direct interactions with members of the ABI5/ABF clade of bZIPs (Jaspert et al. 2011). We initially tested the Arabidopsis 14-3-3 proteins with the highest homology to the barley and rice proteins shown to interact with HvABI5 and HvABF1 (Schoonheim et al. 2007), and that are expressed in dry, imbibed, or germinating seeds: GRF1 (14-3-3 chi), GRF2 (14-3-3 omega), GRF3 (14-3-3psi), GRF4 (14-3-3 phi), and GRF7 (14-3-3 nu). AD fusions with GRF1, GRF2, GRF3, and GRF4 all interacted weakly with a BD-AFP1 fusion, but only the AD-GRF3 and AD-GRF4 fusions interacted above background with BD-AFP2 (Fig. 3b and c). Interaction with ABI5 was substantially stronger (Supplementary Figure S6). Consistent with the importance of phosphorylation for 14-3-3 binding, a BD fusion to AFP2 with alanine substitution mutations in 2 binding sites predicted by 14-3-3-Pred (https://www.compbio.dundee.ac.uk/1433pred/)  (Madeira et al. 2015) (ie, AFP2(2xA)) had greatly reduced interactions with the GRFs (Supplementary Figure S6). In addition to their relatively strong interactions with AFP1 and AFP2, transcripts for both GRF3 and GRF4 are present at similar levels to those of these AFPs in dry and imbibed seeds (Supplementary Figure S3), so our further studies focused on interactions with these family members.

We previously found that AFP1 and AFP2 could form homo- and heterodimers in yeast 2-hybrid and BiFC assays (Lynch et al. 2017). Attempts to map the interacting domains by further yeast 2-hybrid assays showed that the AB domain was sufficient for interactions with full-length AFP2, but not with itself, implying that multiple domains are required for interaction (Supplementary Figure S7a). Consistent with this, Alphafold modeling suggests that the most likely interface between AFP2 subunits includes residues present in the B and C domains (Supplementary Figure S7b).

**Figure 4.**
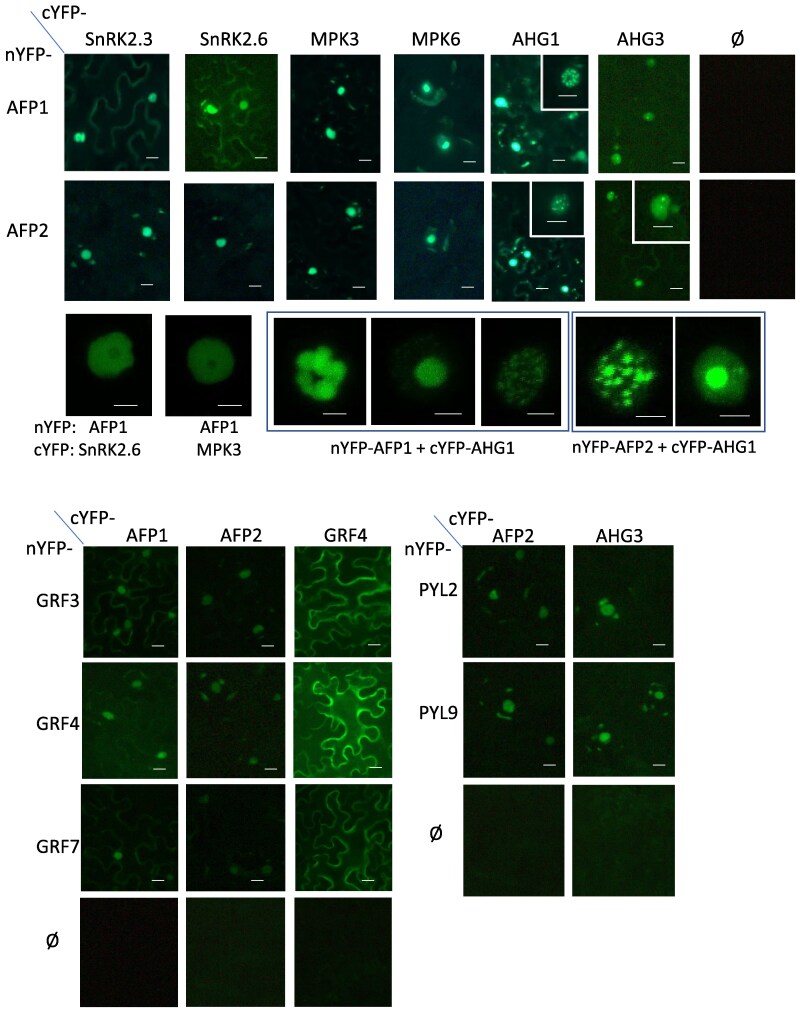
Interactions between AFPs and SnRK2s, PP2Cs, GRFs, and receptors detected by split YFP assays. Low magnification images were obtained with an Infinity camera on an Olympus AX70 epifluorescence microscope at 100× magnification. Insets are 400× magnification. High magnification images of individual nuclei were obtained with a Leica SP8 Resonant Scanning Confocal microscope. Scale bars = 10 µm for interactions with kinases, 5 µm for interactions with AHG1 The 2 images shown for cYFP-AFP2/∅ are the same.

The interactions between AFP1, AFP2, PP2Cs, kinases, receptors, and GRFs were further tested by BiFC assays, using split YFP fusions in transiently transformed *Nicotiana benthamiana*. These studies showed interactions between both AFPs and SnRK2.3, SnRK2.6, MPK3, MPK6, AHG1, AHG3, GRF3, GRF4, PYL2, and PYL9, confirming the yeast 2-hybrid results, but interactions with GRF7 and AHG3 were much weaker in these assays (Fig. 4). AFP2 also interacted with MPK4 and MPK7 in this assay. Although none of the 14-3-3 proteins showed any specific interaction with PP2Cs or a SnRK that interacts with AFP2 in the yeast 2-hybrid assay, GRF4 interacted strongly with MPK3 and SnRK2.6, and very weakly with AHG1, in BiFC assays (Supplementary Figure S6). Further mapping of the BiFC interactions between domains of the AFPs with AHG1 and SnRK2.3 showed that all AFP domains interacted with AHG1 (Supplementary Figure S8) (Erickson McNally 2016). In contrast, SnRK2.3 interacted with only the A and B domains of AFP1.

The BiFC assays also showed some differences in localization. Fluorescence was generally diffuse throughout the nuclei when AFPs and kinases were co-expressed, but localized to nuclear punctae when AFPs and AHG1 were combined (Fig. 4; Supplementary Figure S8). Although GRF4 was distributed throughout the cell when forming homodimers, or heterodimers with GRF3 and GRF7, the GRF interactions with AFPs were mostly limited to nuclei (Fig. 4).

### AFPs are substrates for SnRK2s and PP2Cs

**Figure 5.**
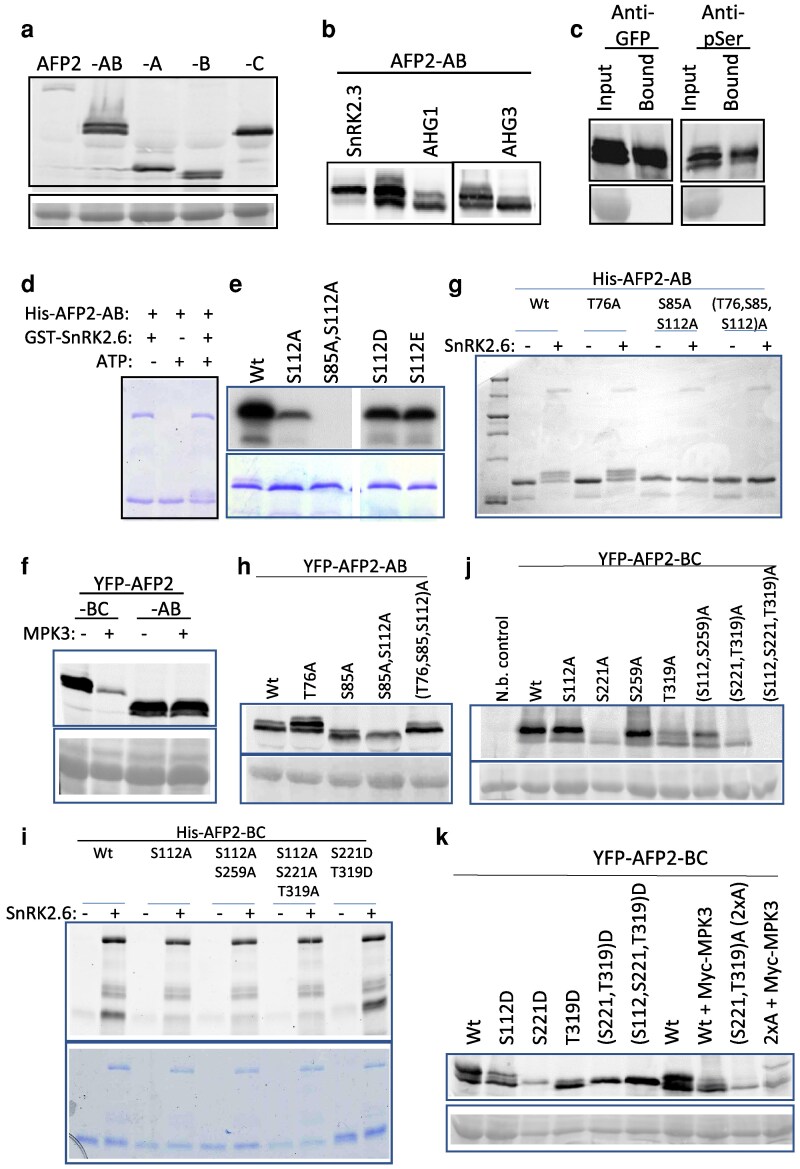
AFP2 is a substrate for both specific kinases and PP2Cs. a) Immunoblot of protein extracts from *N. benthamiana* leaves with transiently expressed full-length or subdomains of AFP2 as YFP fusions, detected by anti-GFP antibody; lower panel is Ponceau stain focused on Rubisco as loading control. b) Immunoblot of YFP-AFP2-AB fusion transiently co-expressed in *N. benthamiana* with fusions to either SnRK2.3 or AHG1. c) Transiently expressed YFP-AFP2-AB was immunoprecipitated using GFP-Trap agarose beads; upper panels are immunoblots with indicated antibodies, and lower panels are Ponceau stain. In vitro kinase reactions with GST-SnRK2.6 and wt or phosphomutant His fusions of AFP2-AB resulting in reduced mobility of the fusion protein d) detected by Coomassie stain, e) incorporation of radioactivity, lower panels are Coomassie stain of gel used in autoradiograph, or g) reactivity with ProQ Diamond stain. Immunoblots of protein extracts from leaves transiently expressing YFP fusions of f) the wt AB and BC domains of AFP2 with Myc-MPK3, h) wt and phosphomutant AFP2-AB domain fusions, j) wt and phosphomutant AFP2-BC domain fusions, and k) wt and phosphomimetic AFP2-BC domain fusions, including some co-expressed with Myc-MPK3. YFP fusions were detected by anti-GFP antibody; lower panels are Ponceau stain. i) In vitro kinase reactions with GST-SnRK2.6 and wt or mutant His6 fusions of AFP2-BC, viewed with ProQ Diamond stain; lower panel is subsequent Coomassie stain of same gel.

We had previously observed that YFP-AFP2 fusions migrate as doublets and occasionally even triplets on SDS-PAGE, suggesting the possibility of post-translational modifications (Erickson McNally 2016). These doublets are especially pronounced in any fusions containing the B domain of AFP2 (Fig. 5a). To determine whether the observed interactions between AFP2 and these kinases and phosphatases reflected a substrate role of AFP2, these proteins were transiently co-expressed in *N. benthamiana*. Co-expression with SnRK2.3 shifted the YFP-AFP2-AB fusion to predominantly the slower mobility form, whereas co-expression with AHG1 or AHG3 resulted in a shift toward the higher mobility form (Fig. 5b). YFP-AFP2-AB affinity purified with GFP-trap beads cross-reacted with an anti-phosphoserine antibody, and this reaction was enhanced in the slower migrating form, confirming that the altered mobility was due at least in part to phosphorylation (Fig. 5c).

AFP2 contains 40 serines and 22 threonines, constituting nearly 18% of the total amino acids in AFP2, many of which are conserved between AFP1 and AFP2 (Supplementary Figure S9). Of these, 17 serines and 11 threonines were predicted to be phosphorylated by the PhosPhAt database (Zulawski et al. 2012), but only 9 serines and 1 threonine are predicted at high stringency by http://musite.sourceforge.net/ (Supplementary Table S1). PhosPhAt lists only 3 documented phosphorylated residues, following ionizing radiation stress and dependent on the ataxia telangiectasia-mutated (ATM) and ataxia telangiectasia-mutated and Rad3-related (ATR) kinases: T76, S232, and S234 (Roitinger et al. 2015). Of these, only T76 was predicted by both programs. Based on the consensus target sites of R-X-X-S and S-D for ABA-induced phosphorylation by SnRK2s (Wang et al. 2013), potential targets in AFP2 are S85, S112, S150, S187, and S259. Of these, only S85 and S112 are conserved between AFP1 and AFP2. Similarly, 2 consensus sequences, P-X-S/T-P and S/T-P, are highly abundant in protein substrates of MAPKs (Seger and Krebs 1995) such that potential targets of MAPKs are T76, S221, and T319 of AFP2, all of which are conserved between AFP1 and AFP2. Three consensus motifs have been proposed for 14-3-3 target binding sites, 2 of which are present in AFP2: S112 fits the mode I consensus and both S346 and T347 fit the C-terminal mode III consensus (reviewed in Camoni et al. 2018). The 14-3-3 Pred program scores S112 and S85 as highly likely binding sites, with relatively moderate scores for T16, S38, and S259.

We took 2 approaches to identifying actual phosphorylation sites: in vitro phosphorylation with purified recombinant proteins and site-directed mutagenesis of predicted phosphorylation sites. We focused on specific target sites of SnRK2s by incubating recombinant His6-AFP2-AB (aa1-149) fusions with GST-SnRK2.6 in vitro, resulting in a shift of the His-AFP2-AB fusion to a higher apparent molecular weight (Fig. 5d). Mass spectrometric analysis of this slower mobility form identified S112 as a phosphorylated residue (Supplementary Figure S10). In vitro kinase assays including gamma-labeled ATP, comparing wild-type (wt) to either S112A or phosphomimetic S112E or S112D mutants of His6-AFP2-AB, showed that phosphorylation by SnRK2.6 was still possible until the S85 was also converted to alanine (Fig. 5e). The phosphomimetic mutants appeared to have an intermediate degree of phosphorylation between the wt and S112A forms, suggesting that true phosphorylation at S112 affected the likelihood of additional phosphorylation. In contrast, a His6-AFP2-C fusion (aa-149-348) was not phosphorylated by SnRK2.6 (Supplementary Figure S11a).

Immunoblot analyses of possible in vivo phosphorylation by Myc-MPK3 showed either no shift in mobility for the YFP-AFP2-AB domain fusion or an apparent compression to the higher mobility form for the YFP-AFP2-BC domain fusion (Fig. 5f). Surprisingly, although neither the S85A, S112A double mutant nor the T76A, S85A, S112A triple mutant of the His6-AFP2-AB domain fusion could be phosphorylated by SnRK2.6 in vitro (Fig. 5g), only the double mutant ran as a single high mobility form when transiently expressed as a YFP fusion in planta (Fig. 5h). The triple mutant ran at the same mobility as wt when expressed as a YFP fusion in planta, and the T76A mutant of the His6-AFP2-AB domain fusion showed a greater shift toward lower mobility forms than the wt fusion (Fig. 5h). However, similar to the wt fusion, its mobility was not altered by co-expression with Myc-MPK3 (Supplementary Figure S11b).

In vitro kinase assays with His6-AFP2-BC domain fusions showed that an S112A substitution greatly reduced phosphorylation of this domain by SnRK2.6 (Fig. 5i). Similarly, S112A mutations blocked in planta phosphorylation of YFP-AFP2-BC fusions by SnRK2.3 when transiently co-expressed in *N. benthamiana* (Supplementary Figure S12a). Although phosphorylation of S112A in the in vitro reactions was not affected by phosphomimetic mutations in S221 and T319, these mutations reduced mobility of the fusion (Fig. 5i). Transiently expressed YFP-AFP2-BC mutant fusions converting S112, S259, S221, or T319 to alanine all still appeared as doublets in immunoblot analysis, as did double mutants combining predicted SnRK2 targets (S112, S259)A or potential MPK targets (S221, T319)A (Fig. 5j). However, both single and double mutants affecting S221 and T319 reduced the shift toward lower mobility, and both double mutants were under-accumulated relative to the wt. The triple mutant (S112, S221, T319)A fusion was rarely detectable. Unlike the alanine substitution mutants, YFP-AFP2-BC fusions with phosphomimetic mutations converting S221 alone or in combination with S112 and T319 to aspartic acid all resulted in compression to a single intermediate mobility form (Fig. 5k). Co-expression with MPK3 also converts most of the wt AFP2-BC fusion toward higher mobility forms, but has little effect on the (S221, T319)A mutant which already displays a higher mobility than the wt (Fig. 5f and k). In contrast to the higher mobility on Laemmli SDS-PAGE of AFP2-BC co-infiltrated with MPK3, analysis on Phos-tag gels showed a shift toward decreased mobility, reflecting increased phosphorylation (Supplementary Figure S12b). The S221A mutation alone blocked the phosphorylation seen in the wt BC domain, but a phosphorylated form reappeared in the (S221, T319)A mutant. Although the (S221, T319)A mutant migrated similarly to the wt BC domain, co-expression with MPK3 resulted in the appearance of a different phosphorylated form, visible in both gel systems, suggesting that loss of both “consensus” MPK targets led to phosphorylation of an as-yet unknown alternative residue, possibly by an unidentified endogenous kinase.

In contrast to the AB and BC domain fusions, full-length YFP-AFP2 transiently co-expressed with Myc-AHG1 in *N. benthamiana* shows as much or more phosphorylation than YFP-AFP2 alone, even when the SnRK2 and potential MPK target residues have been converted to alanines, again suggesting that alternative residues are being modified (Supplementary Figure S12d). Surprisingly, YFP-AFP2 co-expressed with Myc-SnRK2.3 appeared less phosphorylated than YFP-AFP2 alone. However, alanine substitutions in the SnRK target residues (S85 and S112) resulted in the appearance of different bands, reflecting both less and more phosphorylation, but including the additional S259A mutation fully blocked SnRK2-induced phosphorylation (Supplementary Figure S12d). Alanine substitutions in potential MPK target residues (S221 and T319) also blocked phosphorylation by SnRK2.3. Collectively, these results suggest that modification of the primary SnRK2 or MPK targets affects accessibility of the SnRK2 target sites and may lead to phosphorylation of additional sites.

### ABA effects on AFP2 phosphorylation state

**Figure 6.**
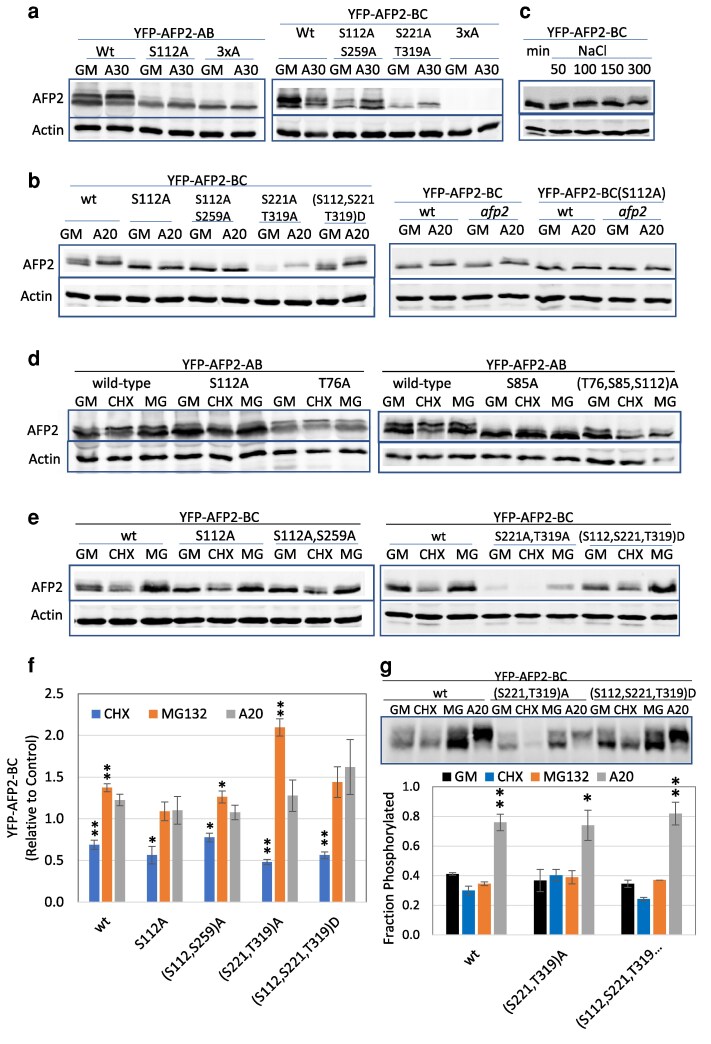
In vivo expression assays with AFP2 domain fusions testing relative significance of specific residues for ABA/stress-induced modification and stability. Immunoblots of extracts from a) the indicated YFP-AFP fusions transiently expressed in *N. benthamiana*, followed by 4 h incubation in GM ± 30 µM ABA; b and c) *A. thaliana* transgenic seedlings grown 5 d on GM, then incubated in b) GM ± 20 µM ABA for 4.5 h or c) min ±NaCl (50−300 mM) for 2.75 h; d) YFP-AFP2-AB fusions transiently expressed in *N. benthamiana*, followed by 6 h incubation in GM ± 20 µM CHX or MG132; e) *A. thaliana* transgenic seedlings grown 5 d on GM, then incubated in GM ± 20 µM CHX or MG132 for 6 h. All upper panels were probed with anti-GFP. Lower panels show either anti-actin reactivity or Ponceau stain (pink). f) Quantification of YFP-AFP2-BC protein with the indicated mutations from stable *A. thaliana* transgenic seedlings incubated in GM + 20 µM CHX, MG132, or ABA as shown in panels b) and e), normalized relative to actin as loading control, and expressed relative to the levels of each fusion protein following incubation in GM. Data displayed are the average of triplicate samples + Se. ** and * indicate statistically different from level in GM (*P* < 0.01 and *P* < 0.05, respectively, based on 2-tailed Student's *t*-test). g) Immunoblot of Phos-tag gel separating YFP-AFP2-BC from a subset of the extracts shown in panels b) and e). The fraction phosphorylated was calculated as the intensity of the upper band(s) relative to the sum of upper and lower band intensities. The average shown is based on quantification of replicate gels + Sd. ** and * indicate statistically different from all other treatments (*P* < 0.01 and *P* < 0.05, respectively, based on ANOVA with post hoc Tukey HSD test).

Given that ABA activates the Group 3 SnRK2s, we tested whether these mutations affected any post-translational modifications induced by ABA treatment. Transiently expressed wt YFP-AFP2-AB domain fusions shifted from roughly 40% slower mobility forms in control conditions to 60% slower mobility forms after 4 h exposure to 30 µM ABA (Fig. 6a). In contrast, the S112A mutation alone, or in combination with T76A and S85A (“3xA”), blocked this shift (Fig. 6a). Similarly, the highest mobility form of the YFP-AFP2-BC domain fusion shifted to an intermediate slower mobility following ABA treatment and this was blocked in the (S112, S259)A mutant. In contrast, the (S221, T319)A mutant of the BC domain continued to show a shift of the highest mobility form in response to ABA, but had greatly reduced expression of the slowest mobility form under any condition.

In stable transgenic Arabidopsis lines carrying the wt YFP-AFP2-BC domain fusion, this shift in mobility was induced by exposure to as little as 1 µM ABA for 6 h or by 1 h of treatment with 20 µM ABA (Supplementary Figure S13a and b). Similar to the transient expression assays, only those lines with the S112A mutation blocked the shift to slower mobility (Fig. 6b), suggesting that this residue is modified in response to ABA treatment. Phos-tag gel analysis showed that the ABA-induced shift in mobility correlated with S112-dependent changes in phosphorylation (Supplementary Figure S12c), but surprisingly, “phosphomimetic” Asp substitutions at S112, S221, and T319 (“3xD”) did not prevent phosphorylation of this fusion. A slight mobility shift of the YFP-AFP2-BC domain fusion was also seen in response to salinity stress imposed by at least 100 mM NaCl (Fig. 6c).

**Figure 7.**
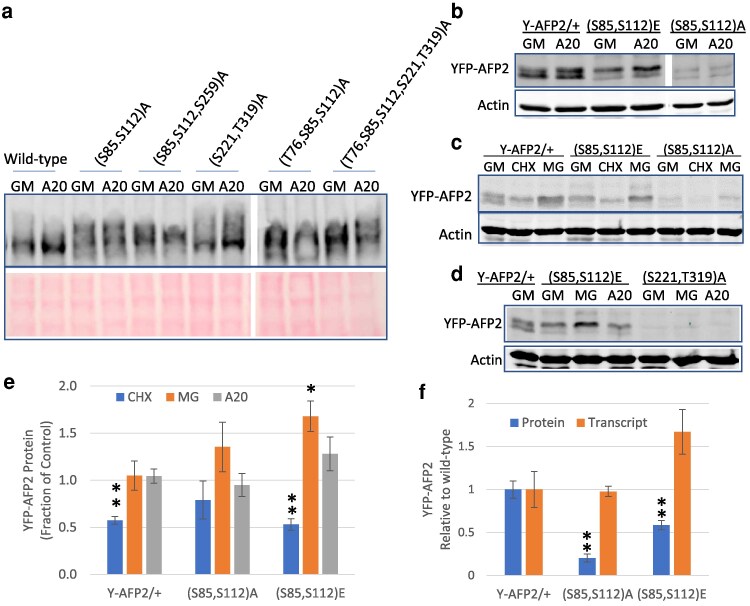
Expression assays with full-length AFP2 fusions testing relative significance of specific residues for ABA-induced modification and stability. a) Immunoblots of protein extracts from *N. benthamiana* leaves transiently expressing YFP fusions of full-length AFP2 with the indicated substitutions, following 6 h exposure to control media (GM) ± 20 µM ABA (A20), resolved on a Phos-tag gel. YFP-AFP2 fusions were detected with anti-GFP antibody; lower panel is Ponceau stain. b to d) Immunoblots of protein extracts from stable transgenic Arabidopsis 5-d-old seedlings with the indicated YFP-AFP2 fusions, following 6 h exposure to GM, supplemented with 20 µM ABA (A20) or 100 µM cycloheximide (CHX) or MG132 (MG). Proteins were resolved on 10% SDS-PAGE. e) Accumulation of fusion protein following treatment with CHX, MG132, or ABA, normalized relative to actin, expressed relative to level of that fusion in seedlings incubated on GM, as shown in b) to d). f) Relative protein and transcript levels of wt and mutant YFP-AFP2 fusions in 5-d-old seedlings of stable transgenic lines. YFP-AFP2 protein levels were normalized relative to actin. Transcript levels were normalized relative to the geometric mean of PP2AA3 (At1g13320) and AP2M (At5g46630) of Arabidopsis. Both are expressed relative to the average value for the wt construct. Data shown in e) and f) is average of at least triplicate samples, and error bars represent Se. ** and * indicate statistically different from control (*P* < 0.01 and *P* < 0.05, respectively, based on 2-tailed Student's *t*-test).

Although transiently expressed full-length YFP-AFP2 fusions showed no difference in phosphorylation between control and ABA treatments, there was generally a higher degree of phosphorylation in proteins with more ala substitutions, again consistent with modifications at different sites (Fig. 7a). In contrast, transgenic lines carrying the full-length YFP-AFP2 fusion showed an ABA-induced shift toward slower mobility/greater phosphorylation of the fusion (Fig. 7b). The stable transgenic lines with (S85, S112)A substitutions showed no change in fusion mobility in response to ABA, whereas the phosphomimic mutations (S85, S112)E displayed a shift toward slower mobility on control media.

Comparison of transcript levels for wt vs mutant fusions in tissue harvested in parallel to that used for protein extracts showed that levels were somewhat variable for all constructs, as expected for a transient system, but the reductions in protein levels of the mutant forms were far greater than any changes in transcript levels (Supplementary Figure S14). Although some transiently expressed transgene transcripts were 2- to 3-fold lower for the mutant constructs, this was not sufficient to explain the at least 10-fold decreases in YFP-AFP2-AB protein or the 5- to 10-fold decreases in YFP-AFP2-BC protein fusions (Supplementary Figure S14c). Stable transformants carrying the (S221, T319)A mutant YFP-AFP2-BC fusion also showed at least 5-fold less protein despite a similar transcript level as the wt fusion. Similarly, the YFP-AFP2-BC(S112A) mutant form showed a roughly 4-fold reduction in protein:transcript in stable transformants (Supplementary Figure S14e). All of these results are consistent with possible defects in stability or translation of the fusions. Protein levels were greatly reduced for full-length YFP-AFP2 fusions with ala substitutions in stable transgenic lines, but transcript levels were similar for wt and the (S85, S112)A variant (Fig. 7).

### Effects of phosphorylation on AFP2 stability

To further address the functional relevance of these differences in phosphorylation, we tested stability and potential proteasomal degradation by assaying accumulation following treatment with either the translation inhibitor cycloheximide (CHX) or the proteasome inhibitor MG132. In transient expression assays, the higher mobility form of the AB domain decreased from roughly 58% in control conditions to 50% of the total in CHX, but the high mobility form was stabilized by MG132 (Fig. 6d), consistent with proteasomal degradation of the high mobility/unphosphorylated form. Accumulation of the low mobility form was still seen in the T76A mutant, but was reduced in all the mutant fusions affecting SnRK2 targets: S85A, S112A, (S85, S112)A and the (T76, S85, S112)A triple mutant, consistent with a role for SnRK2-mediated phosphorylation in regulating protein stability. The S85A mutant appeared to have an even higher mobility than the wt fusion forms (Figs. 5h and 6d).

The YFP-AFP2-BC domain fusions were tested in both transient and stably transformed tissue. All stable transformants showed reduced fusion accumulation following CHX treatment and most increased accumulation in MG132-treated seedlings (Fig. 6e and f). The fusion combining S221A and T319A mutations had severely decreased accumulation in both stable and transiently transformed tissue (Fig. 6e and f; Supplementary Figure S14d and e). ABA treatment primarily stabilized accumulation of the phosphorylated form (Fig. 6g). Although the double mutant proteins were stabilized by MG132 treatment, this treatment was not sufficient to increase accumulation of the (S112, S221, T319)A (3xA) mutant in transiently transformed tissue (Supplementary Figure S15). Furthermore, the limited YFP-AFP2-BC(3xA) fusion visible was present in only a small fraction of cells, mostly in small punctae throughout the cytoplasm, not concentrated in the nuclei as for all other YFP-AFP2 fusion constructs (Supplementary Figure S15c).

In stable transformants with full-length wt YFP-AFP2, the phosphorylated form was more stable than the faster-migrating form (Fig. 7c). Consistent with this, lines with either alanine or phosphomimetic substitutions affecting S112 have substantially lower fusion protein levels, despite transcript levels that are similar or higher than those for the wt fusion (Fig. 7c and f). All of these full-length YFP-AFP2 fusions are proteasomally degraded, but the effects of CHX and MG132 are more limited for those with ala substitutions (Fig. 7c and e). In contrast, the only stable lines expressing the (S221, T319)A variant had extremely low fusion protein accumulation, which was not increased by MG132 treatment (Fig. 7d) and reflected very low transcript levels (less than 0.1% of that for the wt fusion transcript).

### Effects of phosphorylation on AFP2 localization

**Figure 8.**
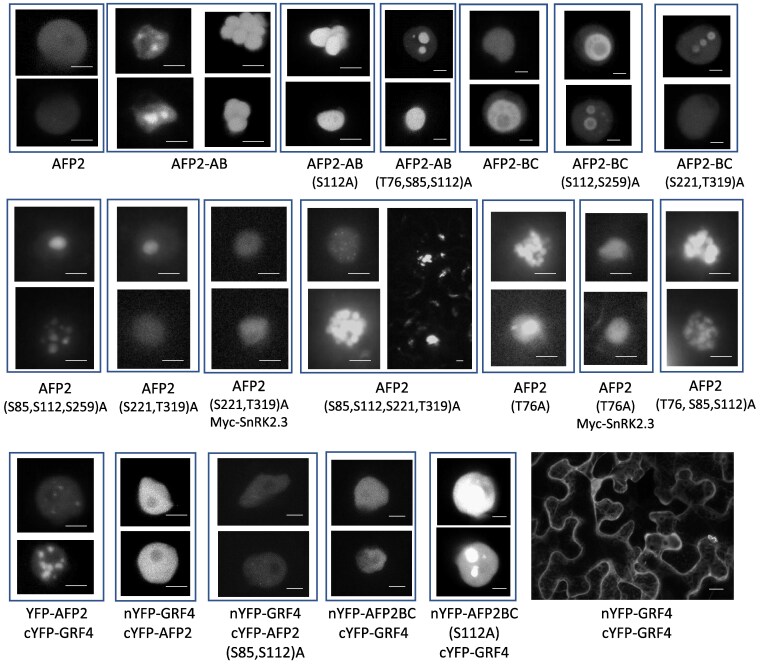
Localization of YFP fusions to wt and phosphomutant AFP2-AB and -BC domains, and split YFP assays with GRF4, in transiently transformed *N. benthamiana*. Confocal images of nuclei: scale bars = 5 µm. nYFP-GRF4/cYFP-GRF4 image: scale bar = 20 µm.

Having observed that co-expression of either nYFP-AFP fusions with cYFP-AHG1 or YFP-AFP2 with Myc-AHG1 led to formation of nuclear punctae we tested whether mutations preventing phosphorylation would lead to similar changes in localization in transient expression assays. Whereas full-length YFP-AFP2 fusions appear mostly diffuse through nuclei, but generally excluded from nucleoli, YFP-AFP2-AB domain fusions display as dense bundles of foci (Fig. 8). YFP-AFP2-AB(S112A) and (3xA) mutants appear to form fewer foci. Although not uniform across the leaf, the 2 double mutants of the YFP-AFP2-BC domain fusions tend toward more discrete areas of accumulation, appearing either more excluded from some regions (S112A, S259A) or more focused in a few foci (S221A, T319A), consistent with effects of phosphorylation state on changes in aggregation state.

Full-length YFP-AFP2 fusions with ala substitutions in S85, S112, and S259 are generally diffuse in the nuclei, but occasionally form condensates. Similar to their effects on the BC domain, the full-length (S221, T319)A mutants are also diffuse with occasional foci within nuclei, but become more diffuse when co-expressed with SnRK2.3. Combining the (S85, S112, S221, T319)A mutations in a full-length protein results in many dispersed nuclear foci and some punctae at the cell periphery. Fusions with ala substitutions in T76 alone are sufficient to produce multiple nuclear foci, but become diffuse when co-expressed with SnRK2.3. Combinations including T76A and ala substitutions in SnRK2 targets produce many nuclear foci, whether or not SnRK2.3 is co-expressed.

Complexes of signaling proteins may be scaffolded by 14-3-3 proteins such as the GRFs, which recognize phosphorylation in several consensus motifs. The S85 and S112 residues of AFP2 are both SnRK2 target sites and predicted binding sites for 14-3-3 proteins. Similar to the yeast 2-hybrid result, comparison of BiFC assays with GRF4 and either wt AFP2 or a (S85, S112)A mutant show greatly reduced interactions with the mutant (Fig. 8). However, GRF4 still interacts with the S112A mutant of the AFP2-BC domain, consistent with the potential for additional 14-3-3 binding sites in this domain. Unlike the nuclear interactions with AFP2, GRF4 dimers are primarily cytoplasmic.

### Effects of AFP2 phosphorylation on ABA sensitivity of germination

We previously showed that overexpression of YFP fusions to either full-length AFPs or just the BC domains were sufficient to confer resistance to ABA inhibition of germination in transgenic Arabidopsis, but AB-domain fusions had almost no effect (Lynch et al. 2017). The full-length AFP2 fusions conferred up to 100-fold reductions in ABA sensitivity and produced desiccation intolerant seeds, while the AFP2-BC domain fusions conferred up to 30-fold reductions in ABA sensitivity and did not impair seed viability. Arabidopsis carrying transgenes with mutations either blocking phosphorylation or creating phosphomimetics in the SnRK2 target sites were tested for effects on ABA sensitivity of germination.

**Figure 9.**
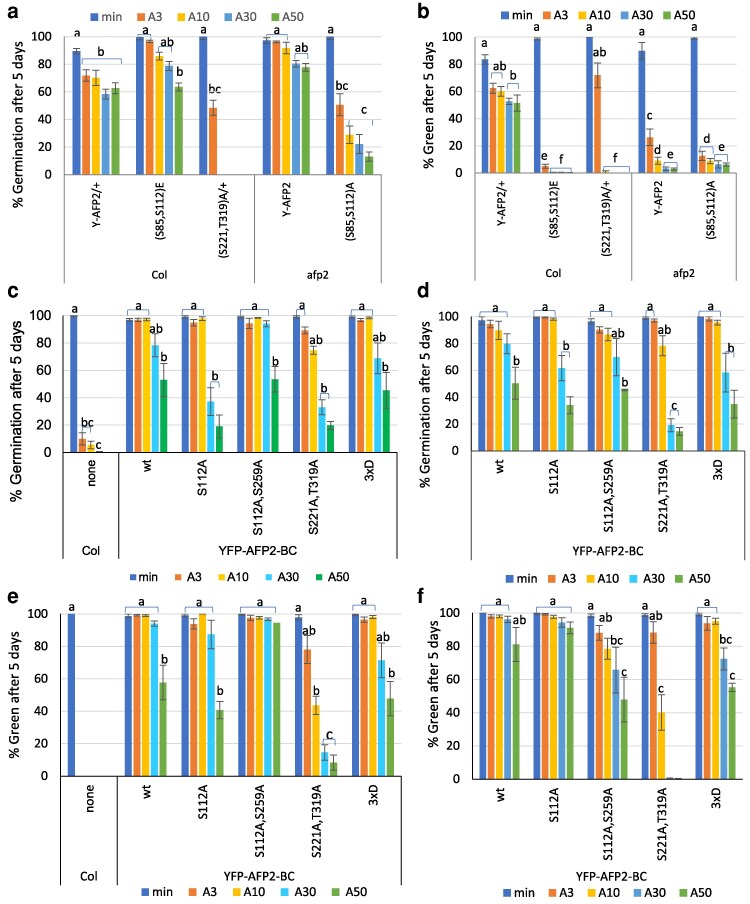
Physiological analysis comparing ABA sensitivity of germination or cotyledon greening of transgenic lines overexpressing wt vs phosphomutant or phosphomimetic AFP2 fusions. Seeds of the indicated genotypes in wt (Col) background a, b, c, and e) or *afp2* background a, b, d, and f) were stratified for 3 d, then incubated on minimal nutrient media with 0 (min), 3 (A3), 10 (A10), 30 (A30), or 50 (A50) µM ABA. Germination a, c, and d) and cotyledon greening b, e, and f) were scored after 5 d at 22 °C. Data displayed are the average of at least triplicate assays for each genotype and treatment ± Se. Bars with different letters represent statistically different values using Tukey's HSD post hoc test (*P* < 0.01).

Although overexpression of full-length AFP2 with a variety of mutations permitted germination on up to 50 µM ABA, lines carrying phosphomimetic (S85, S112)E or non-phosphorylatable (S85, S112)A mutant YFP-AFP2 fusions differed from those with the wt fusion in that seedling greening was still greatly impaired by any ABA (Fig. 9a and b; Supplementary Figure S16). Transgenic seeds overexpressing YFP-AFP2 with mutations in the C domain sites (S221, T319)A differed from those affecting the SnRK2 targets in that they were not able to germinate on media with ABA exceeding 3 µM, but greening was not impaired by this lower concentration of ABA (Fig. 9a and b). In contrast, transgenic seeds overexpressing YFP-AFP2 combining all of these ala substitutions (S85, S112, S221, T319)A blocked seed maturation, resulting in production of green seeds lacking desiccation tolerance (Supplementary Figure S17a and b). ABA sensitivity of germination of seeds expressing this quadruple mutant protein was tested by removing maturing seeds from developing siliques. Although also able to germinate on at least 50 µM ABA, subsequent seedling development was severely impaired even in the absence of ABA (Supplementary Figure S17c to e). The relatively weak ABA resistance of transgenic lines with the (S221, T319)A variant reflected the low level of fusion protein (Fig. 7; Supplementary Figure S17g). In contrast, the (S85, S112, S221, T319)A mutant constructs produced similar levels of fusion protein as the wt construct in seeds and were highly effective in blocking ABA response, but the mutant protein was not maintained during seedling growth (Supplementary Figure S17e to g).

The poor viability of seeds and seedlings expressing the full-length YFP-AFP2 resulted in partial silencing of these transgenes over generations and led us to focus on AFP2-BC domain fusions for additional mutant analyses. When overexpressed in a wt background, all YFP-AFP2-BC domain fusions conferred similar resistance to ABA (Fig. 9c). The well-expressed transgenes from the wt background were backcrossed into the *afp2* mutant to create isogenic lines with similar transgene expression levels (Supplementary Figure S13c), resulting in similar resistance to ABA as seen in the wt background up to 10 µM ABA (Fig. 9d). However, the mutants with alterations in the residues predicted to be phosphorylated by MPKs were less effective than the wt fusion in permitting germination on ABA higher than 30 µM in this background (Fig. 9d). Comparison of protein levels showed that only the (S221, T319)A mutant had reduced fusion protein accumulation, but accumulation could be increased by MG132 treatment (Fig. 6e).

Germination sensu stricto is defined as complete when the radicle emerges from the seed coat and is usually followed by cotyledon greening during seedling establishment in the light. However, some ABA resistant lines including *abi5* mutants and AFP overexpressors often reverse this order when germinating in the presence of ABA, such that their cotyledons turn green prior to radicle emergence. Consequently, at the higher ABA concentrations, the fraction of seeds that have turned green may be higher than the fraction that has completed germination (Fig. 9e and f). This was especially true for seeds expressing the wt and S112A mutants of the YFP-AFP2-BC fusion in the *afp2* mutant background. In contrast, the (S221, T319)A mutant was less effective than the other constructs in reducing ABA sensitivity in the *afp2* background and severely delayed greening when germinating on ABA.

### Effects of AFP2 phosphorylation on seed maturation

**Figure 10.**
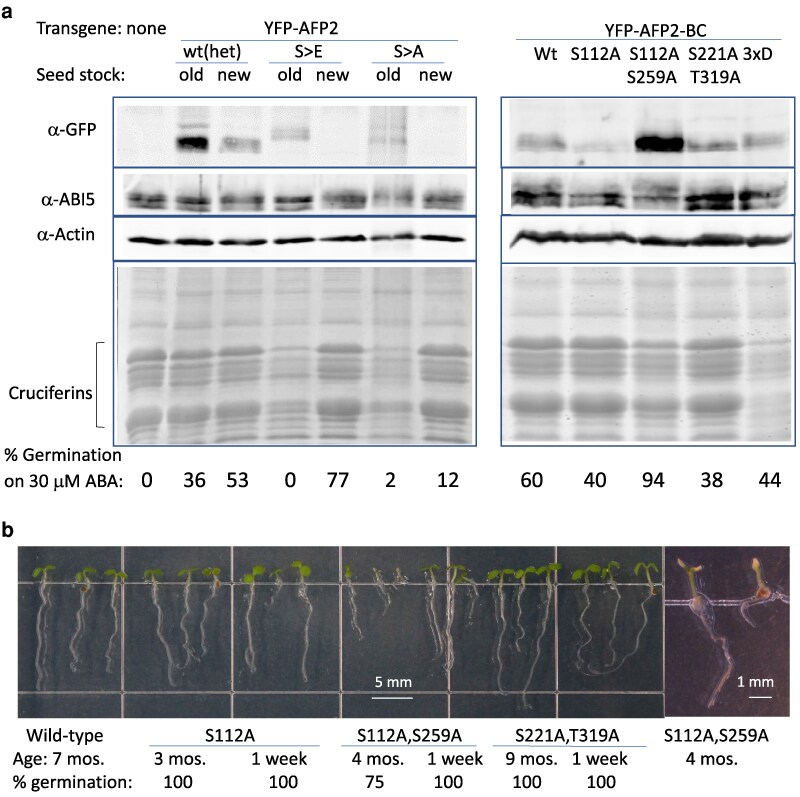
Effects of phosphomutant or phosphomimetic YFP-AFP2 fusions on storage protein accumulation and seed longevity. a) Comparison of dry seed protein extracts from lines either segregating a full-length wt AFP2 fusion or homozygous for S85E, S112E (S > E) or S85A, S112A (S > A) mutant full-length AFP2 fusions (left) or AFP2-BC fusions with the indicated mutations (right). Upper panels show immunoblots of YFP-AFP2 (-BC) fusions detected by α-GFP, -ABI5, and -Actin as a loading control. Lower panels show Coomassie staining of a comparably loaded higher 15% SDS-PAGE to retain and resolve the storage proteins. The “old” extracts were from early generations of these lines with high transgene expression and poor seed viability, while “new” extracts were from later generations with partially silenced transgenes permitting better viability but still conferring ABA-resistant germination. b) Seedlings from seed stocks of the indicated ages and genotypes, and their % germination, after 5 d growth on minimal medium.

In addition to decreasing ABA sensitivity at germination, AFP2 overexpression inhibits various aspects of seed maturation including accumulation of storage proteins (eg cruciferins) and some late embryogenesis abundant (LEA) proteins (Lynch et al. 2022). To determine whether the phosphorylation state of AFP2 was important for this regulation, we compared storage protein accumulation in wt vs transgenic seeds with either wt or mutant AFP2 fusions. Surprisingly, even though most mutants were less effective than wt AFP2 for conferring ABA resistance, only the mutant forms with substitutions in multiple target residues resulted in a decrease in storage protein accumulation (Fig. 10; Supplementary Figure S17h). The most dramatic reductions in cruciferin accumulation were seen in seeds producing the AFP2 quadruple mutant (4xA = (S85, S112, S221, T319)A) (Supplementary Figure S17h).

The 2 BC domain mutant fusions producing the greatest reduction of storage protein accumulation ((S112, S259)A and “3xD”) had similar effects on ABA sensitivity of germination and seedling establishment (Figs. 9 and 10; Supplementary Figure S16). However, they differ in that the (S112, S259)A mutations also adversely affect seed longevity, resulting in decreased viability within 4 months and complete lethality by 6 months (Fig. 10; Supplementary Figure S18). It is noteworthy that the (S112, S259)A mutant form of AFP2-BC accumulates to higher levels in seeds than the wt construct (Fig. 10), suggesting that this form might have greater effects on disrupting seed maturation, but this difference fades away during seedling growth (Supplementary Figure S19).

Examination of multiple ages of seed lots producing different AFP2-BC fusion variants showed that the major differences in storage protein levels reflected loss during aging (Supplementary Figure S18). Furthermore, this was not directly related to the level of ABI5 accumulation in that lines with very little storage protein still had ample ABI5. ABI5 is activated by phosphorylation and already appears as a triplet in dry seeds of all genotypes, but shifts to a more highly phosphorylated state when incubated on ABA (Supplementary Figure S19).

## Discussion

### Post-translational modification of ABA core signaling components

**Figure 11.**
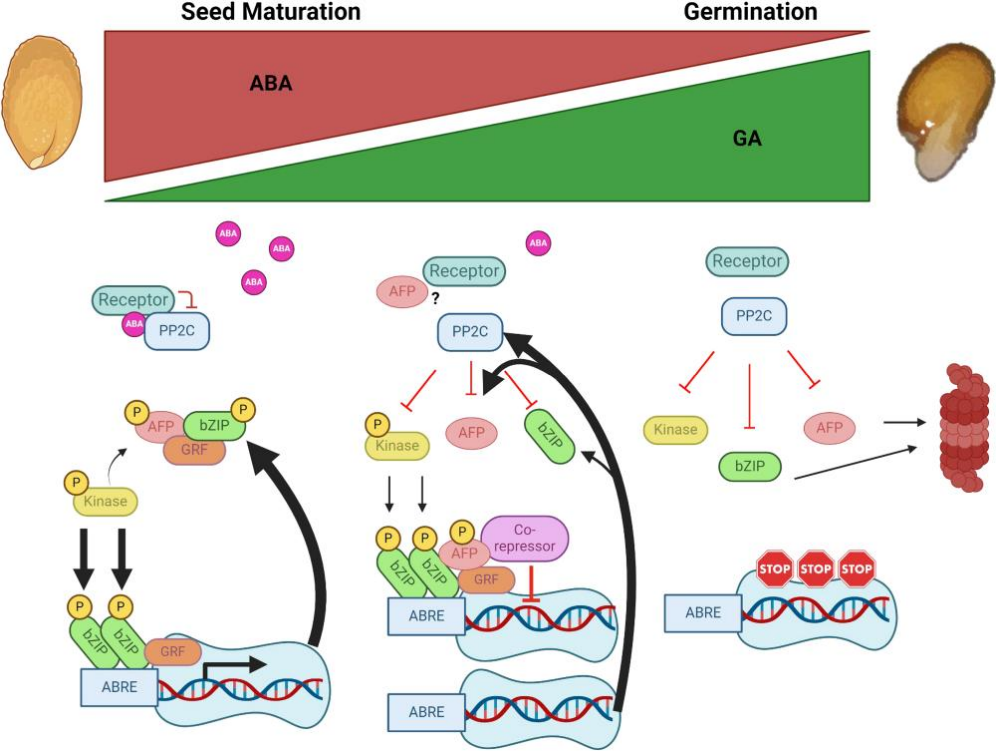
Changing relationships in the ABA core signaling pathway during the transition from seed maturation to germination. At high ABA concentrations, PP2Cs (eg AHG1 and AHG3) are inactive, kinases (eg SnRK2s and MPKs) are active, and bZIPs (eg ABI5 and ABFs) and AFPs are phosphorylated and are bound by GRFs (14-3-3 proteins). When the bZIP:AFP ratio is high, ABA-regulated gene expression proceeds and germination is blocked (Garcia et al 2008). As ABA levels decrease, ABA induces repressors including AFPs and PP2Cs in a feedback mechanism, the bZIP:AFP ratio decreases, and seed-expressed gene expression declines. Interaction between AFPs and co-repressors (eg TPL/TPR and HDAC components) leads to inactivation of seed-expressed genes (Lynch et al. 2017). At low ABA levels, the PP2Cs are active and inactivate the SnRK2s, bZIPs (Lynch et al. 2012), and AFPs, and the latter 2 classes are proteasomally degraded. Many additional interactors are not included due to space constraints. The figure was created with BioRender.com.

The core signalosomes of ABA signaling comprise the PYR/PYL/RCAR receptors, clade A PP2C protein phosphatases, and SnRK2 kinases (reviewed in Lim et al. 2022; Née and Krüger 2023). Each of these components is encoded by a multigene family that is differentially expressed, with different interaction characteristics and ABA sensitivities, creating the potential for a range of responses at different ABA concentrations in different tissues (Tischer et al. 2017). In the canonical view, ABA bridges the receptors with PP2Cs, effectively inactivating the PP2Cs and releasing the SnRK2s to become phosphorylated and active (Fig. 11). When active, the SnRK2 kinases regulate the activity of many effector proteins, including the ABI5/ABF/AREB clade of bZIP transcription factors and ion channels. However, AHG1 differs from the other clade A PP2Cs in that it does not bind to the active sites of group 3 SnRK2s, interacts with only a subset of receptors, and requires high ABA concentrations to do so (Tischer et al. 2017; Krüger et al. 2025). In addition to this central de-repression mechanism for activating ABA response by phosphorylation of effectors, all signalosome components are also subject to post-translational modifications including phosphorylation and ubiquitination. Both of these modifications may be either activating or inhibitory, depending on the target protein residues and the specific kinases or E3 ligases involved (reviewed in Lim et al. 2022).

Activity and stability of the ABI5/ABF/ABRE clade of bZIP transcription factors is also regulated by a variety of post-translational modifications including phosphorylation, ubiquitination, sumoylation, and S-nitrosylation (reviewed in Yu et al. 2015). Multiple kinases, including SnRK2s, CBL-interacting protein kinase (CIPK)26, SOS2-like protein kinase 5 (PKS5), BRASSINOSTEROID INSENSITIVE(BIN)2, and MPK3, phosphorylate overlapping and discrete subsets of amino acid residues within ABI5 (Lyzenga et al. 2013; Zhou et al. 2015; Bhagat et al. 2025; Wei et al. 2022). Our immunoblots detected multiple forms of ABI5 in dry seeds and seeds germinating in the presence of ABA, but it is currently not clear which modifications are present on these ABI5 proteins.

The AFPs interact with multiple members of this clade of bZIPs (Garcia et al. 2008) and AFP1 was initially proposed to assist degradation of these bZIPs by promoting interactions with E3 ligases that ubiquitinate ABI5 (Lopez-Molina et al. 2003). Although these proposed E3 ligase interactions appear weak at most (Lynch et al. 2022), subsequent studies including the present one have expanded the list of AFP interactors to include proteins representing all elements of the ABA signalosome, some MPKs, several 14-3-3 proteins, the DELLA repressors of GA signaling (Finkelstein and Lynch 2022), the flowering regulator CONSTANS (Chang et al. 2019), WRKY36 and AHG1 in dormancy regulation (Deng et al. 2023; Krüger et al. 2025), several chromatin modifying factors (Pauwels et al. 2010; Lynch et al. 2017), and the protein kinase SALT OVERLY SENSITIVE(SOS)2 in regulating germination under salt stress (Wang et al. 2024). This extensive list of interactions is consistent with a role for AFPs as signaling hubs in a variety of processes, but raises the question of how specificity is achieved. Some of the likely options are regulated expression affecting availability of these proteins in different organs or stages of growth and changes in phosphorylation state or other post-translational modifications leading to changes in structure of the disordered regions.

### Kinases regulating ABA response target AFPs

The kinase families most highly associated with ABA or osmotic stress signaling are the SnRK2 kinases acting in the ABA core signalosome, SnRK3/CBL interacting kinases (CIPKs), MAP kinases, and calcium-dependent protein kinases (CDPKs). Many proteins can be phosphorylated at multiple sites, by multiple kinases, and the different sites may be phosphorylated sequentially with initial modifications altering the efficiency of subsequent events (reviewed in Miller and Turk 2018). For example, mammalian GSK3 kinases often require “priming” by phosphorylation at a residue 4 or 5 amino acids C-terminal to the target (Sutherland 2011). Furthermore, modifications at different sites may have opposing effects on activity of the substrate.

We focused on specific SnRK2s and MAP kinases that interacted with AFP1 and/or AFP2 in yeast 2-hybrid and/or BiFC assays, using in vitro kinase assays and co-expression to test for altered mobility on SDS-PAGE and Phos-tag gels. Mass spectrometric analysis identified Ser-112 of AFP2 as the sole residue phosphorylated by SnRK2.6, consistent with results reported for a phosphorylated site conserved in all 4 AFPs (Krüger et al. 2025). Mutagenesis converting this residue to either alanine or a phosphomimetic amino acid reduced, but did not eliminate, phosphorylation of the AFP2-AB domain. This indicated that SnRK2.6 could phosphorylate additional residues, albeit not efficiently enough to be detected by mass spectrometry. Mutagenesis converting an additional predicted SnRK2 target, Ser-85, to alanine eliminated phosphorylation of the AFP2-AB domain. In contrast, combining the S112A mutation with alanine substitution of Ser-259, the residue with the next highest match to the SnRK2 consensus target in the AFP2-BC domain, was not sufficient to eliminate phosphorylation of this domain in vitro, but did block ABA-induced changes in mobility of the YFP-AFP2-BC fusion in stable transgenic plants. Mutations affecting predicted MAP kinase target residues did not block ABA-induced changes in mobility of protein fusions, but severely reduced stability and consequently accumulation of these proteins. Surprisingly, transient expression of full-length AFP2 fusions containing alanine substitutions at multiple documented or predicted kinase targets produced even more distinct phosphorylated products than the wt fusion. These mutant fusion proteins in stable transgenics were barely detectable, but some were slightly stabilized by inhibiting proteasomal degradation.

### AFPs can form biomolecular condensates

Predictions of AFP structures show a series of likely protein binding regions separated by intrinsically disordered regions (Lynch et al. 2017) and the AFPs have been reported to interact with diverse proteins. The structure predicted by AlphaFold for the AFP2 monomer shows most of the predicted SnRK2 and MAPK phosphorylation sites in regions predicted to be disordered (Supplementary Figure S1). Only T319 is predicted with high confidence to be at the junction between beta-sheet and helical regions, while T76 and S112 are predicted to be near regions that might form helices. Our interaction data show that AFP2 forms dimers requiring regions in both the AB domain and the full-length protein (Supplementary Figure S7). When modeled as a dimer, the relatively structured regions of the B domain (aa 115-143) and the C domain (aa 281-326) of distinct chains are in proximity, and T76 is predicted to be in a fully unstructured region. The AB domain does not dimerize and is predicted to have all potential phosphorylation sites in disordered regions. Substrates are often bound to the kinase active site in an extended conformation (reviewed in Miller and Turk 2018), and these structural predictions are consistent with the observation that the AB domain is more accessible to SnRK2s than full-length AFP2.

Intrinsically disordered regions are recognized as drivers of biomolecular condensate formation, often through multivalent interactions, ie with multiple other molecules (Emenecker et al. 2021). Post-translational modifications such as phosphorylation alter the charge distribution and consequently the potential for interactions leading to condensate formation. Modeling possible effects of modification at the potential target residues tested in our mutant constructs showed limited impact of phosphorylation at either the 2 primary SnRK2 targets (S85 and S112) or the predicted MPK targets (S221 and T319), but additional phosphorylation of T76 or S259 was predicted to promote helix formation across much of the unstructured regions (Supplementary Figure S20). Further phosphorylation at additional target residues is predicted to give the AFP2 dimer even more structure, albeit with low confidence. In contrast, the ala substitutions tested in this study are predicted to maintain the dimer in a largely disordered state, such that additional residues might be accessible to additional kinases.

Initial descriptions of the AFPs showed a variety of localization patterns, depending on the experimental conditions. Transient overexpression of AFP(1)::CFP in either onion epidermal cells or Arabidopsis seedlings treated with ABA produced diffuse nuclear localization, but co-bombardment with 35S::ABI5 resulted in colocalization in nuclear bodies (Lopez-Molina et al. 2003). Stable expression of *AFP2pro:AFP2:GFP* in an *afp2* mutant background was also primarily nuclear, but excluded from nucleoli, in ABA- or NaCl-treated seedlings (Garcia et al. 2008). These conditions enhanced accumulation of both AFP2 and ABI5, but did not result in restriction to nuclear bodies, consistent with diffuse nuclear localization of AFPs likely to be in a phosphorylated state due to ABA or stress exposure. However, within 7 h of removal from these stress treatments AFP2:GFP was no longer visible in the nuclei, but still detected by immunoblot. In addition, some punctate fluorescence was visible in the cytoplasms of a few cells near the root meristem. Subsequent studies of interactions between AFPs and ABI5/ABF proteins by split YFP BiFC assays using transient overexpression in *N. benthamiana* also showed nuclear localization, but no foci (Lynch et al. 2017).

In the current BiFC assays, we found that co-expression of AFPs with kinases revealed interactions in nuclei, but excluded from nucleoli, whereas co-expression with PP2Cs resulted in formation of nuclear bodies over a broad range of sizes (Fig. 4), suggesting that dephosphorylated AFPs were more prone to form condensates. However, YFP-AFP2 co-expressed with cYFP-GRF4 also formed numerous small nuclear condensates, consistent with interactions dependent on phosphorylation. In an effort to determine which residues were most important for regulating condensate formation, we tested localization of YFP-AFP fusions with alanine substitutions at known or predicted phosphorylation sites. Surprisingly, the wt YFP-AFP2-AB domain fusion formed clusters of nuclear bodies, similar to the “grapebunch-like” condensates observed for ARF proteins (Powers et al. 2019), but the alanine-substituted mutant AFP2-AB domain fusions formed fewer clusters. This might reflect formation of complexes by phosphorylation-dependent interactions with 14-3-3 proteins that are reduced in the mutants. In contrast, the wt YFP-AFP2-BC fusion was mostly diffuse, but YFP-AFP2-BC fusions with mutations preventing phosphorylation of the predicted kinase target residues were more likely to form condensates, sometimes with small internal areas of exclusion (donut-shaped). When co-expressed with the tagged PP2C phosphatase Myc-AHG1, full-length AFP2 formed punctae whether or not the SnRK2 or GRF target residues could be phosphorylated. The AFP2-AB domain also continued to form punctae when co-expressed with AHG1, but fewer punctae formed when AHG1 and the AFP2-BC domain were co-expressed. In combination with the previously described diffuse nuclear localization in ABA-treated roots, this suggests that condensate formation is negatively correlated with ABA-induced phosphorylation.

### Physiological significance of AFP phosphorylation state

The AFPs have recently been reported to function as a downstream element of DOG1 control of dormancy. Proteomic and genetic studies showed that DOG1 maintains dormancy by repressing AHG1 and AHG3 activity (Krüger et al. 2025). In non-dormant or *dog1* mutant seeds, AHG1 dephosphorylates AFP2 at S112 and AFP1 at the corresponding conserved serine, allowing the AFPs to function as repressors of ABA response, resulting in germination. These results are consistent with our observation that ABA promotes a shift to the phosphorylated state, whereas co-expression with AHG1 has the opposite effect. Our studies used a gain of function approach, such that overexpression of the AFP domains and mutant forms leads to ABA resistance if the AFPs are physiologically active.

Functional tests of the physiological significance of these phosphorylation options focused on effects on ABA sensitivity of germination, cotyledon greening, storage protein accumulation, and seed longevity. We previously reported that overexpression of YFP-AFP2 could reduce ABA sensitivity nearly 100-fold and was so extreme in a wt background that homozygous transgenic lines failed to complete seed maturation and become desiccation tolerant (Lynch et al. 2017). Overexpression of the quadruple mutant (S85A, S112A, S221A, T319A), even though present at barely detectable levels, had a more extreme effect, also blocking maturation of heterozygous seeds. Homozygous YFP-AFP2 transgenes are tolerated in an *afp2* mutant background, where the total AFP2 concentration is lower, and YFP-AFP2-BC domain fusions are sufficient to reduce sensitivity only 30- to 50-fold, also resulting in viable seeds. The reduced impact of the truncated protein might reflect the loss of the EAR domain and therefore interactions with TPL/TPR co-repressors. However, these fusions could still inhibit ABI5 and related AREB/ABF clade transcription factors via interactions with their C domains (Garcia et al. 2008). Further truncation of AFP2, removing the B domain, results in much weaker reduction of ABA inhibited germination (Lynch et al. 2017). Surprisingly, removal of the unstructured region between the B and C domains to produce the “LITTLE NINJA” microprotein creates a dominant negative effect on jasmonic acid signaling resulting in stunted growth, but this construct was not tested for effects on ABA sensitivity (Hong et al. 2020).

Comparison of endogenous AFP and ABI5 levels over a range of ABA concentrations showed that ABI5 is usually in excess post-stratification, ranging from 8- to 10-fold higher than AFP1/2 when exposed to 1 µM ABA, but increasing to as much as 30-fold higher after 5 d post-stratification on media containing 10 µM ABA (Garcia et al. 2008). Extended incubation on the higher ABA concentrations led to accumulation of more AFP protein, effectively reducing the ABI5/AFP ratio, which was accompanied by increased germination. If this ratio is a sensor of physiological state that regulates germination, the severe disruption of the ratio by the AFP overexpression approach might be acting by titrating their interacting factors.

Mutations of the BC domain fusions converting only the SnRK2 targets, S112 or both S112 and S259, to alanine had minimal effect on ABA sensitivity of germination. However, the double mutant substantially reduced seed longevity. In contrast, mutants preventing phosphorylation of both predicted MPK target residues, S221 and T319, were less effective than the wt BC domain in conferring ABA resistance to concentrations higher than 10 µM ABA. This might reflect the reduced accumulation of this mutant form due at least in part to its reduced stability. Overexpression of phosphomimetic mutants did not confer greater ABA resistance than provided by the wt YFP-AFP2-BC. Although the mutants with the strongest effects on ABA sensitivity ((S112, S259)A and 3xD) had cruciferin levels similar to wt seeds when fresh, they showed accelerated loss of these storage proteins during shelf storage. Cruciferins have been found to serve as protectants from oxidative stress leading to seed deterioration during aging (Nguyen et al. 2015), but the 3xD mutants remained viable despite a massive loss of cruciferins within a year, suggesting that they rely on alternative protectants. A more detailed comparison of the proteomes or metabolomes of these mutants might identify components critical for seed longevity.

Although phosphomimetic substitutions are often used to simulate phosphorylated forms of proteins, they frequently do not confer the expected activities, especially if the phosphorylation is a binding site for an adaptor protein, eg 14-3-3 proteins (Dephoure et al. 2013; Kozeleková et al. 2022). Consequently, it is not surprising that the alanine and aspartic acid substitutions have some similar effects since both types of mutations prevent true phosphorylation. Given that AFP2 phosphorylation is required to interact with the 14-3-3 proteins, this might represent a sequestered state induced by ABA, maintaining ABI5 and other downstream signaling factors in an active state. As ABA levels decrease post-stratification, increased activity of the AHG PP2Cs could release the AFPs from complexes with the 14-3-3 proteins, permitting more inhibitory interactions with ABI5/ABFs/AREBs.

Finally, what is the physiological significance of these condensates? In other well-characterized plant systems, condensates include miRNA processing bodies (Xie et al. 2021), light response complexes regulating translation (Jang et al. 2019), high temperature-induced de-repression of gene expression (Bohn et al. 2024), sensors of hydration regulating germination (Dorone et al. 2021), and cytoplasmic sequestering of inactive transcription factors (Powers et al. 2019). Thus, they may represent complexes required for activity or inactivity of the relevant proteins. In the current work, all AFPs or AFP domains tested were present as overexpressed fusions to all or part of YFP. A trivial explanation is that at least some of these simply reflect high protein concentrations produced by overexpression in transient systems. However, some fusions formed foci despite accumulating to barely detectable levels, different mutations or co-expression conditions led to different localizations, and the BiFC studies permit visualization of only the fusion pairs being tested. As discussed above, the AFPs interact with many different proteins and are likely to be modified at additional sites by kinases not yet tested. Consequently, it is possible that these AFPs, and their variants created by distinct post-translational modifications, are present in diverse complexes with different functions, ie acting as signaling hubs.

## Materials and methods

### Plant materials and transgenes

Split YFP fusions for MPKs, GRFs, and PYLs were constructed using the Gateway-compatible pSITE-nEYFP-C1 (GenBank Acc. # GU734651) and pSITE-cEYFP-C1 (Acc. # GU734652) vectors and PCR products with attL ends added as described in Fu et al. (2008), following manufacturer's instructions for LR Clonase reactions (Invitrogen). The *35S::YFP:AFP2* fusion in pEarleyGate104 (Earley et al. 2006) and split YFP fusions for AFP1 (AT1G69260) and AFP2 (AT1G13740) were also constructed by LR Clonase recombinations, as described in Lynch et al. (2017). The split YFP fusions for SnRK2s and AHGs were described in Lynch et al. (2012). The *35S::YFP:AFP2* fusion lines in the wt (Col-0) and *afp2-1* (SALK_131676) (Alonso et al. 2003) mutant backgrounds were lines #4A2 and H1, respectively, reported in Lynch et al. (2017). All genes included in this study are listed in Supplementary Table S2. Mutations affecting potential phosphorylation sites were created by PCR using the high-fidelity polymerase ExTaq (TaKaRa Bio) and primers shown in Supplementary Table S3, designed using PrimerX (https://www.bioinformatics.org/primerx/documentation.html), with plasmids encoding cDNAs for full-length AFP2 or specific subdomains as templates. The AFP2-AB domain includes aa 1-149 and the AFP2-BC domain includes aa 94-348. For multi-site mutations, fragments overlapping at the mutation sites were amplified, then annealed, and amplified by attL primers at the end points to create Gateway-compatible fragments.

Arabidopsis plants were grown in pots in growth chambers under continuous light at 22 °C. *Agrobacterium tumefaciens*-mediated direct transformation of constructs with mutations in potentially phosphorylated residues was performed by the floral dip method (Clough and Bent 1998), followed by selection of BASTA-resistant seedlings on germination medium (GM: 0.5× MS salts and vitamins, 1% sucrose) supplemented with 8 µg/mL BASTA (“Finale”, AgrEvo Environmental Health). Homozygous lines were identified by production of 100% BASTA-resistant progeny. Following crosses between *35S:YFP:AFP2-BC* fusion lines and *afp2-1* mutants, YFP-AFP2-BC overexpressing progeny were selected by BASTA resistance and homozygous *afp2-1* segregants were identified by PCR-based genotyping, as described at http://signal.salk.edu/tdnaprimers.2.html.

### Yeast 2-hybrid constructs and assays

Fusions between the GAL4 activation domain (AD) and full-length PP2C cDNAs were described in Lynch et al. (2012). Fusions between the AD domain and full-length and partial AFP cDNAs were described in Lynch et al. (2017). Fusions between the AD domain and 14-3-3 cDNAs were constructed by CRE-lox recombination between pUNI clones and the pACT2-lox vector (Liu et al. 1998). Fusions between the GAL4 DNA BD and either AFP or PYL cDNAs were constructed using the pGBKT7-DEST vector (Lu et al. 2010) and PCR products with attL ends. BD fusions with SnRK2.2, 2.3 and MPK6 were constructed by CRE-lox recombination using the pAS2lox vector and by LR Clonase reactions with the pGBKT7-DEST vector and pDONR clones for SnRK2.6, SnRK2.10, and MPK3.

BD fusions were transformed into yeast line PJ69-4A selecting for growth on yeast synthetic medium (YSM) without trp. AD fusions were transformed into Y187, selecting for growth on YSM without leu. Interactions were tested by matings between pairs of lines carrying BD and AD fusions; following overnight incubation on YPD plates, matings were replica plated to YSM lacking leu and trp to select for diploids or YSM also missing histidine (his) and, in some cases, adenine to screen for diploids that had activated the HIS3 and ADE2 reporter genes. Diploids carrying BD-PYLs and AD vector or AD-AFPs were grown in YSM lacking leu and trp, then diluted to an OD600 of 0.9, before making serial 10-fold dilutions that were spotted in replicates on YSM lacking leu and trp, or also lacking his. Diploids combining BD-AFPs with AD vector or AD-GRFs were also analyzed by growth assays with serially diluted cultures.

### Plant growth conditions

Germination assays testing ABA sensitivity of age-matched seeds were performed on minimal nutrient media supplemented with ABA at concentrations over the range from 0 to 50 µM, as described in Lynch et al. (2017). Accumulation of fusion proteins was assayed by immunoblots of seeds or seedlings harvested after 5 d incubation on GM (0.5× MS salts and vitamins, 1% sucrose) or minimal media solidified with 0.7% agar, then subjected to ABA at the indicated concentrations, or 100 µM cycloheximide (CHX) (Acros Organics, #357420010) or MG132 (UBPBio, # F1101) for the indicated times.

For transient expression, *Agrobacterium* lines expressing fusion proteins were combined with GV3101 expressing the P19 protein of tomato bushy stunt virus to enhance transient expression, as described in Voinnet et al. (2003). Cultures of all bacteria to be used in infiltration were grown overnight in YEP media with appropriate selective antibiotics, diluted to an OD_600_ of 1.0 in 10 mM MgCl_2_, 10 mM MES pH 5.6, and 0.2 mM acetosyringone (Aldrich #D134406) and rocked at room temperature for 2 to 3 h prior to mixing and infiltration of leaves on 2 to 3-wk-old *N. benthamiana* plants. For BiFC assays, Agrobacteria expressing cYFP and nYFP fusions were combined just prior to infiltration. Fluorescence was scored 2 to 4 d later, using an Olympus AX70 microscope or a Leica SP8 Resonant Scanning Confocal microscope. Confocal images were processed with Fiji (ImageJ) to produce maximum intensity Z-stack composites.

For stability assays of transiently transformed plants, infiltrated leaves were further infiltrated at 3 d post-Agrobacterium infiltration with GM supplemented with 100 µM cycloheximide (CHX) or MG132, or a solvent control and incubated for 6 h prior to harvest.

### Immunoblots

Arabidopsis seedlings or *N. benthamiana* leaf tissue were ground directly in 2× Laemmli loading buffer (1.5 to 2 µL/mg FW), microfuged 10 min at 4 °C to pellet debris, then the supernatants were boiled 5 min prior to fractionation by SDS-PAGE or SuperSep Phos-tag PAGE (Fujifilm Wako Chemicals). Proteins were transferred to nitrocellulose filters (Prometheus, Genesee Scientific, San Diego, CA) and stained with Ponceau S, as described in Lynch et al. (2017). Filters were blocked with Casein blocking buffer (LI-COR Biosciences, Lincoln, NE), then co-incubated with anti-GFP mAb (1:10000, UBPBio, Aurora, CO) and/or anti-actin mAb (A0480, Sigma) primary antibodies, followed by anti-mouse secondary IRDye 800 conjugated IgGs (LiCor, #926-32210), and visualized using the 800 channel of the Licor Odyssey Infrared Imaging System. Mature dry Arabidopsis seeds were ground in 20 µL/mg FW 1× Laemmli loading buffer, then processed as above. ABI5 was detected with anti-ABI5pAb (1:10000, Ab98831, Abcam) primary antibodies, followed by anti-rabbit secondary IRDye 700 conjugated IgGs (LiCor #926-68071), and visualized using the 700 channel of the Licor Odyssey Infrared Imaging System.

### Recombinant protein purification

Fusions between His6-tags and full-length, partial, and mutant AFP coding sequences were constructed by CRE-lox recombination between pUNI cDNAs and the pHB3-His6 vector, as described in http://signal.salk.edu/UPS_Protocols_Vectors.pdf. Primers used for subcloning and mutagenesis are shown in Supplementary Table S3. Plasmids carrying fusion genes were transformed into *E.coli* BL21C+ and expression was induced by growth overnight at 19 to 20 °C in Overnight Express Instant TB medium (Novagen). Cells were harvested by microcentrifugation for 3 min at 16 K rpm and resuspended in 140 µL Lysis Buffer (8.3 M urea, 0.1 M NaPhosphate pH 8) per mL of initial culture. His6 fusions were affinity purified in batch mode with HIS-select Nickel Affinity beads (Millipore P6611) according to the manufacturer's protocol for denaturing conditions, eluting with 8 M urea, 0.1 M NaPhosphate pH 5.5. The urea concentration was reduced 10-fold by dilution into kinase reaction buffer.

GST fusions to full-length SnRK2.6 also made use of the pUNI vector system, and induction overnight in TB Express, but proteins were extracted under native conditions with CelLytic B in 1× PBS, 10 mM DTT, 83 units/mL Benzonase nuclease, 1 mM PMSF, and 5 mg/mL lysozyme, followed by purification on glutathione-agarose beads (UBP-Bio, P3060-10) and elution with 30 mM Tris pH9, 300 mM NaCl, 5 mM DTT, 0.5% Triton-X100, 1 mM PMSF, and 40 mM glutathione.

### In vitro kinase assays

Purified GST-SnRK2.6 was activated by 1 h pre-incubation in kinase buffer (25 mM Tris pH 7.5, 12 mM MgCl_2_, 2 mM DTT) with 1 mM ATP, then combined with His-AFP fusions maintaining the buffer and ATP concentrations for 1 additional hour of incubation. Phosphorylation was monitored in wt and mutant constructs by incorporation of ^32^P from gamma-labeled ATP (0.1 to 0.2 µCi/rxn) during the latter incubation. SDS sample buffer was added to 1× final, samples were boiled 5 min, then fractionated on 13% SDS-PAGE, dried, and autoradiographed. Following assays with only cold ATP, gel slices containing lower mobility products were excised and sent to UC Davis for mass spec analysis. Additional SnRK assays with mutant AFP fusion proteins were analyzed by ProQ Diamond Phosphoprotein staining according to manufacturer's instructions (Invitrogen).

### Mass spectrometry

Coomassie stained gel slices were processed with a standard washing, reduction/alkylation followed by tryptic digestion, as described in https://www.promega.com/-/media/files/resources/protocols/technical-bulletins/101/proteasemax-surfactant-trypsin-enhancer.pdf. The mass spectrometer used was a Xevo G2 QTof coupled to a nanoAcquity UPLC system (Waters, Milford, MA). Samples were loaded onto a C18 Waters Trizaic nanotile of 85 µm × 100 mm, 1.7 µm (Waters, Milford, MA). The column temperature was set to 45 °C with a flow rate of 0.45 mL/min. The mobile phase consisted of A (water containing 0.1% formic acid) and B (acetonitrile containing 0.1% formic acid). A linear gradient elution program was used: 0 to 40 min, 3 to 40% (B); 40 to 42 min, 40 to 85% (B); 42 to 46 min, 85% (B); 46 to 48 min, 85 to 3% (B); and 48 to 60 min, 3% (B).

Mass spectrometry data were recorded for 60 min for each run and controlled by MassLynx 4.2 SCN990 (Waters, Milford, MA). Acquisition mode was set to positive polarity under resolution mode. Mass range was set from 50 to 2,000Da. Capillary voltage was 3.5 kV, sampling cone at 25 V, and extraction cone at 2.5 V. Source temperature was held at 110 °C. Cone gas was set to 25 L/h, nano flow gas at 0.10 Bar, and desolvation gas at 1,200 L/h. Leucine–enkephalin at 720 pmol/µL (Waters, Milford, MA) was used as the lock mass ion at m/z 556.2771 and introduced at 1 µL/min at 45 second intervals with a 3 scan average and mass window of ±0.5 Da. The MSe data were acquired using 2 scan functions corresponding to low energy for Function 1 and high energy for Function 2. Function 1 had collision energy at 6 V, and Function 2 had a collision energy ramp of 18–42 V.

Raw data were searched using Protein Lynx Global Server 3.0.3 Expression 2.0 (Waters, Milford, Massachusetts). Processing parameters consisted of a low energy threshold set at 200.0 counts, an elevated energy threshold set at 25.0 counts, and an intensity threshold set at 1,500 counts. A dedicated database was constructed containing common housekeeping proteins as well as the provided sequence of the target protein. The database was randomized within PLGS. Possible structure modifications included for consideration were methionine oxidation, carbamidomethylation of cysteine, deamidation of asparagine or glutamine, dehydration of serine or threonine, and phosphorylation of serine, threonine, or tyrosine.

### RT-qPCR analysis

RNA was isolated from *N. benthamiana* leaf tissue or Arabidopsis seedlings as described in Lynch et al. (2017). DNA-free total RNA was prepared by incubation with RQ1 DNase (Promega) and RNAsin for 15 min at room temperature, followed by inactivation of the DNase by addition of EGTA and incubation for 5 min at 65 °C, then clean-up over Qiagen RNeasy or Zymo-Clean columns according to manufacturers' instructions. Approximately 0.5 µg of RNA was used as template for cDNA synthesis by MMLV reverse transcriptase (Promega), using a 10:1 mix of random hexamers and oligo dT as primers. cDNA concentrations were checked by RT-qPCR, using Forget-Me-Not master mix (Biotium) in an iQ5 or CFX cycler (Bio-Rad), according to manufacturer's instructions. Primers used for normalizing were selected for uniform expression in Arabidopsis seeds or young seedlings subjected to a variety of treatments: AT5G46630 and At1g13320 (Czechowski et al. 2005) or a validated reference gene for *N. benthamiana* (Pombo et al. 2019). Equal amounts of cDNA were used as templates for reactions with all other primer sets (listed in Supplementary Table S3), quantified relative to a standard curve spanning the range of concentrations present in all genotypes of transgene expression.

### Accession numbers

Sequence data from this article can be found in the GenBank/EMBL data libraries under accession numbers listed in Supplementary Table S2.

## Supplementary Material

kiaf674_Supplementary_Data

## Data Availability

The data underlying this article are available in the article and in its online supplementary material. Mass spectrometry data is available as linked in the supplementary material.

## Supplementary material

The following materials are available in the online version of this article.

**Supplementary Figure S1.** Alphafold prediction of AFP2 structure (Model AF-Q9LMX5-F1) with locations of predicted SnRK2 or MPK phosphorylation sites labeled.

**Supplementary Figure S2.** Interactions between MPK6, AFPs and their subdomains detected by yeast two-hybrid assays.

**Supplementary Figure S3.** Expression of MPKs in dry seeds and either 24 h post-2d-stratification or after 24h imbibition in water or 3 •M ABA following 2-4 months after-ripening.

**Supplementary Figure S4.** Yeast two-hybrid test of interactions between ABA receptors and AFPs, presented as GAL4BD-PYR/PYL and GAL4AD-AFP fusions.

**Supplementary Figure S5.** Expression of PYLs, GRFs and AFPs in maturing embryos, dry seeds and either 24 h post-2d-stratification, or after 24h imbibition in water following 2-4 months after-ripening.

**Supplementary Figure S6.** Interactions between 14-3-3 proteins (GRFs) and ABA core signaling elements.

**Supplementary Figure S7.** Potential dimerization domains of AFP2

**Supplementary Figure S8.** Biomolecular fluorescence complementation of full length AFP2 and AFP1 and subdomains versus ABA core signaling components in N. benthamiana leaves.

**Supplementary Figure S9.** Predicted phosphorylated residues and disordered regions in AFP1 and AFP2.

**Supplementary Figure S10.** Mass spectrometric analysis of phosphorylation site(s) by in vitro kinase reaction of His-AFP2-AB incubated with GST-SnRK2.6.

**Supplementary Figure S11.** Tests of potential phosphorylation sites by kinase reactions with mutant AFP substrates.

**Supplementary Figure S12.** Comparison of phosphorylation status of AFP2-BC domain and full-length AFP2 by Phos-tag gels.

**Supplementary Figure S13.** Effects of ABA, mutations and genetic background on expression and mobility of YFP-AFP2-BC transgenes in stably transformed A. thaliana.

**Supplementary Figure S14.** Comparison of protein and transcript levels for AFP2-AB or AFP2-BC domain fusions.

**Supplementary Figure S15.** Comparison of stability and accumulation of transiently expressed wild type, double (S112A,S259A or S221A,T319A) or triple (3xA=S112A,S221A,T319A) mutant AFP2-BC domain fusions to YFP.

**Supplementary Figure S16.** Germination phenotypes of wild type (Col) and transgenic seeds expressing the indicated YFP-AFP2 fusions after 7d incubation on either 10 or 50 µM ABA.

**Supplementary Figure S17.** Transgenic line hemizygous for 35S-YFP-AFP2(S85,S112,S221,T319)A fails to complete seed maturation.

**Supplementary Figure S18.** Effects of phosphomutant or phosphomimetic YFP-AFP2 fusions on storage protein accumulation and seed longevity.

**Supplementary Figure S19.** Effect of ABA on accumulation and phosphorylation of ABI5 in seeds with wild type or mutant YFP-AFP2-BC fusions in an afp2 mutant background.

**Supplementary Figure S20.** Alphafold3 predictions of AFP2 dimer structure when phosphorylated, or blocked from phosphorylation, at residues tested in this work (Abramson et al., 2024).

**Supplementary Table S1.** AFP2 predicted phosphorylation sites

**Supplementary Table S2.** List of genes included in this study.

**Supplementary Table S3.** Primers used in this study.

## Dive Curated Terms

The following phenotypic, genotypic, and functional terms are of significance to the work described in this paper:

AHG1 Gramene: AT5G51760
AHG1 Araport: AT5G51760

AFP3 Gramene: AT3G29575
AFP3 Araport: AT3G29575

COP1 Gramene: AT2G32950
COP1 Araport: AT2G32950

GRF1 Gramene: At2g22840
GRF1 Araport: At2g22840

GRF2 Gramene: AT1G78300
GRF2 Araport: AT1G78300

WRKY36 Gramene: AT1G69810
WRKY36 Araport: AT1G69810

ABI1 Gramene: AT4G26080
ABI1 Araport: AT4G26080

AFP2 Gramene: AT1G13740
AFP2 Araport: AT1G13740

GRF7 Gramene: AT3G02520
GRF7 Araport: AT3G02520

ABI3 Gramene: AT3G24650
ABI3 Araport: AT3G24650

ABI2 Gramene: AT5G57050
ABI2 Araport: AT5G57050

MPK7 Gramene: At2g18170
MPK7 Araport: At2g18170

PKS5 Gramene: AT2G30360
PKS5 Araport: AT2G30360

PYL2 Gramene: AT2G26040
PYL2 Araport: AT2G26040

ABI5 Gramene: AT2G36270
ABI5 Araport: AT2G36270

AFP1 Gramene: AT3G02520
AFP1 Araport: AT3G02520

AFP4 Gramene: AT3G02140
AFP4 Araport: AT3G02140

AHG3 Gramene: AT3G11410
AHG3 Araport: AT3G11410

GRF3 Gramene: AT5G38480
GRF3 Araport: AT5G38480

GRF4 Gramene: AT1G35160
GRF4 Araport: AT1G35160

HAI1 Gramene: AT5G59220
HAI1 Araport: AT5G59220

HAI2 Gramene: AT1G07430
HAI2 Araport: AT1G07430

HAB1 Gramene: AT1G72770
HAB1 Araport: AT1G72770

MPK3 Gramene: AT3G45640
MPK3 Araport: AT3G45640

MPK4 Gramene: AT4G01370
MPK4 Araport: AT4G01370

MPK6 Gramene: MD01G1069100
MPK6 Araport: MD01G1069100

MPK16 Gramene: AT5G19010
MPK16 Araport: AT5G19010

MPK17 Gramene: AT2G01450
MPK17 Araport: AT2G01450

MPK20 Gramene: AT2G42880
MPK20 Araport: AT2G42880

SnRK2.2 Gramene: AT3G50500
SnRK2.2 Araport: AT3G50500

SnRK2.3 Gramene: AT5G66880
SnRK2.3 Araport: AT5G66880

SnRK2.6 Gramene: AT4G33950
SnRK2.6 Araport: AT4G33950

SnRK2.10 Gramene: AT1G60940
SnRK2.10 Araport: AT1G60940

PYR1 Gramene: AT4G17870
PYR1 Araport: AT4G17870

PYL1 Gramene: AT5G46790
PYL1 Araport: AT5G46790

PYL3 Gramene: AT1G73000
PYL3 Araport: AT1G73000

PYL4 Gramene: AT2G38310
PYL4 Araport: AT2G38310

PYL5 Gramene: AT5G05440
PYL5 Araport: AT5G05440

PYL6 Gramene: AT2G40330
PYL6 Araport: AT2G40330

PYL7 Gramene: AT4G01026
PYL7 Araport: AT4G01026

PYL8 Gramene: AT5G53160
PYL8 Araport: AT5G53160

PYL9 Gramene: AT1G01360
PYL9 Araport: AT1G01360

PYL10 Gramene: AT4G27920
PYL10 Araport: AT4G27920

PYL11 Gramene: AT5G45860
PYL11 Araport: AT5G45860

PYL12 Gramene: AT5G45870
PYL12 Araport: AT5G45870

PYL13 Gramene: AT4G18620
PYL13 Araport: AT4G18620

## References

[kiaf674-B1] Ali A, Pardo JM, Yun D-J. Desensitization of ABA-signaling: the swing from activation to degradation. Front Plant Sci. 2020:11:379. 10.3389/fpls.2020.00379. 32391026 PMC7188955

[kiaf674-B2] Alonso JM et al Genome-wide insertional mutagenesis of Arabidopsis thaliana. Science. 2003:301:653–657. 10.1126/science.1086391. 12893945

[kiaf674-B3] Bhagat PK, Verma N, Pandey S, Verma D, Sinha AK. MPK3 mediated phosphorylation inhibits the dimerization of ABI5 to fine-tune the ABA signaling in Arabidopsis. Plant Physiol Biochem. 2025:221:109690. 10.1016/j.plaphy.2025.109690. 40010200

[kiaf674-B4] Bohn L et al The temperature sensor TWA1 is required for thermotolerance in Arabidopsis. Nature. 2024:629:1126–1132. 10.1038/s41586-024-07424-x. 38750356 PMC11136664

[kiaf674-B5] Camoni L, Visconti S, Aducci P, Marra M. 14-3-3 proteins in plant hormone signaling: doing several things at once. Front Plant Sci. 2018:9:297. 10.3389/fpls.2018.00297. 29593761 PMC5859350

[kiaf674-B6] Chang G et al ABI5-BINDING PROTEIN2 coordinates CONSTANS to delay flowering by recruiting the transcriptional corepressor TPR2. Plant Physiol. 2019:179:477–490. 10.1104/pp.18.00865. 30514725 PMC6426417

[kiaf674-B7] Chen K et al Abscisic acid dynamics, signaling, and functions in plants. J Integr Plant Biol. 2020:62:25–54. 10.1111/jipb.12899. 31850654

[kiaf674-B8] Chen X et al The MKK3-MPK7 cascade phosphorylates ERF4 and promotes its rapid degradation to release seed dormancy in Arabidopsis. Mol Plant. 2023:16:1743–1758. 10.1016/j.molp.2023.09.006. 37710960

[kiaf674-B9] Clough S, Bent A. Floral dip: a simplified method for *Agrobacterium*-mediated transformation of *Arabidopsis thaliana*. Plant J. 1998:16:735–743. 10.1046/j.1365-313x.1998.00343.x. 10069079

[kiaf674-B10] Cutler SR, Rodriguez PL, Finkelstein RR, Abrams SR. Abscisic acid: emergence of a core signaling network. Ann Rev of Plant Biology. 2010:61:651–679. 10.1146/annurev-arplant-042809-112122. 20192755

[kiaf674-B11] Czechowski T, Stitt M, Altmann T, Udvardi MK, Scheible W-RD. Genome-wide identification and testing of superior reference genes for transcript normalization in Arabidopsis. Plant Physiol. 2005:139:5–17. 10.1104/pp.105.063743. 16166256 PMC1203353

[kiaf674-B12] Danquah A et al Identification and characterization of an ABA-activated MAP kinase cascade in Arabidopsis thaliana. Plant J. 2015:82:232–244. 10.1111/tpj.12808. 25720833

[kiaf674-B13] Deng G et al A transcription factor WRKY36 interacts with AFP2 to break primary seed dormancy by progressively silencing DOG1 in Arabidopsis. New Phytol. 2023:238:688–704. 10.1111/nph.18750. 36653950

[kiaf674-B14] Dephoure N, Gould KL, Gygi SP, Kellogg DR. Mapping and analysis of phosphorylation sites: a quick guide for cell biologists. Mol Biol Cell. 2013:24:535–542. 10.1091/mbc.e12-09-0677. 23447708 PMC3583658

[kiaf674-B15] De Zelicourt A, Colcombet J, Hirt H. The role of MAPK modules and ABA during abiotic stress signaling. Trends Plant Sci. 2016:21:677–685. 10.1016/j.tplants.2016.04.004. 27143288

[kiaf674-B16] Dorone Y et al A prion-like protein regulator of seed germination undergoes hydration-dependent phase separation. Cell. 2021:184:4284–4298.e27. 10.1016/j.cell.2021.06.009. 34233164 PMC8513799

[kiaf674-B17] Earley K et al Gateway-compatible vectors for plant functional genomics and proteomics. Plant J. 2006:45:616–629. 10.1111/j.1365-313X.2005.02617.x. 16441352

[kiaf674-B18] Eisner N et al Phosphorylation of serine 114 of the transcription factor ABSCISIC ACID INSENSITIVE 4 is essential for activity. Plant Sci. 2021:305:110847. 10.1016/j.plantsci.2021.110847. 33691973

[kiaf674-B19] Emenecker RJ, Holehouse AS, Strader LC. Biological phase separation and biomolecular condensates in plants. Annu Rev Plant Biol. 2021:72:17–46. 10.1146/annurev-arplant-081720-015238. 33684296 PMC8221409

[kiaf674-B20] Erickson McNally BJ-A . Expression patterns of legume-specific cell wall proteins and their genomic organization in Medicago truncatula and investigating the relationship between post-translational modification and AFP2 function [PhD thesis]. University of California.

[kiaf674-B21] Finkelstein R . Abscisic acid synthesis and response. Arabidopsis Book. 2013:11:e0166. 10.1199/tab.0166. 24273463 PMC3833200

[kiaf674-B22] Finkelstein RR, Lynch TJ. Overexpression of ABI5 binding proteins suppresses inhibition of germination due to overaccumulation of DELLA proteins. Int J Mol Sci. 2022:23:5537. 10.3390/ijms23105537. 35628355 PMC9144539

[kiaf674-B23] Fu C, Wehr DR, Edwards J, Hauge B. Rapid one-step recombinational cloning. Nucl Acids Res. 2008:36. 10.1093/nar/gkn167. PMC2396420 18424799

[kiaf674-B24] Fujii H, Zhu J-K. Arabidopsis mutant deficient in 3 abscisic acid-activated protein kinases reveals critical roles in growth, reproduction, and stress. Proc Natl Acad Sci U S A. 2009:106:8380–8385. 10.1073/pnas.0903144106. 19420218 PMC2688869

[kiaf674-B25] Fujita Y et al Three SnRK2 protein kinases are the main positive regulators of abscisic acid signaling in response to water stress in Arabidopsis. Plant Cell Physiol. 2009:50:2123–2132. 10.1093/pcp/pcp147. 19880399

[kiaf674-B26] Galletti R, Ferrari S, De Lorenzo G. Arabidopsis MPK3 and MPK6 play different roles in basal and oligogalacturonide- or flagellin-induced resistance against Botrytis cinerea. Plant Physiol. 2011:157:804–814. 10.1104/pp.111.174003. 21803860 PMC3192574

[kiaf674-B27] Garcia M, Lynch T, Peeters J, Snowden C, Finkelstein R. A small plant-specific protein family of ABI five binding proteins (AFPs) regulates stress response in germinating *Arabidopsis* seeds and seedlings. Plant Mol Biol. 2008:67:643–658. 10.1007/s11103-008-9344-2. 18484180

[kiaf674-B28] Group M et al Mitogen-activated protein kinase cascades in plants: a new nomenclature. Trends Plant Sci. 2002:7:301–308. 10.1016/S1360-1385(02)02302-6. 12119167

[kiaf674-B29] Guan Y, Lu J, Xu J, Mcclure B, Zhang S. Two mitogen-activated protein kinases, MPK3 and MPK6, are required for funicular guidance of pollen tubes in Arabidopsis. Plant Physiol. 2014:165:528–533. 10.1104/pp.113.231274. 24717717 PMC4044831

[kiaf674-B30] Guo H et al Plastid- nucleus communication involves calcium-modulated MAPK signalling. Nat Commun. 2016:7:12173. 10.1038/ncomms12173. 27399341 PMC4942575

[kiaf674-B31] Hong S-Y et al Heterologous microProtein expression identifies LITTLE NINJA, a dominant regulator of jasmonic acid signaling. Proc Natl Acad Sci U S A. 2020:117:26197–26205. 10.1073/pnas.2005198117. 33033229 PMC7584889

[kiaf674-B32] Jagodzik P, Tajdel-Zielinska M, Ciesla A, Marczak M, Ludwikow A. Mitogen-activated protein kinase cascades in plant hormone signaling. Front Plant Sci. 2018:9:1387. 10.3389/fpls.2018.01387. 30349547 PMC6187979

[kiaf674-B33] Jang G-J, Yang J-Y, Hsieh H-L, Wu S-H. Processing bodies control the selective translation for optimal development of Arabidopsis young seedlings. Proc Natl Acad Sci U S A. 2019:116:6451–6456. 10.1073/pnas.1900084116. 30850529 PMC6442596

[kiaf674-B34] Jaspert N, Throm C, Oecking C. Arabidopsis 14-3-3 proteins: fascinating and less fascinating aspects. Front Plant Sci. 2011:2:96. 10.3389/fpls.2011.00096. 22639620 PMC3355631

[kiaf674-B35] Ko DK, Brandizzi F. Network-based approaches for understanding gene regulation and function in plants. Plant J. 2020:104:302–317. 10.1111/tpj.14940. 32717108 PMC8922287

[kiaf674-B36] Kobayashi Y, Yamamoto S, Minami H, Kagaya Y, Hattori T. Differential activation of the rice sucrose nonfermenting1-related protein kinase2 family by hyperosmotic stress and abscisic acid. Plant Cell. 2004:16:1163–1177. 10.1105/tpc.019943. 15084714 PMC423207

[kiaf674-B37] Koornneef M, Reuling G, Karssen C. The isolation and characterization of abscisic acid-insensitive mutants of *Arabidopsis thaliana*. Physiol Plant. 1984:61:377–383. 10.1111/j.1399-3054.1984.tb06343.x.

[kiaf674-B38] Kozeleková A et al Phosphorylated and phosphomimicking variants may differ—A case study of 14-3-3 protein. Front Chem. 2022:10:835733. 10.3389/fchem.2022.835733 35321476 PMC8935074

[kiaf674-B39] Krüger T et al DOG1 controls dormancy independently of ABA core signaling kinases regulation by preventing AFP dephosphorylation through AHG1. Sci Adv. 2025:11:eadr8502. 10.1126/sciadv.adr8502. 40020062 PMC11870083

[kiaf674-B40] Kulik A, Wawer I, Krzywińska E, Bucholc M, Dobrowolska G. SnRK2 protein kinases–key regulators of plant response to abiotic stresses. OMICS. 2011:15:859–872. 10.1089/omi.2011.0091. 22136638 PMC3241737

[kiaf674-B41] Lee S-J, Lee MH, Kim J-I, Kim SY. Arabidopsis putative MAP kinase kinase kinases Raf10 and Raf11 are positive regulators of seed dormancy and ABA response. Plant Cell Physiol. 2014:56:84–97. 10.1093/pcp/pcu148. 25324504

[kiaf674-B42] Lim J, Lim CW, Lee SC. Core components of abscisic acid signaling and their post-translational modification. Front Plant Sci. 2022:13:895698. 10.3389/fpls.2022.895698. 35712559 PMC9195418

[kiaf674-B43] Liu Q, Li MZ, Leibham D, Cortez D, Elledge SJ. The univector plasmid-fusion system, a method for rapid construction of recombinant DNA without restriction enzymes. Curr Biol. 1998:8:1300–1309. 10.1016/S0960-9822(07)00560-X. 9843682

[kiaf674-B44] Liu Q, Liu W, Niu Y, Wang T, Dong J. Liquid–liquid phase separation in plants: advances and perspectives from model species to crops. Plant Commun. 2023:5:100663. 10.1016/j.xplc.2023.100663. 37496271 PMC10811348

[kiaf674-B45] Lopez-Molina L, Mongrand S, Kinoshita N, Chua N-H. AFP is a novel negative regulator of ABA signaling that promotes ABI5 protein degradation. Genes Dev. 2003:17:410–418. 10.1101/gad.1055803. 12569131 PMC195991

[kiaf674-B46] Lu Q, Tang X, Tian G, Wang F, Liu K, Nguyen V, Kohalmi SE, Keller WA, Tsang EWT, Harada JJ, et al Arabidopsis homolog of the yeast T REX-2 mRNA export complex: components and anchoring nucleoporin. Plant J. 2010:61:259–270. 10.1111/j.1365-313X.2009.04048.x. 19843313

[kiaf674-B47] Lumba S et al A mesoscale abscisic acid hormone interactome reveals a dynamic signaling landscape in Arabidopsis. Dev Cell. 2014:29:360–372. 10.1016/j.devcel.2014.04.004. 24823379

[kiaf674-B48] Lynch T et al ABI5 binding protein2 inhibits ABA responses during germination without ABA-INSENSITIVE5 degradation. Plant Physiol. 2022:189:666–678. 10.1093/plphys/kiac096. 35258597 PMC9157056

[kiaf674-B49] Lynch T, Erickson B, Finkelstein R. Direct interactions of ABA-insensitive (ABI)-clade protein phosphatase (PP)2Cs with calcium-dependent protein kinases and ABA response element- binding bZIPs may contribute to turning off ABA response. Plant Mol Biol. 2012:80:647–658. 10.1007/s11103-012-9973-3. 23007729

[kiaf674-B50] Lynch TJ, Erickson BJ, Miller DR, Finkelstein RR. ABI5-binding proteins (AFPs) alter transcription of ABA-induced genes via a variety of interactions with chromatin modifiers. Plant Mol Biol. 2017:93:403–418. 10.1007/s11103-016-0569-1. 27942958

[kiaf674-B51] Lyzenga WJ, Liu H, Schofield A, Muise-Hennessey A, Stone SL. Arabidopsis CIPK26 interacts with KEG, components of the ABA signalling network and is degraded by the ubiquitin-proteasome system. J Exp Bot. 2013:64:2779–2791. 10.1093/jxb/ert123. 23658427 PMC3697954

[kiaf674-B52] Madeira F et al 14-3-3-Pred: improved methods to predict 14-3-3-binding phosphopeptides. Bioinformatics. 2015:31:2276–2283. 10.1093/bioinformatics/btv133. 25735772 PMC4495292

[kiaf674-B53] Maszkowska J et al The multifaceted regulation of SnRK2 kinases. Cells. 2021:10:2180. 10.3390/cells10092180. 34571829 PMC8465348

[kiaf674-B54] Maymon T, Eisner N, Bar-Zvi D. The ABCISIC ACID INSENSITIVE (ABI) 4 transcription factor is stabilized by stress, ABA and phosphorylation. Plants (Basel). 2022:11(16):2179. 10.3390/plants11162179. 36015481 PMC9414092

[kiaf674-B55] Miller CJ, Turk BE. Homing in: mechanisms of substrate targeting by protein kinases. Trends Biochem Sci. 2018:43(5):380–394. 10.1016/j.tibs.2018.02.009. 29544874 PMC5923429

[kiaf674-B56] Nakashima K et al Three Arabidopsis SnRK2 protein kinases, SRK2D/SnRK2.2, SRK2E/SnRK2.6/OST1 and SRK2I/SnRK2.3, involved in ABA signaling are essential for the control of seed development and dormancy. Plant Cell Physiol. 2009:50:1345–1363. 10.1093/pcp/pcp083. 19541597

[kiaf674-B57] Née G, Krüger T. Dry side of the core: a meta-analysis addressing the original nature of the ABA signalosome at the onset of seed imbibition. Front Plant Sci. 2023:14:1192652. 10.3389/fpls.2023.1192652. 37476171 PMC10354442

[kiaf674-B58] Nguyen T-P, Cueff G, Hegedus DD, Rajjou L, Bentsink L. A role for seed storage proteins in Arabidopsis seed longevity. J Exp Bot. 2015:66:6399–6413. 10.1093/jxb/erv348. 26184996 PMC4588887

[kiaf674-B59] Nishimura N et al *ABA-Hypersensitive Germination1* encodes a protein phosphatase 2C, an essential component of abscisic acid signaling in Arabidopsis seed. Plant J. 2007:50:935–949. 10.1111/j.1365-313X.2007.03107.x. 17461784

[kiaf674-B60] Park S-Y et al Abscisic acid inhibits type 2C protein phosphatases via the PYR/PYL family of START proteins. Science. 2009:324:1068–1071. 10.1126/science.1173041. 19407142 PMC2827199

[kiaf674-B61] Pauwels L et al NINJA connects the co-repressor TOPLESS to jasmonate signalling. Nature. 2010:464:788–791. 10.1038/nature08854. 20360743 PMC2849182

[kiaf674-B62] Peng J et al COP1 positively regulates ABA signaling during Arabidopsis seedling growth in darkness by mediating ABA-induced ABI5 accumulation. Plant Cell. 2022:34:2286–2308. 10.1093/plcell/koac073. 35263433 PMC9134052

[kiaf674-B63] Pombo MA et al Transcriptome-based identification and validation of reference genes for plant-bacteria interaction studies using Nicotiana benthamiana. Sci Rep. 2019:9:1632. 10.1038/s41598-018-38247-2. 30733563 PMC6367355

[kiaf674-B64] Powers SK et al Nucleo-cytoplasmic partitioning of ARF proteins controls auxin responses in *Arabidopsis thaliana*. Mol Cell. 2019:76:177–190.e5. 10.1016/j.molcel.2019.06.044. 31421981 PMC6778021

[kiaf674-B65] Roitinger E et al Quantitative phosphoproteomics of the ataxia telangiectasia-mutated (ATM) and ataxia telangiectasia-mutated and Rad3-related (ATR) dependent DNA damage response in *Arabidopsis thaliana*. Molec Cell Proteomics. 2015:14:556–571. 10.1074/mcp.M114.040352. 25561503 PMC4349977

[kiaf674-B66] Schoonheim PJ et al 14-3-3 adaptor proteins are intermediates in ABA signal transduction during barley seed germination. Plant J. 2007:49:289–301. 10.1093/nargab/lqad041. 17241451

[kiaf674-B67] Seger R, Krebs E. The MAPK signaling cascade. FASEB J. 1995:9:726–735. 10.1096/fasebj.9.9.7601337. 7601337

[kiaf674-B68] Shao Y et al The YDA-MKK4/MKK5-MPK3/MPK6 cascade functions downstream of the RGF1-RGI ligand–receptor pair in regulating mitotic activity in root apical meristem. Mol Plant. 2020:13:1608–1623. 10.1016/j.molp.2020.09.004. 32916336

[kiaf674-B69] Sutherland C . What are the bona fide GSK3 substrates? Int J Alzheimers Dis. 2011:2011:505607. 10.4061/2011/505607. 21629754 PMC3100594

[kiaf674-B70] Takahashi Y et al MAP3Kinase-dependent SnRK2-kinase activation is required for abscisic acid signal transduction and rapid osmotic stress response. Nat Commun. 2020:11:12. 10.1038/s41467-019-13875-y. 31896774 PMC6940395

[kiaf674-B71] Tischer SV et al Combinatorial interaction network of abscisic acid receptors and coreceptors from *Arabidopsis thaliana*. Proc Natl Acad Sci U S A. 2017:114:10280–10285. 10.1073/pnas.1706593114. 28874521 PMC5617281

[kiaf674-B72] Umezawa T et al Genetics and phosphoproteomics reveal a protein phosphorylation network in the abscisic acid signaling pathway in Arabidopsis thaliana. Sci Signal. 2013:6:rs8. 10.1126/scisignal.2003509. 23572148

[kiaf674-B73] Varadi M et al AlphaFold protein structure database in 2024: providing structure coverage for over 214 million protein sequences. Nucleic Acids Res. 2023:52:D368–D375. 10.1093/nar/gkad1011. PMC10767828 37933859

[kiaf674-B74] Voinnet O, Rivas S, Mestre P, Baulcombe D. An enhanced transient expression system in plants based on suppression of gene silencing by the p19 protein of tomato bushy stunt virus. Plant J. 2003:33:949–956. 10.1046/j.1365-313X.2003.01676.x. 12609035

[kiaf674-B75] Wang C, Wen J, Liu Y, Yu B, Yang S. SOS2-AFP2 module regulates seed germination by inducing ABI5 degradation in response to salt stress in Arabidopsis. Biochem Biophys Res Commun. 2024:723:150190. 10.1016/j.bbrc.2024.150190. 38838447

[kiaf674-B76] Wang H, Ngwenyama N, Liu Y, Walker JC, Zhang S. Stomatal development and patterning are regulated by environmentally responsive mitogen-activated protein kinases in Arabidopsis. Plant Cell. 2007:19:63–73. 10.1105/tpc.106.048298. 17259259 PMC1820971

[kiaf674-B77] Wang P et al Quantitative phosphoproteomics identifies SnRK2 protein kinase substrates and reveals the effectors of abscisic acid action. Proc Natl Acad Sci U S A. 2013:110:11205–11210. 10.1073/pnas.1308974110. 23776212 PMC3703982

[kiaf674-B78] Wang X et al ABRE-BINDING FACTORS play a role in the feedback regulation of ABA signaling by mediating rapid ABA induction of ABA co-receptor genes. New Phytol. 2019:221:341–355. 10.1111/nph.15345. 30019753

[kiaf674-B79] Wei J, Li X, Song P, Wang Y, Ma J. Studies on the interactions of AFPs and bZIP transcription factor ABI5. Biochem Biophys Res Commun. 2022:590:75–81. 10.1016/j.bbrc.2021.12.046. 34973533

[kiaf674-B80] Xie D et al Phase separation of SERRATE drives dicing body assembly and promotes miRNA processing in Arabidopsis. Nat Cell Biol. 2021:23:32–39. 10.1038/s41556-020-00606-5. 33288888

[kiaf674-B81] Yilmaz M, Paulic M, Seidel T. Interactome of Arabidopsis Thaliana. Plants. 2022:11:350. 10.3390/plants11030350. 35161331 PMC8838453

[kiaf674-B82] Yoshida T et al *ABA- hypersensitive germination3* encodes a protein phosphatase 2C (AtPP2CA) that strongly regulates abscisic acid signaling during germination among Arabidopsis protein phosphatase 2Cs. Plant Physiol. 2006:140:115–126. 10.1104/pp.105.070128. 16339800 PMC1326036

[kiaf674-B83] Yu F, Wu Y, Xie Q. Precise protein post-translational modifications modulate ABI5 activity. Trends Plant Sci. 2015:20:569–575. 10.1016/j.tplants.2015.05.004. 26044742

[kiaf674-B84] Zhao H et al ABI5 modulates seed germination via feedback regulation of the expression of the PYR/PYL/RCAR ABA receptor genes. New Phytol. 2020:228:596–608. 10.1111/nph.16713. 32473058

[kiaf674-B85] Zhou X et al SOS2-LIKE PROTEIN KINASE5, an SNF1-RELATED PROTEIN KINASE3-type protein kinase, is important for abscisic acid responses in Arabidopsis through phosphorylation of ABSCISIC ACID-INSENSITIVE5. Plant Physiol. 2015:168:659–676. 10.1104/pp.114.255455. 25858916 PMC4453773

[kiaf674-B86] Zou M et al MPK3- and MPK6-mediated VLN3 phosphorylation regulates actin dynamics during stomatal immunity in Arabidopsis. Nat Commun. 2021:12:6474. 10.1038/s41467-021-26827-2. 34753953 PMC8578381

[kiaf674-B87] Zulawski M, Braginets R, Schulze WX. Phosphat goes kinases—searchable protein kinase target information in the plant phosphorylation site database PhosPhAt. Nucleic Acids Res. 2012:41:D1176–D1184. 10.1093/nar/gks1081. 23172287 PMC3531128

