# Supplementary material for: ABI5-binding proteins are substrates of key components in the ABA core signaling pathway affecting seeds
Source: Plant Physiol. 2025 Dec 23;200(1):kiaf674. doi: 10.1093/plphys/kiaf674 (PMC12857203; doi:10.1093/plphys/kiaf674)
Supplement: kiaf674_Supplementary_Data [file kiaf674_supplementary_data.zip › Supplementary Datafinal.pdf]

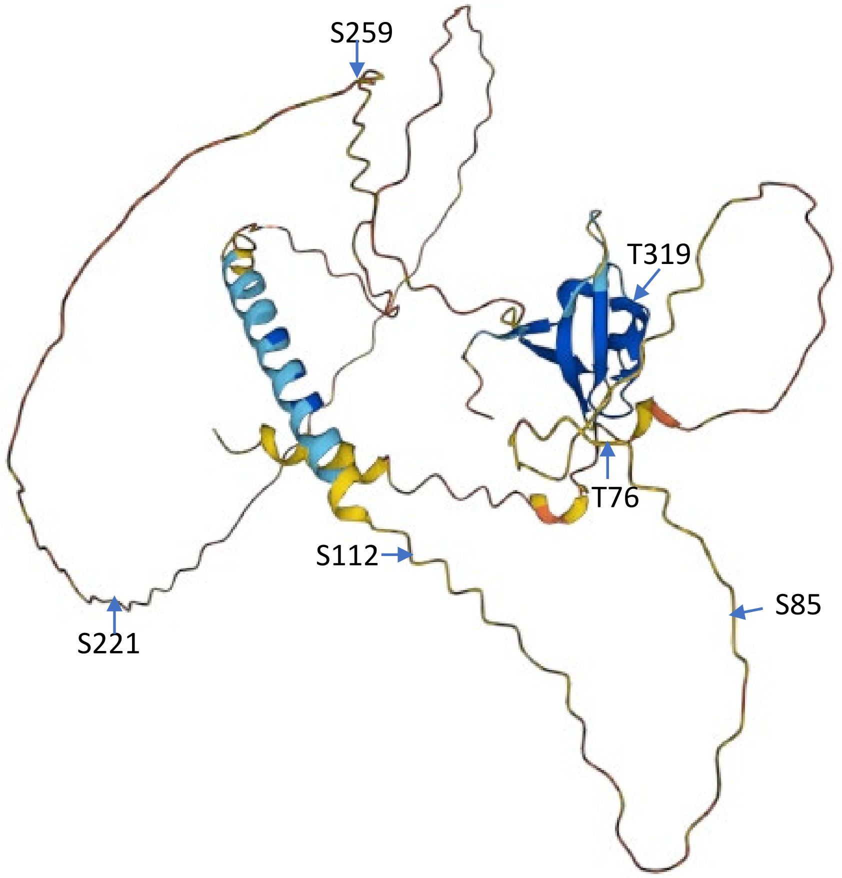


Supplementary Figure S1. Alphafold prediction of AFP2 structure (Model AF-Q9LMX5-F1) with locations of predicted SnRK2 or MPK phosphorylation sites labeled.

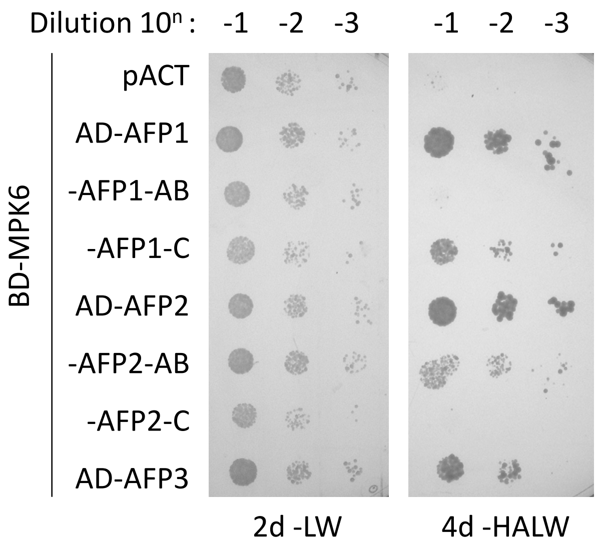


Supplementary Figure S2. Interactions between MPK6, AFPs and their subdomains detected by yeast two-hybrid assays. Following matings between haploid lines carrying the BD-MPK6 or AD-AFP fusions, the indicated diploid lines were grown overnight in media lacking leu and trp. After measuring the OD600, cultures were diluted to the same concentration, then serially diluted 10-fold three times. The diluted cultures were then replica-spotted onto selective media. The -LW plate serves as a control for accuracy of the dilutions. Growth on the -HALW plate requires interaction between the AD- and BD- fusions to activate the reporter genes complementing the *his3*- and *ade2*- mutations of these yeast.

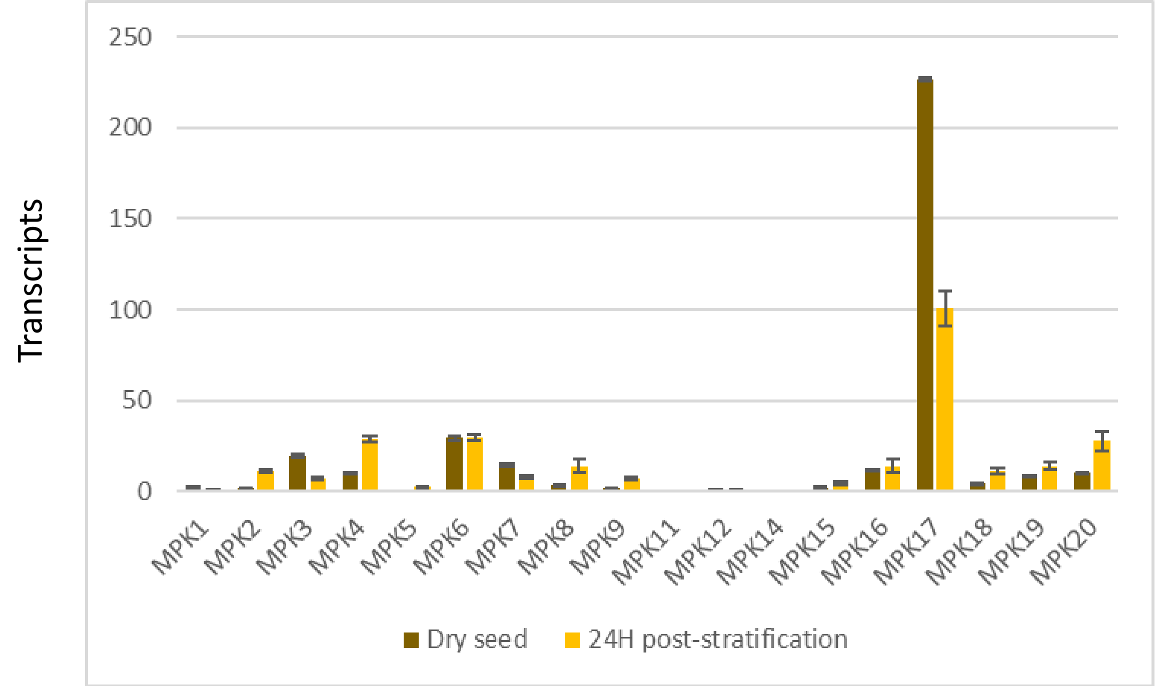


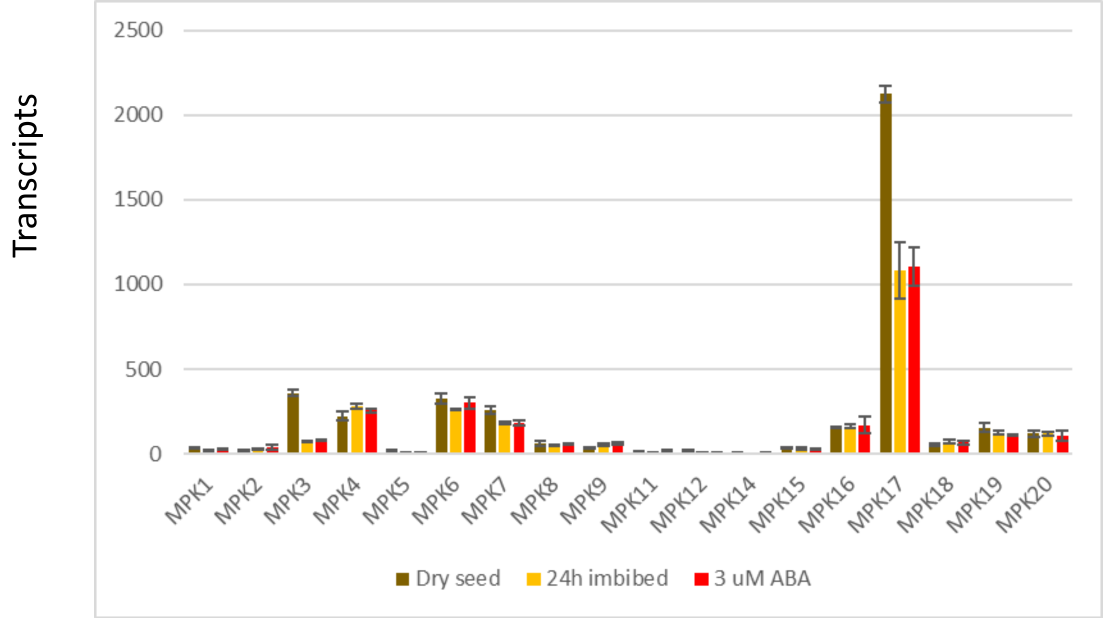


Supplementary Figure S3. Expression of MPKs in dry seeds and either 24 h post-2d-stratification (top) or after 24h imbibition in water or 3 uM ABA following 2-4 months after-ripening (bottom). Data from (Narsai et al., 2011; Nakabayashi et al., 2005) displayed as "Absolute" units on http://bar.utoronto.ca/efp//cgi-bin/efpWeb.cgi (Winter et al., 2007). Error bars represent S.D.

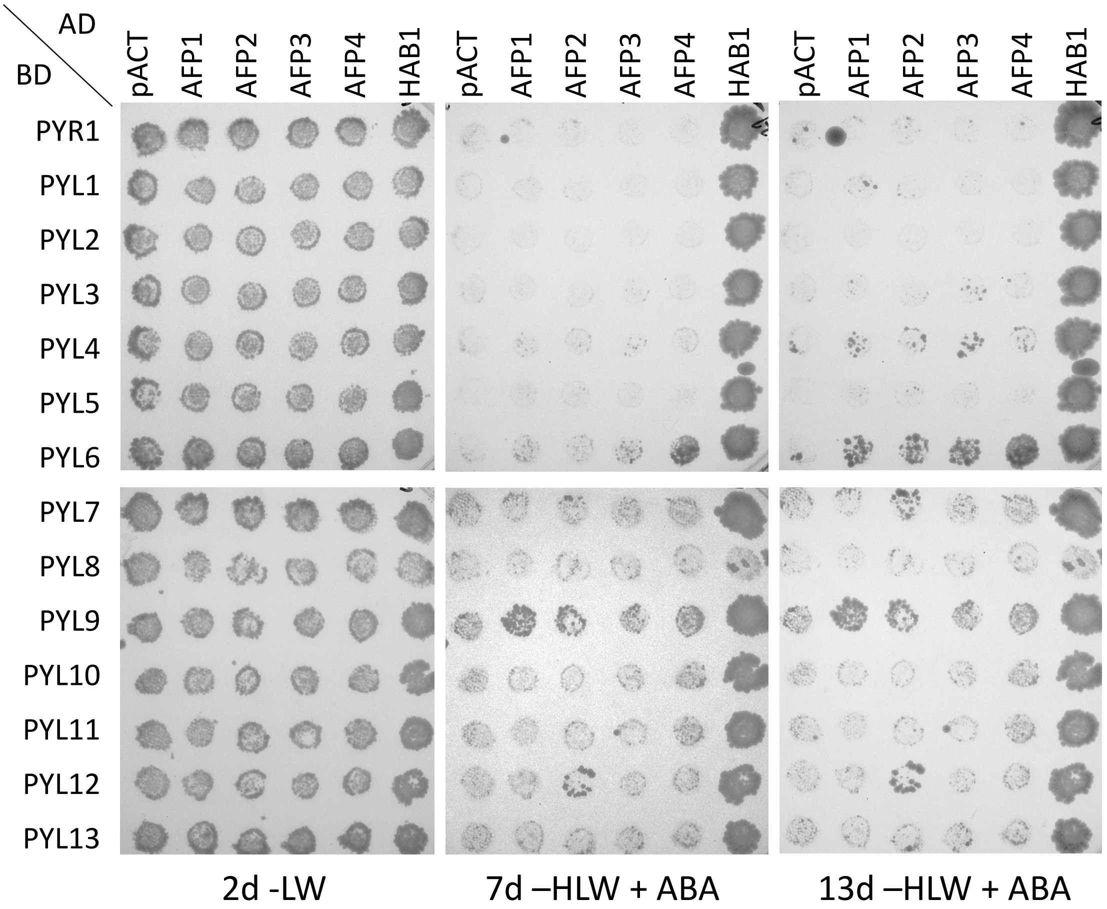


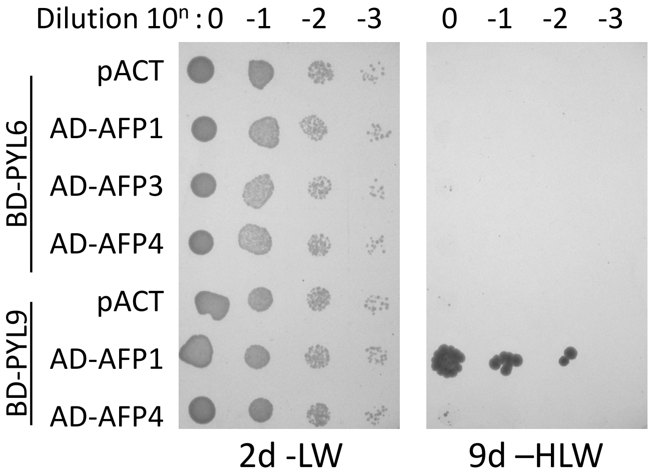


Supplementary Figure S4. Yeast two-hybrid test of interactions between ABA receptors and AFPs, presented as GAL4BD-PYR/PYL and GAL4AD-AFP fusions. The AD fusion to the PP2C HAB1 is included as a positive control, and pACT is a negative control. Fusions were combined by matings between Y187 carrying AD fusions and PJ69-4a carrying BD fusions. After overnight incubation on YPD media, yeast were replica plated onto selective media lacking leu and trp (-LW) to maintain the AD- and BD-fusion plasmids, then replica plated again to media lacking histidine (-HLW) and supplemented with ABA to score interactions between the fusion proteins. Lower panel: test of potential positives by dilution assay, as in Figure 3.

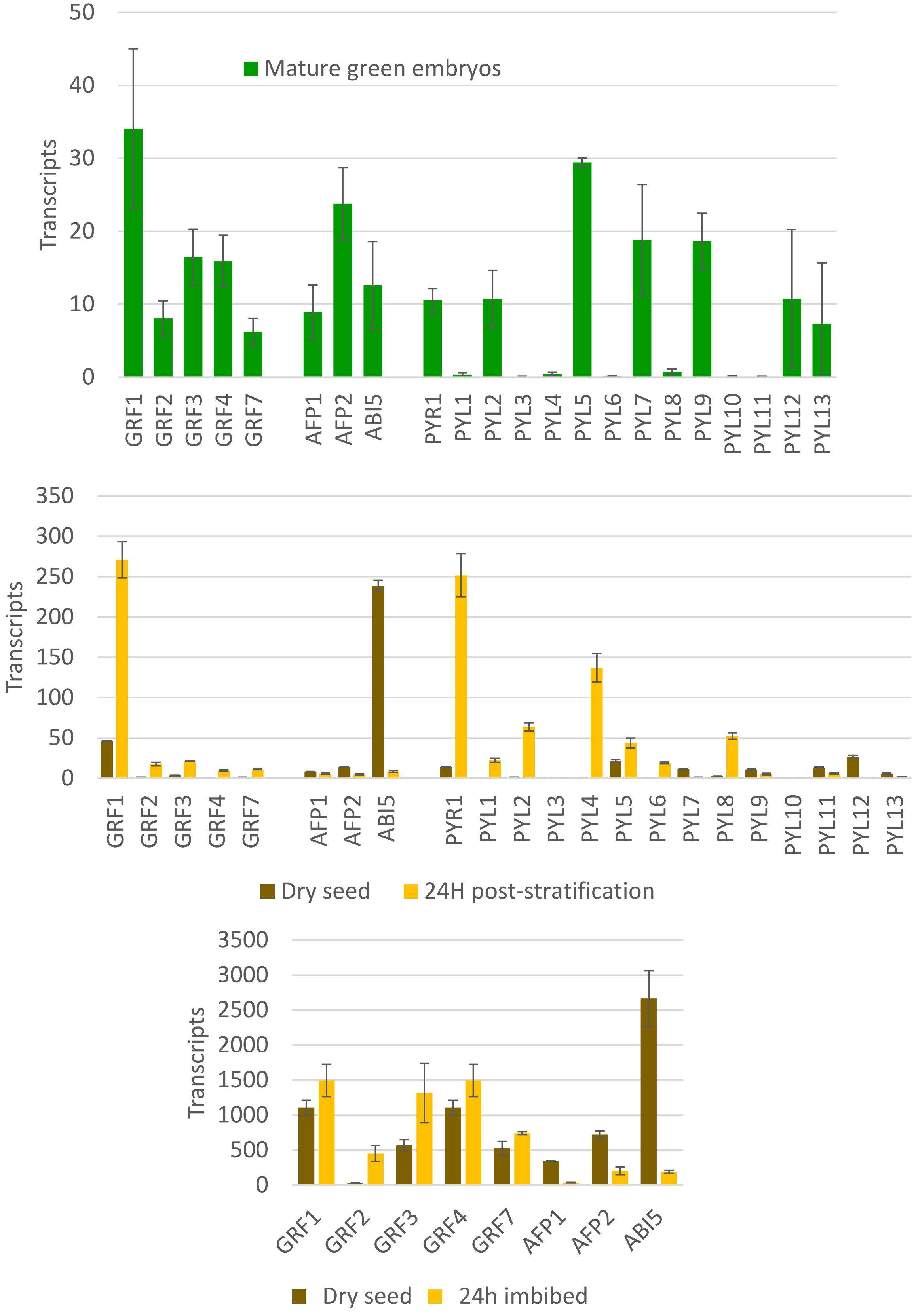


Supplementary Figure S5. Expression of PYLs, GRFs and AFPs in maturing embryos (top), dry seeds and either 24 h post-2d-stratification (middle) or after 24h imbibition in water following 2-4 months after-ripening (bottom). Data from (Narsai et al., 2011; Nakabayashi et al., 2005; Hofmann et al.,2019) displayed as "Absolute" units on http://bar.utoronto.ca/efp//cgi-bin/efpWeb.cgi (Winter et al., 2007). Error bars represent S.D.

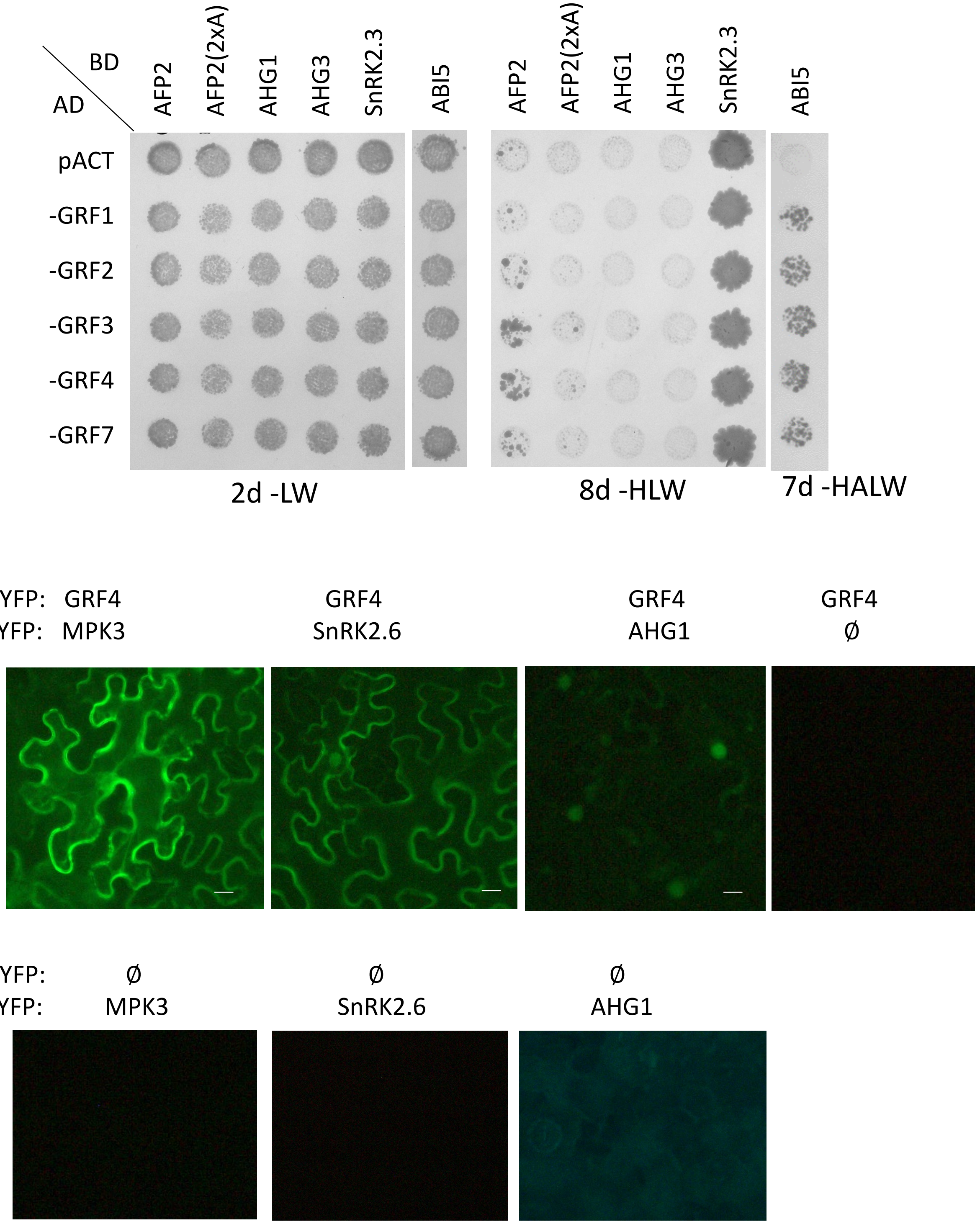


Supplementary Figure S6. Interactions between 14-3-3 proteins (GRFs) and ABA core signaling elements. Top: Yeast two-hybrid assays combining AD-fusions to 14-3-3 proteins with BD-fusions to wild-type and mutant AFP2, PP2Cs, SnRK2.3 and ABI5 by matings, as described for Supplementary Figure S4. The AFP2(2xA) mutant contains S85A and S112A substitutions.
Bottom: BiFC assays testing GRF interactions with kinases and phosphatases. Scale bars = 10 μm

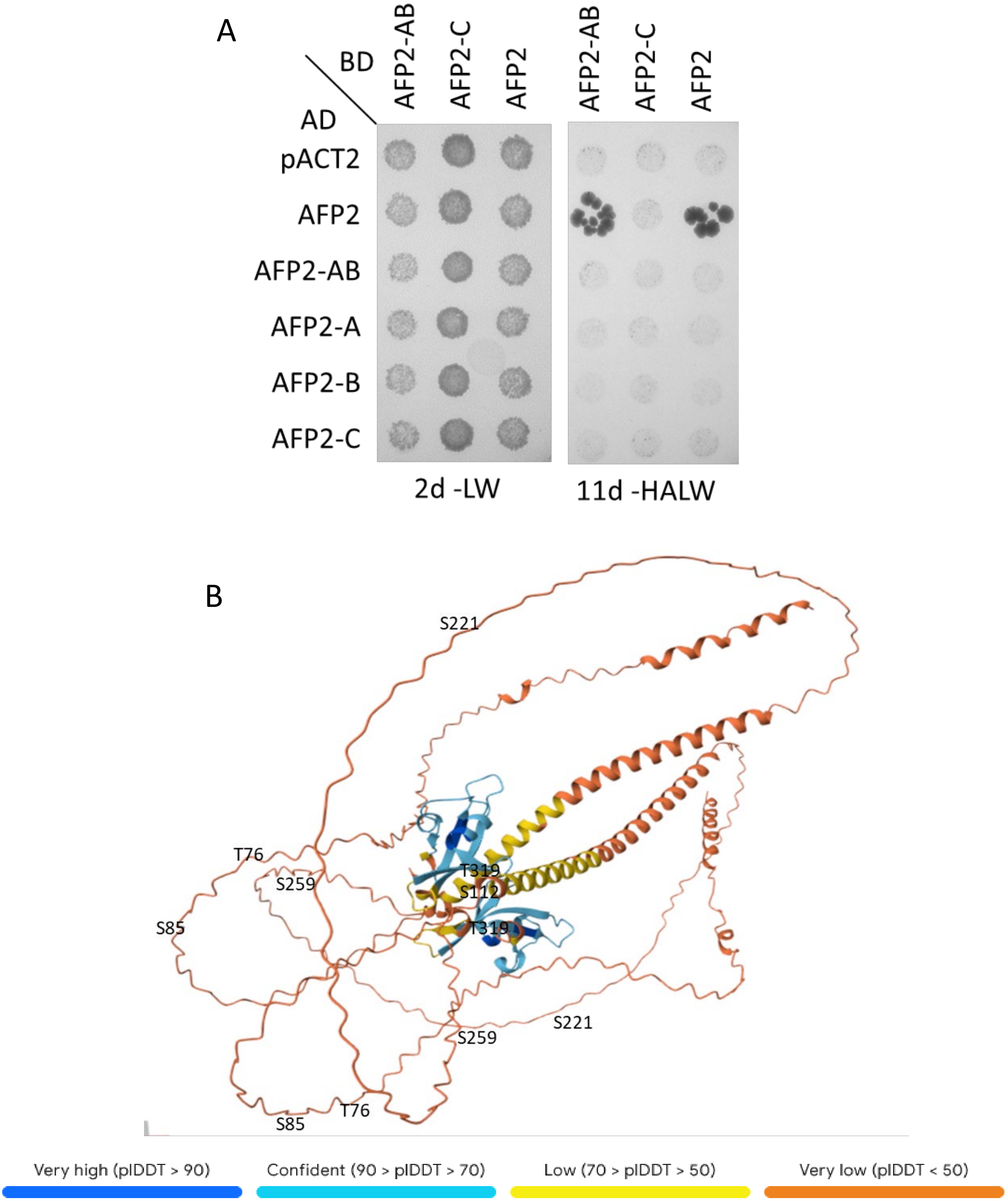


Supplementary Figure S7. Potential dimerization domains of AFP2
(A) Mapping interacting domains of AFP2 by yeast two hybrid assays, as in Supp. Fig. 2. The AFP2-AB domain includes aa 1-149 and the C domain includes aa 150-348. (B) Alphafold prediction of AFP2 dimer structure.

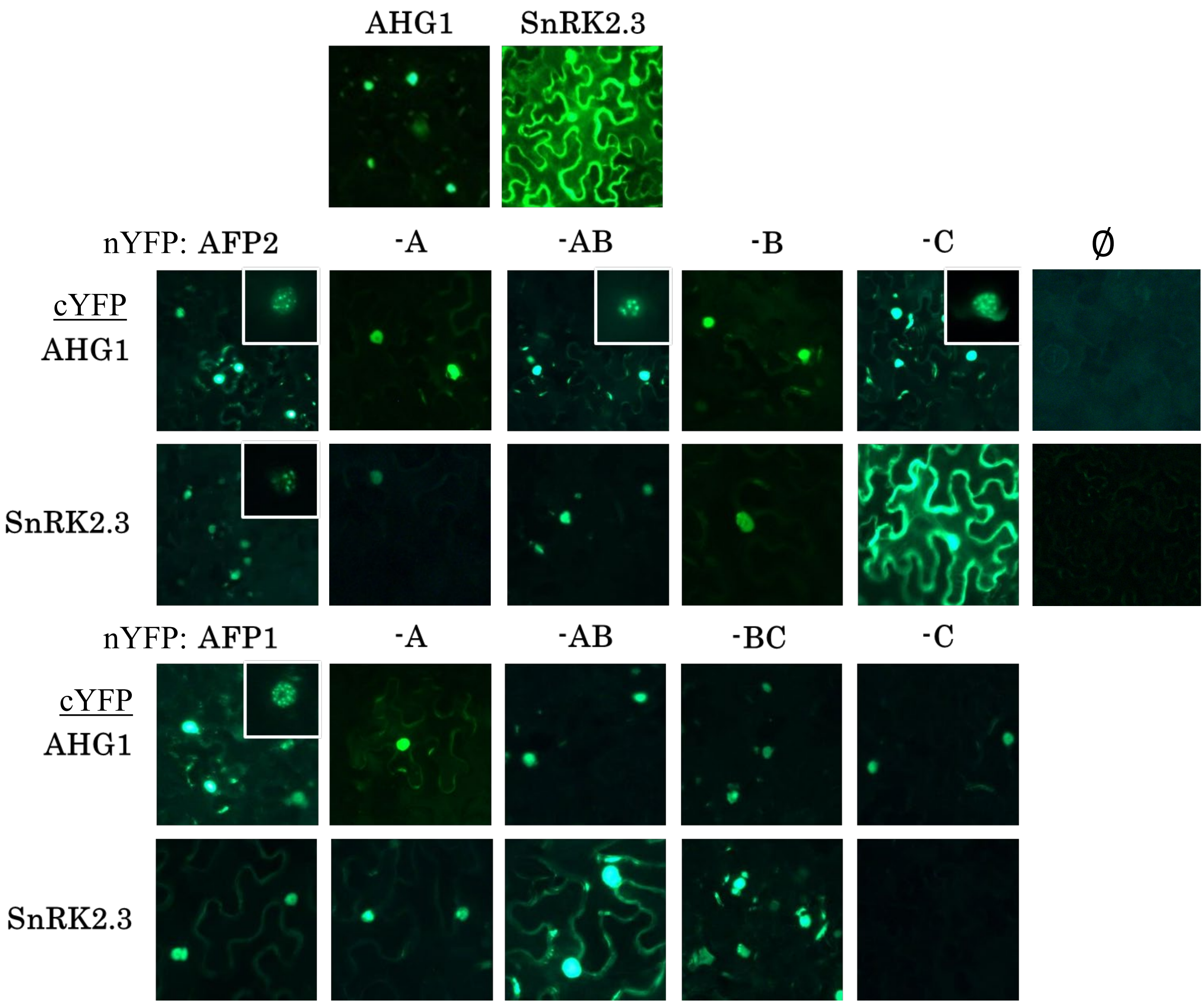


Supplementary Figure S8. Biomolecular fluorescence complementation of full length AFP2 (middle) and AFP1 (bottom) and subdomains versus ABA core signaling components in *N. benthamiana* leaves (100X magnification). Top panel shows localization of YFP-fused phosphatase and kinase for reference. Color differences are due to different versions of image capture software. Inset images show examples of nuclear speckles observed at 200X or 400X magnification. (from Erickson McNally, 2016) The images for cYFP-AHG1 with both nYFP-AFP1 and -AFP2, and cYFP-SnRK2.3 with nYFP-AFP1 are the same as those shown in Figure 4. The cYFP-AHG1/Ø control is from the same micrograph shown in Supplementary Figure S6.

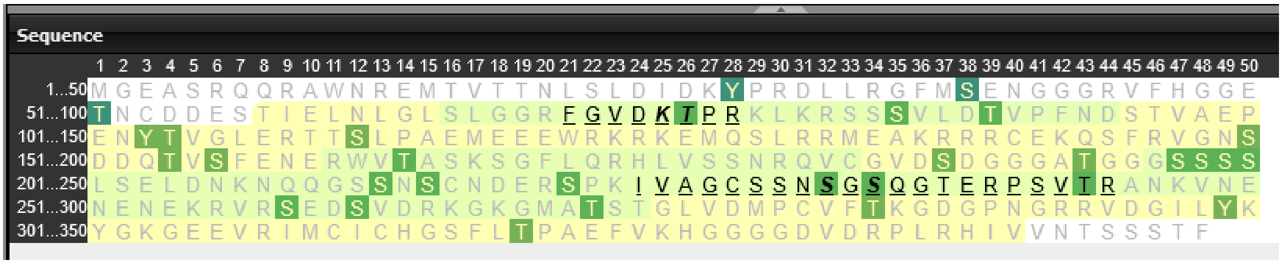


```
AFP2 :    28 YPRDLLRGFMSENGGGRVFHGGETNCDDESTIELNLGLSLGGRFGVDKTPRKLKRSSSVL 87
             +PRDLL+ F+S +   G       E    DDE   IELNLGLSLGGRFGVDK+   KL RSSSV+
AFP1 :    19 FPRDLLQRFISNSVEGE--DDDEEEDDDE--IELNLGLSLGGRFGVDKS-NKLVRSSSVV 73

AFP2 :    88 DTVP-FNDS-----------------TVAEPENYTVGLERTTSLPAEMEEEWRKRKEMQS 129
              T+P  F +                 TVA       T GL RTTSLPAE EEEWRKRKEMQ+
AFP1 :    74 VTMPLFREDHHHHQAAAMITTKVSTETVAGATRGT-GLMRTTSLPAESEEEWRKRKEMQT 132

AFP2 :   130 LRRMEAKRRRCEKQSFRVGNSDDQTVSFENERWVTASKSGFLQRHLVSSNRQVCGVDSDG 189
             LRRM AKRRR EK     VG  +        E       TAS+      +R    SS           ++
AFP1 :   133 LRRMAAKRRRSEKLRTGVGGGNSNNP--EEAATATASR----RRGRPSSGLPRWSATANK 186

AFP2 :   190 GGATGGGSSSSLSELDNKNQQ-GSSNSCNDERSPKIVAGCSSNSGSQGTERPSVTRANKV 248
              G       S+ L  L     + G    +       S    +    +  S  +       PS T+   +
AFP1 :   187 SGLLRQ-HSAGLDSLQVSGESLGGGRAAGSSSSVSELE--TKASSDEARSLPSTTQPQQE 243

AFP2 :   249 NENENEKRVRSEDSVDRKGKGMATSTGLVDMPCVFTKGDGPNGRRVDGILYKYGKGEEVR 308
                +    R+R    SVD    K        G  +MPCVFTKGDGPNG+RVDGILY+YG GEEVR
AFP1 :   244 TTTKPTNRLRRLSSVDMNMKMEPQGKGKSEMPCVFTKGDGPNGKRVDGILYRYGSGEEVR 303

AFP2 :   309 IMCICHGSFLTPAEFVKHGGGGDVDRPLRHIVVNTSS 345
             IMC+CHG FL+PA+FVKH GG   VD PLRHIVVNTSS
AFP1 :   304 IMCVCHGDFLSPADFVKHAGGPHVDHPLRHIVVNTSS 340
```

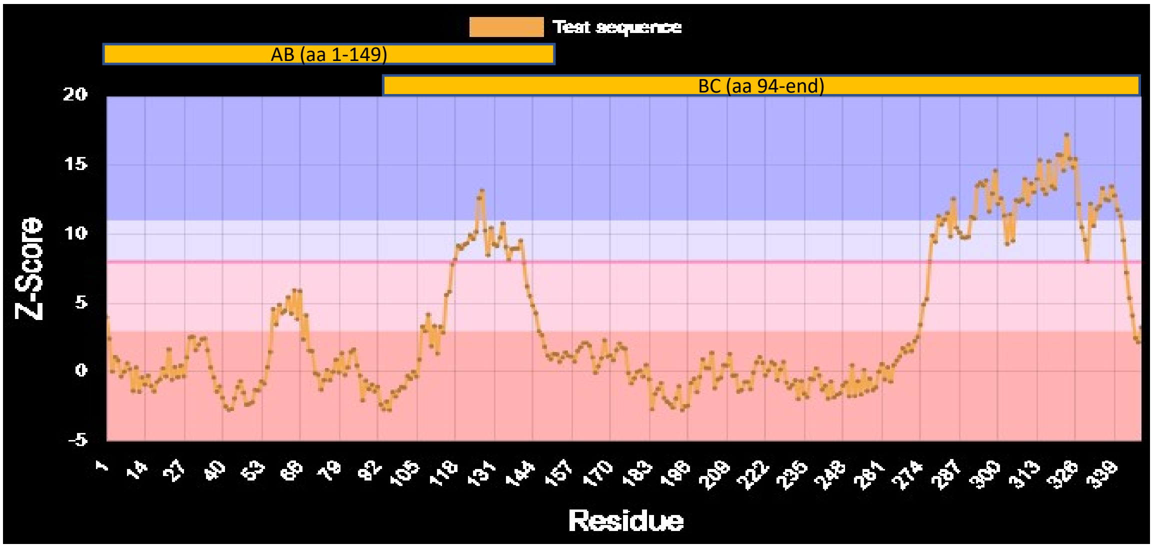


Supplementary Figure S9. Predicted phosphorylated residues and disordered regions in AFP1 and AFP2. Top: PhosPhAt shows possible phosphorylation sites of AFP1 highlighted in green, with documented sites identified by black letters. Middle: Alignment of AFP1 and AFP2. A, B and C domains are underlined in blue, green and red, respectively. Residues fitting consensus for MAPK phosphorylation are in blue, those fitting SnRK2 consensus are in red. Bottom: Disordered regions (score <8) in AFP2 predicted by Attention based DisOrder PredicTor (ADOPT) (Redl et al. 2023), with extent of the subdomain constructs shown above the disorder plot.

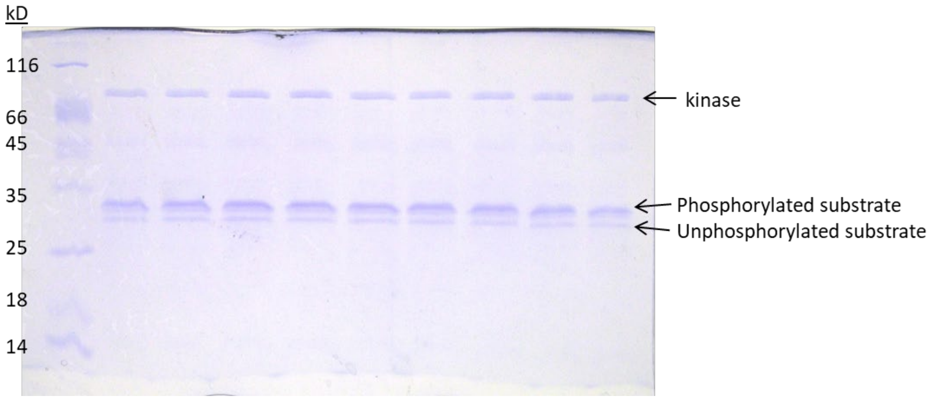


[illegible]

**10 exclusive unique peptides, 10 exclusive unique spectra, 10 total spectra, 113/192 amino acids (59% coverage)**

M G S S H H H H H H  S S G L V P R G S H  M I T S Y S I H Y T  K L S G I R P S R P  Q D P M G E A S R Q
Q R A W N R E M T V  T T N L S L D I D K  Y P R D L L R G F M  S E N G G G R V F H  G G E T N C D D E S
T I E L N L G L S L  G G R F G V D K T P  R K L K R S S S V L  D T V P F N D S T V  A E P E N Y T V G L
E R T T S L P A E M  E E E W R K R K E M  Q S L R R M E A K R  R R C E K Q S F R V  G N

| Valid | Weight | Sequence | Prob | IdentityE Score | NTT | Modifications |
|---|---|---|---|---|---|---|
| ☑ | 1.0 | (R)GSHMITSYSIHYTK(L) | 100% | 8.7 | 2 | |
| ☑ | 1.0 | (K)LSGIRPSRPQDPMGEASR(Q) | 100% | 8.6 | 2 | |
| ☑ | 1.0 | (R)EMTVTTNLSLDIDKYPR(D) | 99% | 7.8 | 2 | Oxidation (+16) |
| ☑ | 1.0 | (R)DLLRGFMSENGGGR(V) | 97% | 7.1 | 2 | Oxidation (+16) |
| ☑ | 1.0 | (R)GFMSENGGGR(V) | 99% | 8.0 | 2 | Deamidated (+1) |
| ☑ | 1.0 | (R)FGVDKTPR(K) | 98% | 7.5 | 2 | |
| ☑ | 1.0 | (R)SSSVLDTVPFNDSTVAEPENYTVGLER(T) | 99% | 7.7 | 2 | |
| ☑ | 1.0 | (R)TTSLPAEMEEEWR(K) | 100% | 8.3 | 2 | |
| ☑ | 1.0 | (R)TTSLPAEMEEEWRK(R) | 99% | 8.1 | 2 | |
| ☑ | 1.0 | (R)TTSLPAEMEEEWRKR(K) | 98% | 7.6 | 2 | Phosphoryl+STY (+80) |

Protein Sequence | Similar Proteins | Spectrum | Spectrum/Model Error | Fragmentation Table

| B | B Ions | B+2H | B-NH3 | B-H2O | AA | Y Ions | Y+2H | Y-NH3 | Y-H2O | Y |
|---|---|---|---|---|---|---|---|---|---|---|
| 1 | 102.1 | 51.5 | | 84.0 | T | 1,942.9 | 971.9 | 1,925.8 | 1,924.9 | 15 |
| 2 | 203.1 | 102.1 | | 185.1 | T | 1,841.8 | 921.4 | 1,824.8 | 1,823.8 | 14 |
| 3 | 370.1 | 185.6 | | 352.1 | S+80 | 1,740.8 | 870.9 | 1,723.8 | 1,722.8 | 13 |
| 4 | 483.2 | 242.1 | | 465.2 | L | 1,573.8 | 787.4 | 1,556.8 | 1,555.8 | 12 |
| 5 | 580.2 | 290.6 | | 562.2 | P | 1,460.7 | 730.9 | 1,443.7 | 1,442.7 | 11 |
| 6 | 651.3 | 326.1 | | 633.3 | A | 1,363.6 | 682.3 | 1,346.6 | 1,345.6 | 10 |
| 7 | 780.3 | 390.7 | | 762.3 | E | 1,292.6 | 646.8 | 1,275.6 | 1,274.6 | 9 |
| 8 | 911.4 | 456.2 | | 893.3 | M | 1,163.6 | 582.3 | 1,146.5 | 1,145.6 | 8 |
| 9 | 1,040.4 | 520.7 | | 1,022.4 | E | 1,032.5 | 516.8 | 1,015.5 | 1,014.5 | 7 |
| 10 | 1,169.4 | 585.2 | | 1,151.4 | E | 903.5 | 452.2 | 886.5 | 885.5 | 6 |
| 11 | 1,298.5 | 649.7 | | 1,280.5 | E | 774.4 | 387.7 | 757.4 | 756.4 | 5 |
| 12 | 1,484.6 | 742.8 | | 1,466.6 | W | 645.4 | 323.2 | 628.4 | | 4 |
| 13 | 1,640.7 | 820.8 | 1,623.6 | 1,622.7 | R | 459.3 | 230.2 | 442.3 | | 3 |
| 14 | 1,768.8 | 884.9 | 1,751.7 | 1,750.8 | K | 303.2 | 152.1 | 286.2 | | 2 |
| 15 | 1,942.9 | 971.9 | 1,925.8 | 1,924.9 | R | 175.1 | 88.1 | 158.1 | | 1 |

Supplementary Figure S10. Mass spectrometric analysis of phosphorylation site(s) by *in vitro* kinase reaction of His-AFP2-AB incubated with GST-SnRK2.6. Top: Coomassie-stained SDS-PAGE; the replicate phosphorylated substrate bands were excised from the gel, and sent to the UC Davis Mass Spectrometry Facility for analysis following tryptic digestion. Bottom: Summary of Mass Spec analysis by Scaffold software, including Fragmentation Table for the only peptide showing phosphorylation. The initiating Met of AFP2, at position 44 of the fusion protein, is circled in the sequence. Full dataset is available at ftp://massive-ftp.ucsd.edu/v11/MSV000099546/

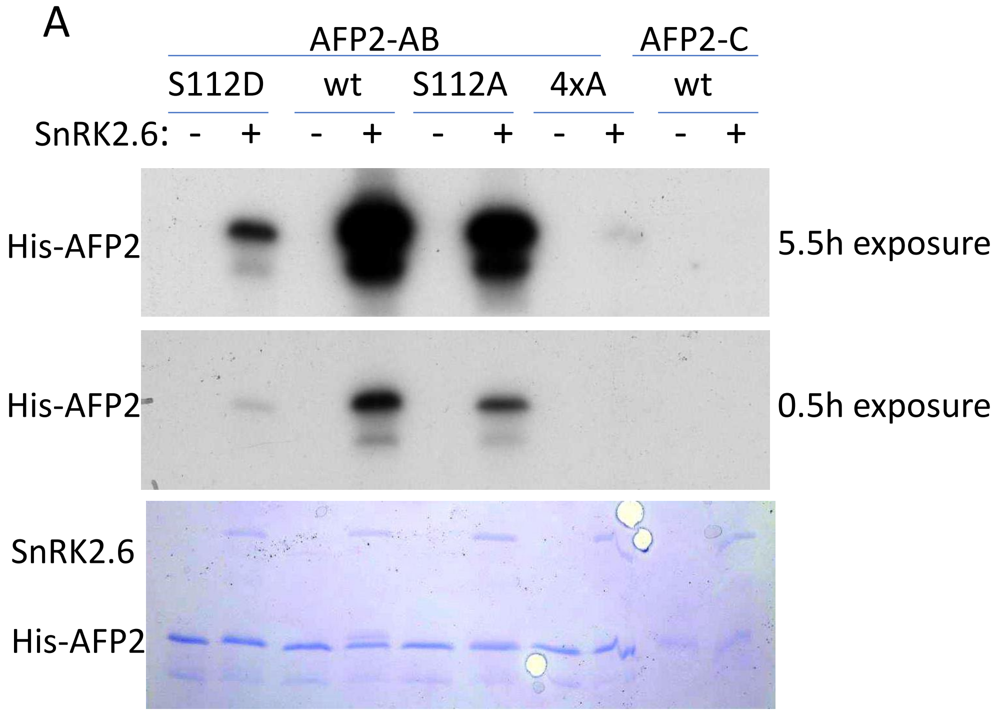


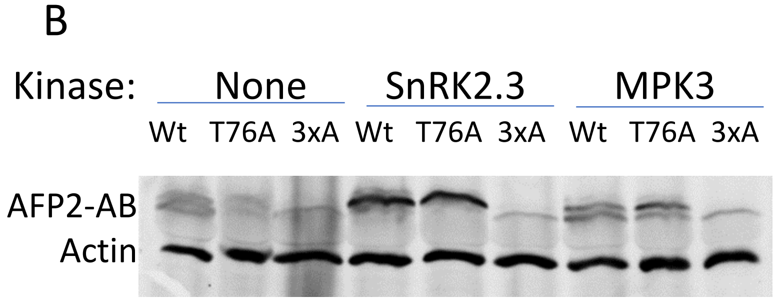


Supplementary Figure S11. Tests of potential phosphorylation sites by kinase reactions with mutant AFP substrates. (A) In vitro kinase assay comparing phosphorylation of wild-type AFP2-AB domain with phosphomutants or AFP2-C domain. Top two panels are autoradiographs of the Coomassie-stained gel shown in the bottom panel. Affinity-purified GST-SnRK2.6 was pre-activated, then reactions set up as pool before aliquoting for combination with His-AFP2 substrates. Coomassie shows loading of AB fusions and SnRK. “4xA” = (S85,T111,S112,S129)A; as shown in Fig. 5E, (S85,S112)A gives the same result as the 4xA mutant.
(B) Comparison of wild type and phosphomutant YFP-AFP2-AB domain mobility when transiently expressed in *N. benthamiana*, either alone or in combination with either Myc-SnRK2.3 or Myc-MPK3. “3xA” = (T76,S85,S112)A

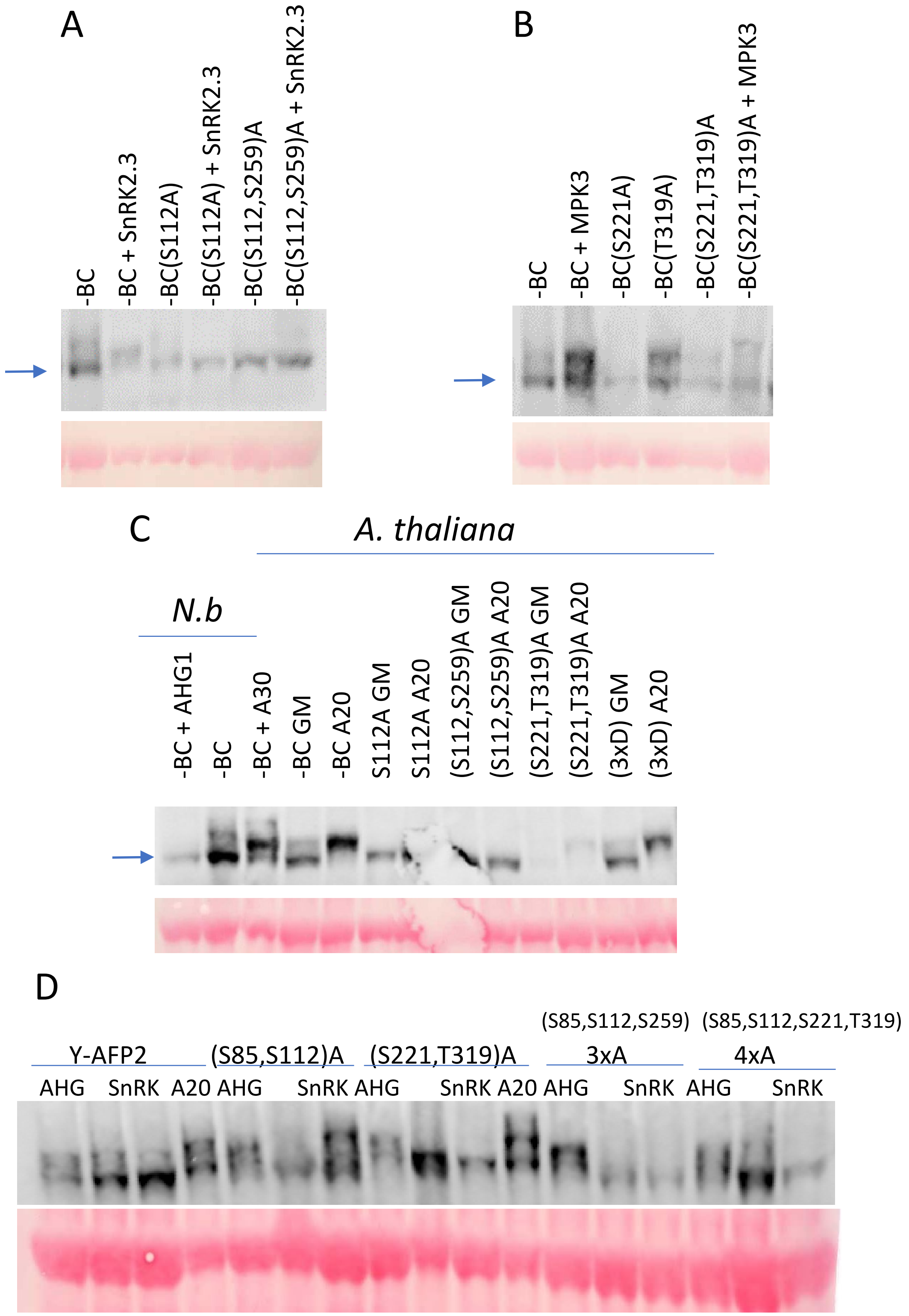


Supplementary Figure S12. Comparison of phosphorylation status of AFP2-BC domain and full-length AFP2 by Phos-tag gels. YFP-AFP2-BC fusions were expressed transiently in *N.benthamiana* (A,B,C) or stably in *A.thaliana* (C) Fusions were expressed alone or in combination with Myc-SnRK2.3 (A), Myc-MPK3 (B) or Myc-AHG1 (C). ABA or GM incubations were as described in Fig. 6AB. The "3xD" mutant is altered at S112, S221, and T319. The unphosphorylated AFP2-BC domain is identified by the arrows. Full-length AFP2 fusions were expressed transiently alone or in combination with Myc-AHG1 or Myc-SnRK2.3 in *N.benthamiana* (D).

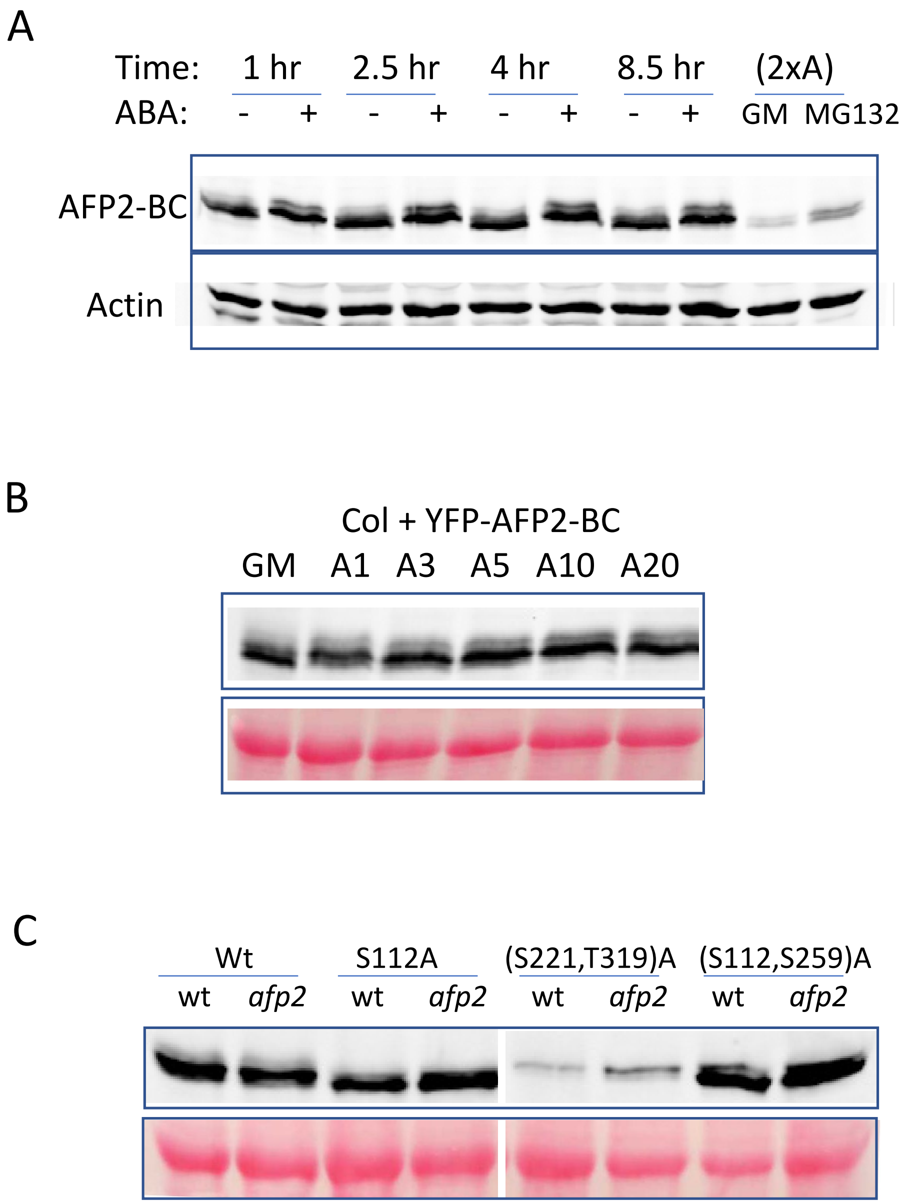


Supplementary Figure S13. Effects of ABA, mutations and genetic background on expression and mobility of YFP-AFP2-BC transgenes in stably transformed *A. thaliana.*
(A) Timecourse of changes in mobility of wild-type AFP2-BC in response to 20 μM ABA.
(B) Dose response of ABA-induced changes in mobility of wild-type AFP2-BC domain in stably transformed *A. thaliana* incubated for 6 hrs in GM with or without ABA ranging from 1-20 μM.
(C) Expression of wild-type (wt) YFP-AFP2-BC and transgenes with the indicated mutations in wt and *afp2* seedlings following germination and 7d growth on minimal media supplemented with 3 μM ABA. Upper panels are immunoblots probed with anti-GFP. Lower panels in B and C are the RbcL bands seen on Ponceau stained filters.

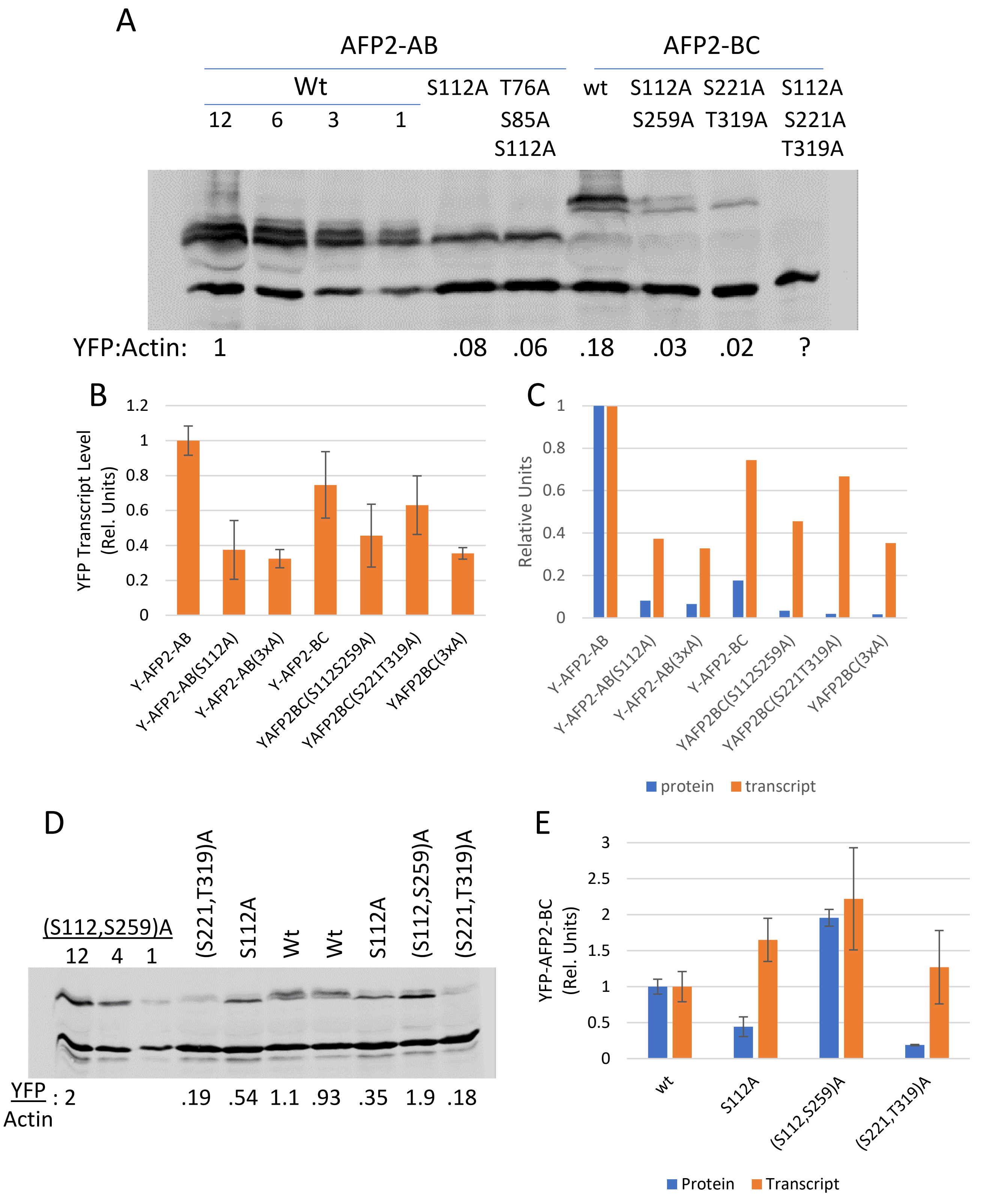


Supplementary Figure S14. Comparison of protein and transcript levels for AFP2-AB or AFP2-BC domain fusions. Transient expression in *N. benthamiana* (A-C) or stable transformants in *A. thaliana* (D,E). The standard series of wt AFP2-AB shows the volume of extract loaded for the standard curve. Amounts of YFP fusion protein were normalized relative to actin. YFP-fusion transcripts were normalized relative to these stably expressed endogenous genes: PP2A (Niben101Scf09716g01002.1) of *N.benthamiana* and the geometric mean of PP2AA3 (At1g13320) and AP2M (At5g46630) of Arabidopsis. Error bars represent S.D. of at least two biological samples.

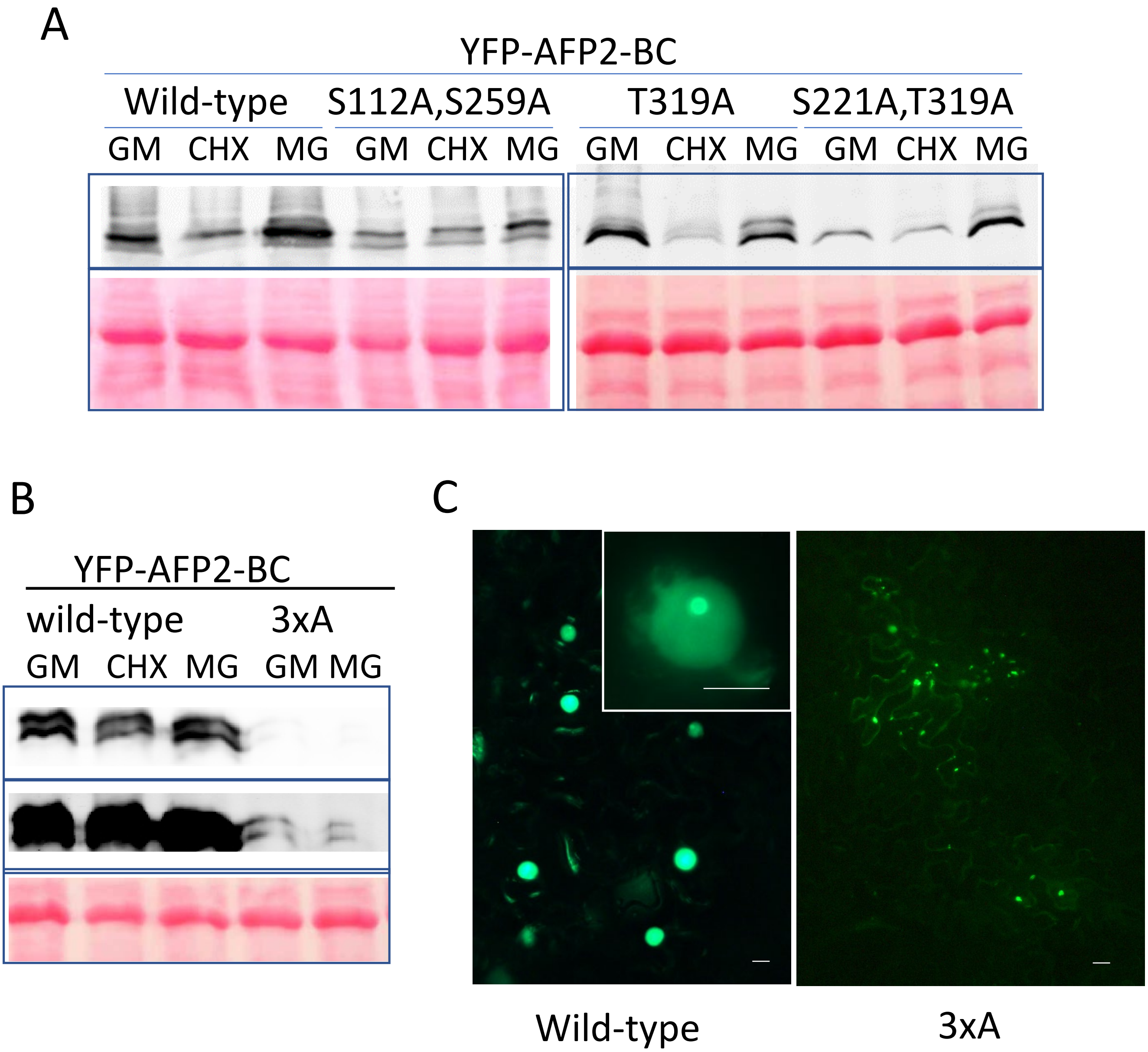


Supplementary Figure S15. Comparison of stability and accumulation of transiently expressed wild type, double (S112A,S259A or S221A,T319A) or triple (3xA=S112A,S221A,T319A) mutant AFP2-BC domain fusions to YFP. Following infiltration, *N. benthamiana* leaf discs were infiltrated with GM (0.5x MS medium) with or without 20 uM cycloheximide (CHX) or MG132( MG), and incubated 6 hrs prior to harvest for extraction. Immunoblots were probed with anti-GFP (top panels) after staining with Ponceau (lower panels) (A,B). Micrographs of *N. benthamiana* leaves used for assay in B, taken with 10x objective on an Olympus AX70 epifluorescence microscope (C). Gain was set at 11.9 for 3xA mutant vs. 2.0 for wild-type. Inset is wild-type nucleus taken with 40x objective. Scale bars = 10 μm for wild-type, 20 μm for 3xA mutant

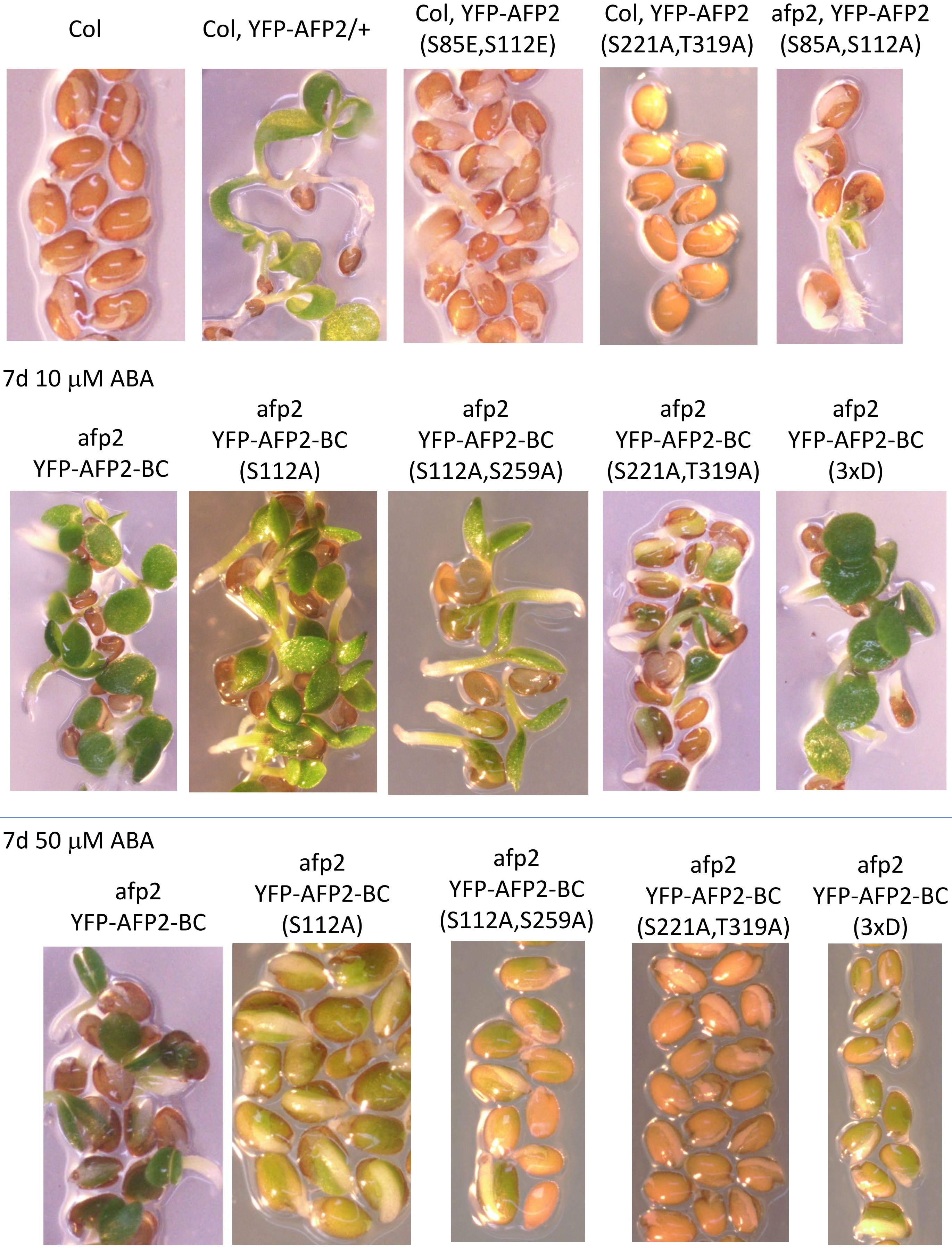


Supplementary Figure S16. Germination phenotypes of wild type (Col) and transgenic seeds expressing the indicated YFP-AFP2 fusions after 7d incubation on either 10 or 50 μM ABA.

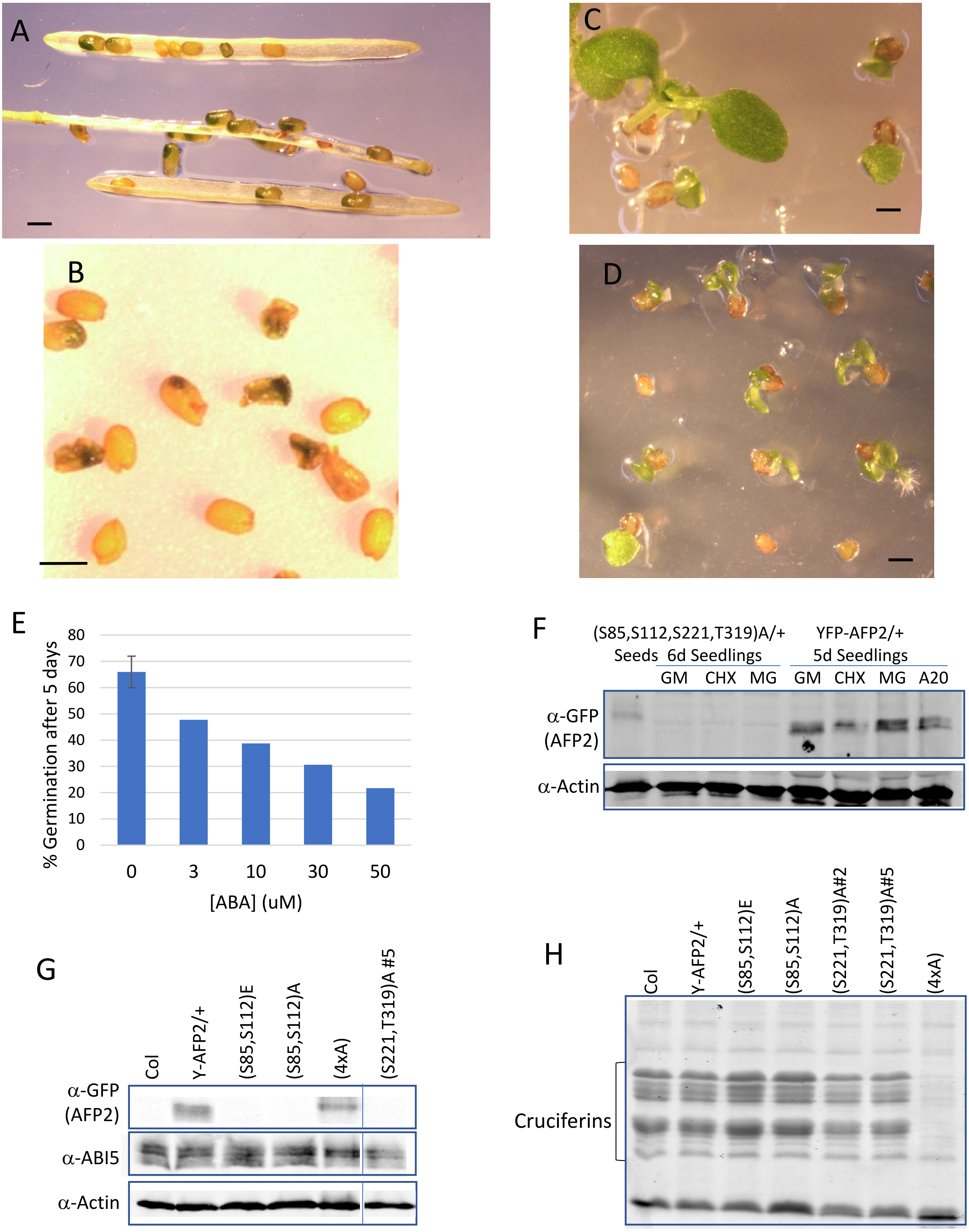
A
B
C
D
E
% Germination after 5 days
80
70
60
50
40
30
20
10
0
0
3
10
30
50
[ABA] (uM)
F
(S85,S112,S221,T319)A/+
Seeds 6d Seedlings
GM CHX MG
YFP-AFP2/+
5d Seedlings
GM CHX MG A20
α-GFP
(AFP2)
α-Actin
G
Col
Y-AFP2/+
(S85,S112)E
(S85,S112)A
(4xA)
(S221,T319)A #5
α-GFP
(AFP2)
α-ABI5
α-Actin
H
Col
Y-AFP2/+
(S85,S112)E
(S85,S112)A
(S221,T319)A#2
(S221,T319)A#5
(4xA)
Cruciferins

Supplementary Figure S17. Transgenic line hemizygous for 35S-YFP-AFP2(S85,S112,S221,T319)A fails to complete seed maturation. (A) Maturing silique segregating green seeds. (B) Dry seeds with green embryos are desiccation intolerant and shrunken. (C) Comparison of seedlings from wild type siblings (left) and green seeds (right) removed from maturing siliques prior to desiccation. (D) Some green seeds fail to develop roots on minimal media. (E) Transgenic seeds are highly resistant to ABA-inhibition of germination. (F) Immunoblot comparing accumulation of wild type and mutant AFP2 fusions in seedlings following 6h incubation in GM with or without 100 uM CHX or MG132, or 20 uM ABA. Dry seed protein extracts of the indicated genotypes were separated on 10% SDS-PAGE for immunoblots (G) or 15% SDS-PAGE to resolve storage proteins (cruciferins) (H). “4xA” = 35S-YFP-AFP2(S85,S112,S221,T319)A. Scale bars = 0.5 mm

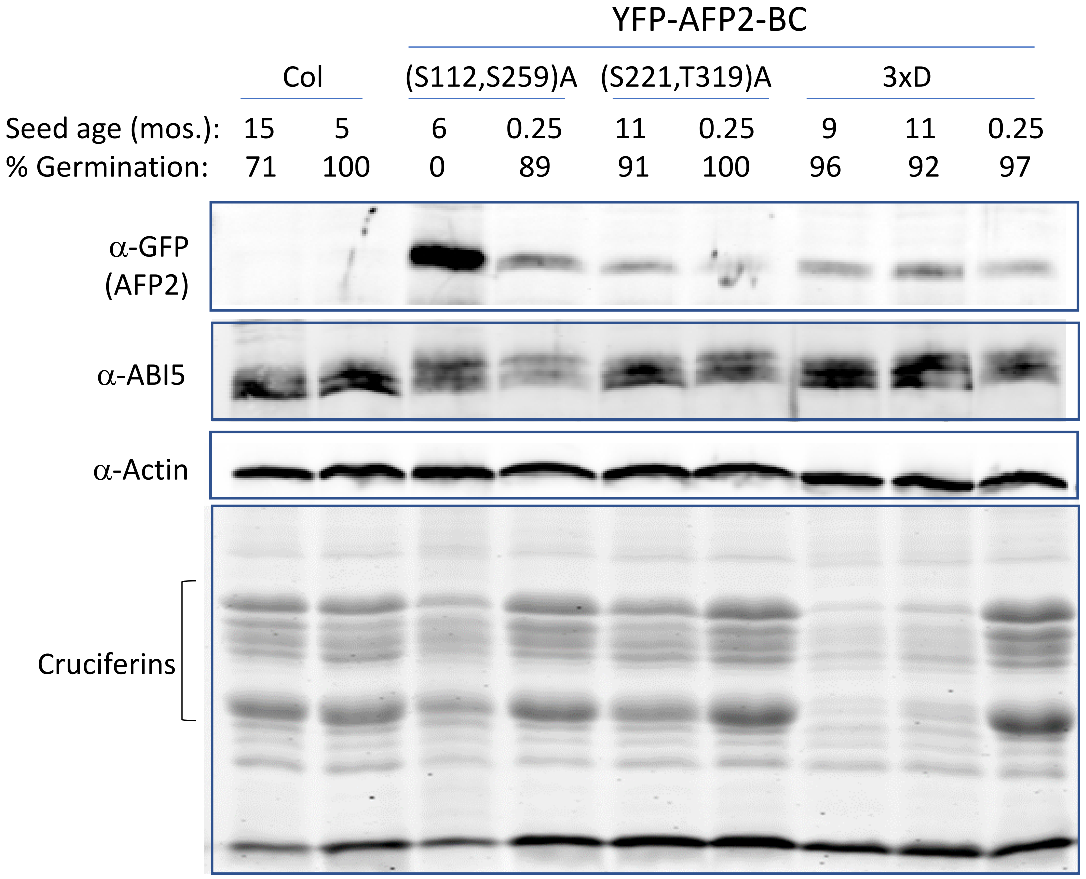


Supplementary Figure S18. Effects of phosphomutant or phosphomimetic YFP-AFP2 fusions on storage protein accumulation and seed longevity. Dry seed protein extracts of the indicated genotypes and ages were separated on 10% SDS-PAGE for immunoblot probed with the indicated antibodies or 15% SDS-PAGE for resolution of storage proteins (Cruciferins). Germination was scored at 4d post-stratification incubation on minimal nutrient media.

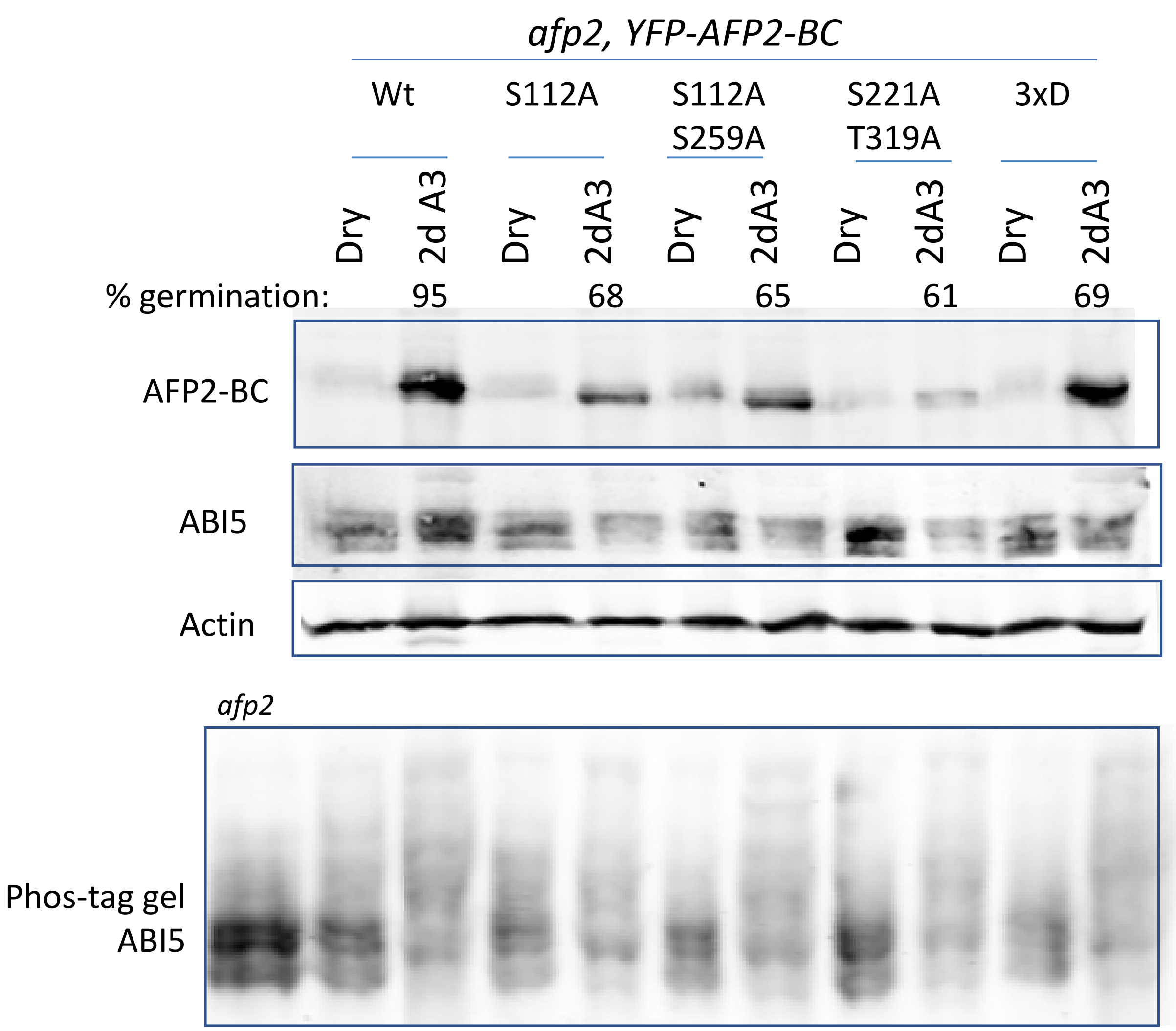


Supplementary Figure S19. Effect of ABA on accumulation and phosphorylation of ABI5 in seeds with wild type or mutant YFP-AFP2-BC fusions in an *afp2* mutant background. Protein extracts were separated on 10% SDS-PAGE (upper panels) or a 7.5% Phos-tag gel, then transferred to filters for immunoblots. Far left lane on the Phos-tag gel contains dry seed extract from th *afp2* progenitor line. Germination on minimal media supplemented with 3 µM ABA (A3) was scored just prior to harvest.

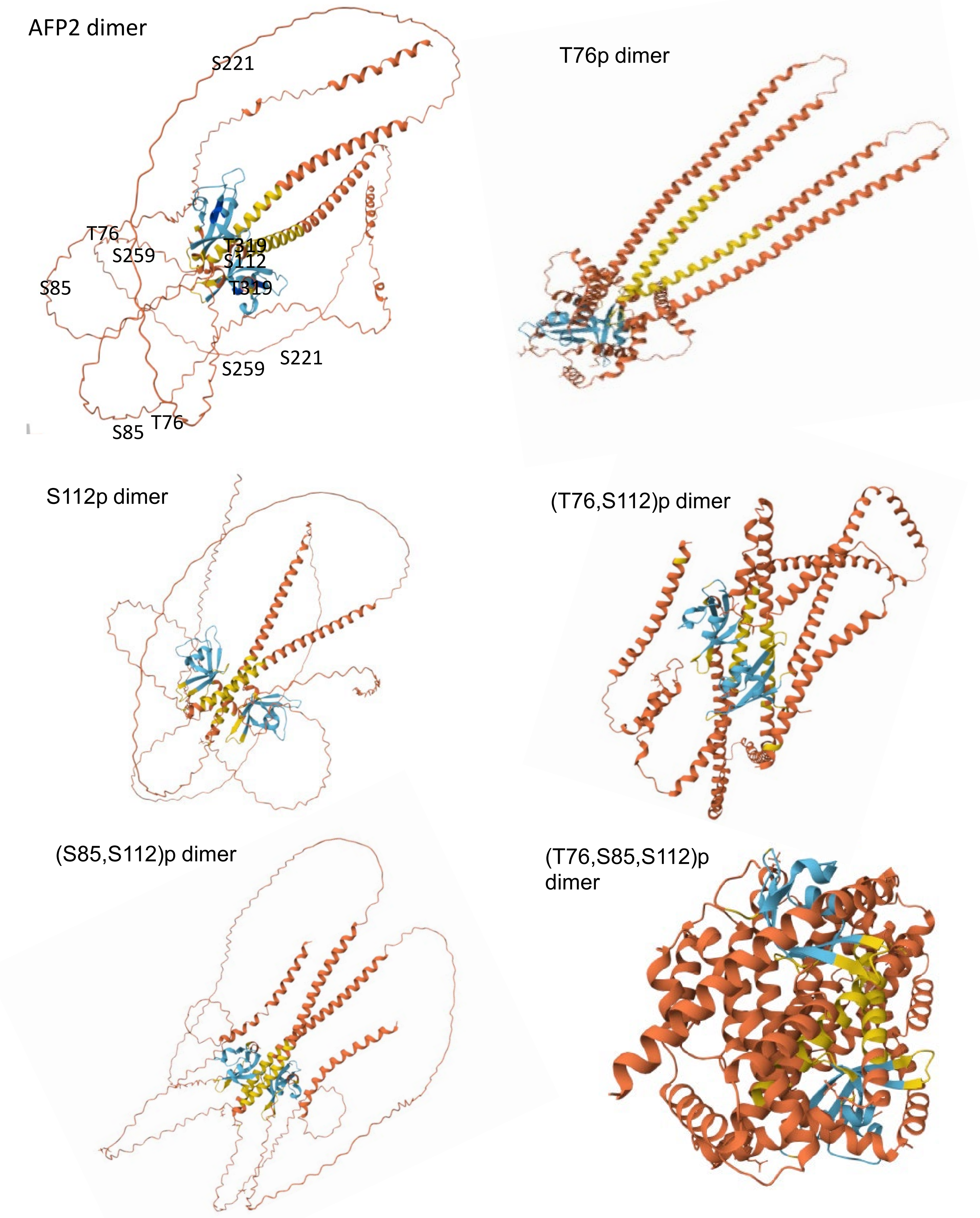


Supplementary Figure S20. Alphafold3 predictions of AFP2 dimer structure when phosphorylated, or blocked from phosphorylation, at residues tested in this work (Abramson et al., 2024).

(S85,S112,S259)p dimer

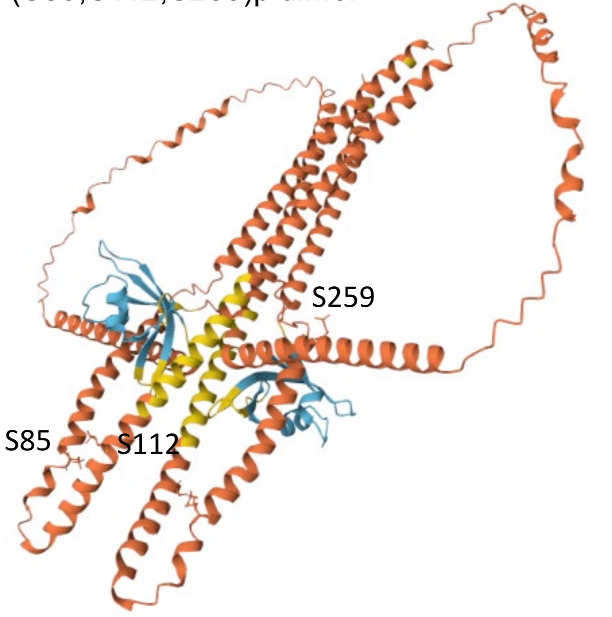


(T76,S85,S112,S259)p dimer

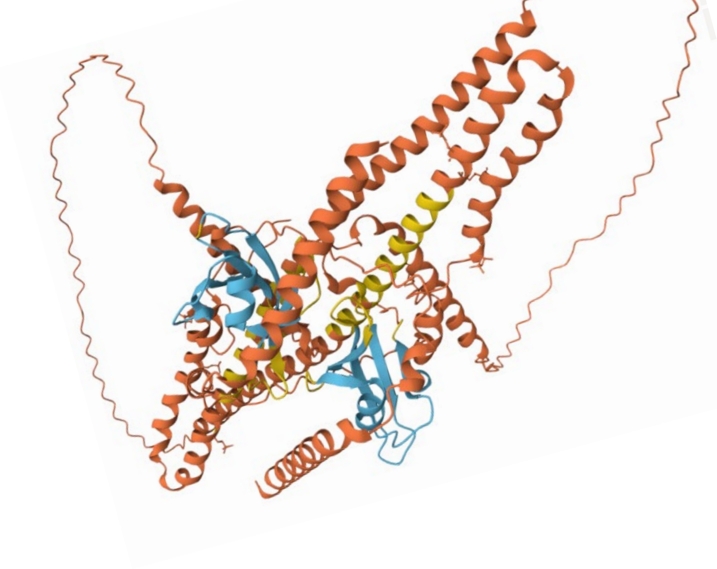

(S221,T319)p dimer

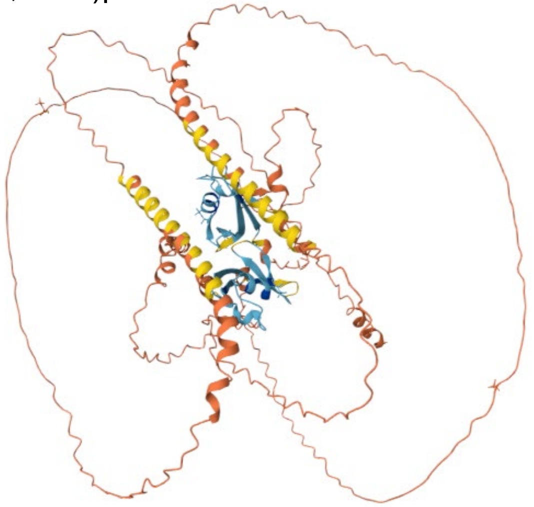

(T76, S221,T319)p dimer

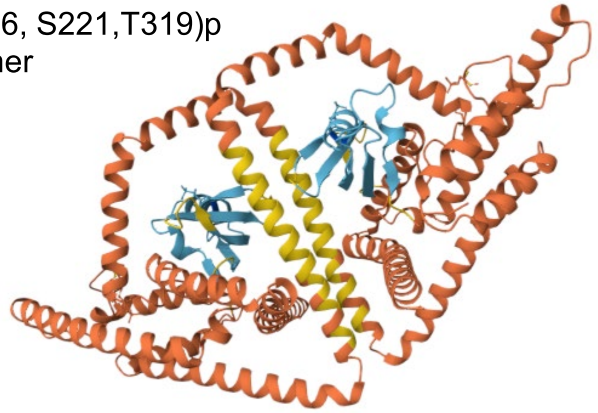

(S85,S112,S221,T319)p dimer

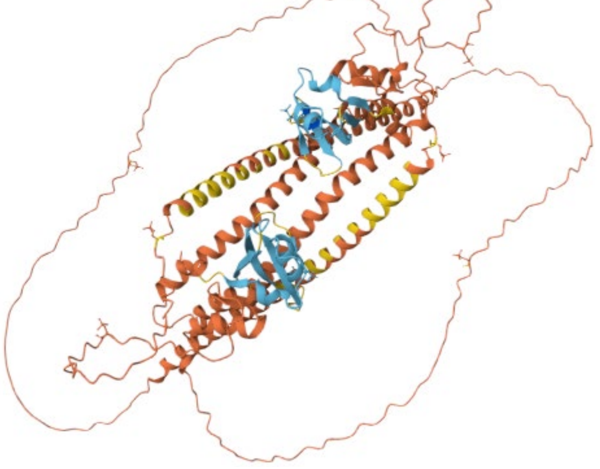

(T76,S85,S112,S221,T319)p dimer

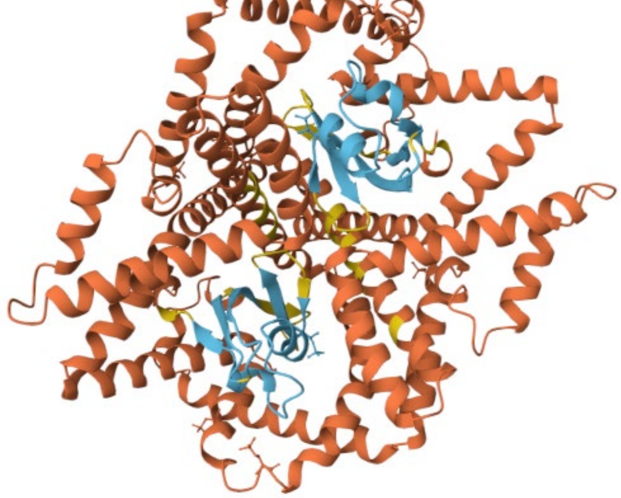

AFP2(S85,S112)A dimer

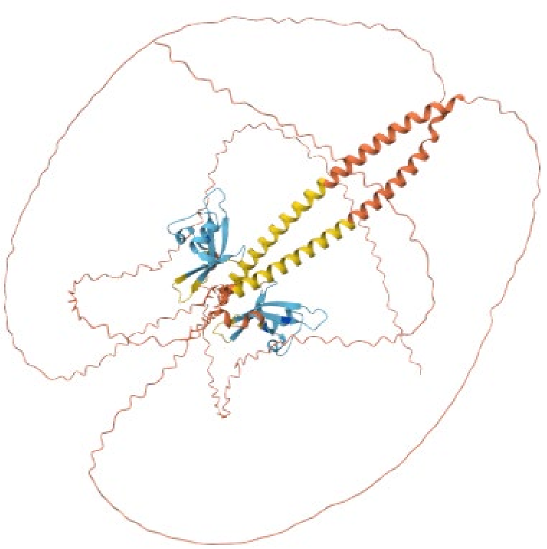

AFP2(T76,S85,S112)A dimer

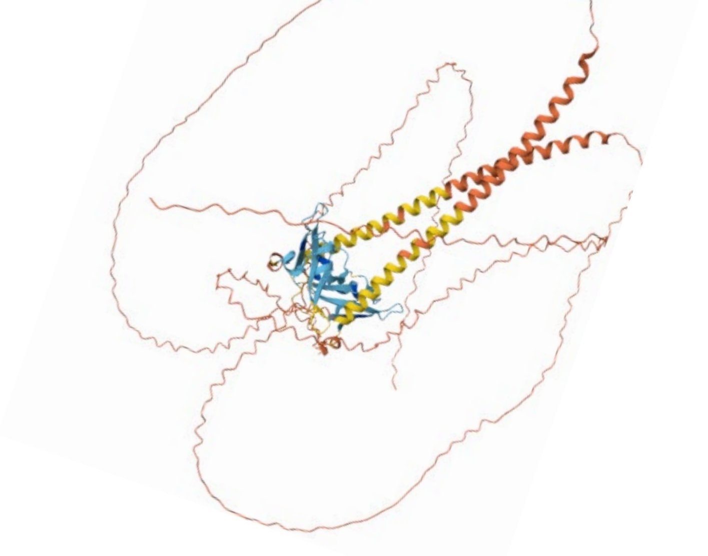

AFP2(S85,S112,S259)A dimer

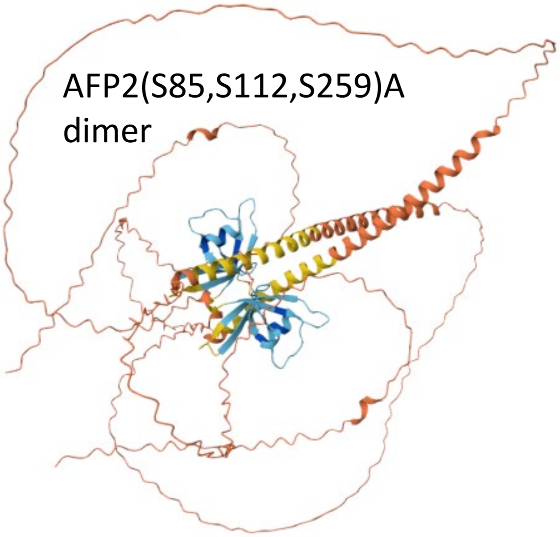

AFP2(S85,S112,S221,T319)A dimer

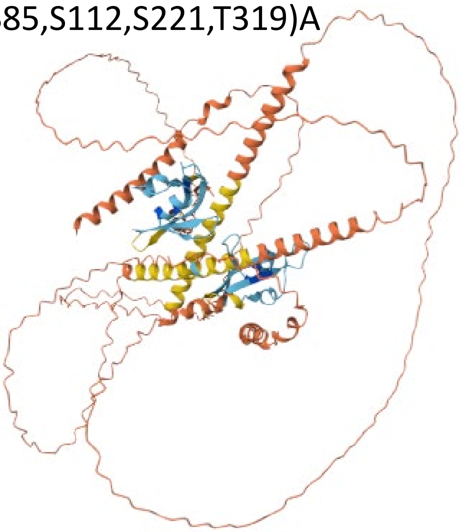

AFP2_T76p(S85,S112,S259)A dimer

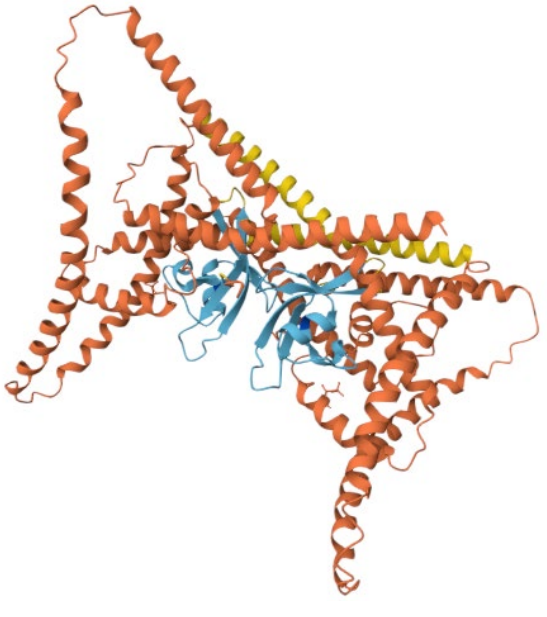

AFP2_T76p(S85,S112,S221,T319)A dimer

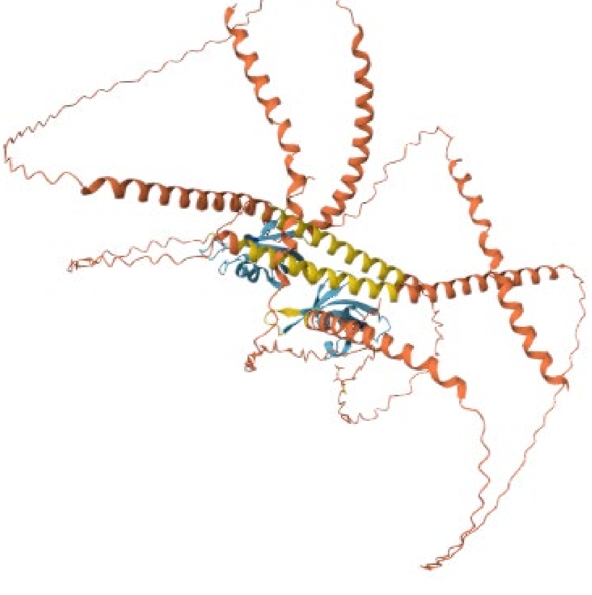

**References**


Abramson J et al. 2024. Accurate structure prediction of biomolecular interactions with AlphaFold 3. Nature. 630:493–500. .

Erickson McNally BJ-A. 2016. Expression patterns of legume-specific cell wall proteins and their genomic organization in *Medicago truncatula* and investigating the relationship between post-translational modification and AFP2 function [PhD thesis]. University of California.

Hofmann F, Schon MA, Nodine MD. 2019. The embryonic transcriptome of *Arabidopsis thaliana*. Plant Reprod. 32:77–91. https://doi.org/10.1007/s00497-018-00357-2.

Nakabayashi K, Okamoto M, Koshiba T, Kamiya Y, Nambara E. 2005. Genome-wide profiling of stored mRNA in Arabidopsis thaliana seed germination: epigenetic and genetic regulation of transcription in seed. Plant J. 41:697–709. https://doi.org/10.1111/j.1365-313X.2005.02337.x.

Narsai R, Law SR, Carrie C, Xu L, Whelan J. 2011. In-depth temporal transcriptome profiling reveals a crucial developmental switch with roles for RNA processing and organelle metabolism that are essential for germination in Arabidopsis. Plant Physiol. 157:1342–1362. https://doi.org/10.1104/pp.111.183129.

Redl I et al. 2023. ADOPT: intrinsic protein disorder prediction through deep bidirectional transformers. NAR Genom Bioinform. 5(2):lqad041. https://doi.org/10.1093/nargab/lqad041.

Winter D et al. 2007. An “Electronic Fluorescent Pictograph” browser for exploring and analyzing large-scale biological data sets. PLoS One. 2:e718. https://doi.org/10.1371/journal.pone.0000718.
